# Host Exploitation by Cuckoos in China: A Review and Real‐Time Tracking Program for Parasitism Records

**DOI:** 10.1111/1749-4877.13009

**Published:** 2025-07-17

**Authors:** Tao Liu, Canchao Yang

**Affiliations:** ^1^ Ministry of Education Key Laboratory for Ecology of Tropical Islands, College of Life Sciences Hainan Normal University Haikou China

**Keywords:** avian brood parasitism, coevolution, cuckoo diversity, egg polymorphism, host diversity

## Abstract

China stands as a global hotspot for cuckoo diversity and their avian hosts, presenting an unparalleled natural laboratory for investigating brood parasitism and co‐evolutionary dynamics in avian systems. Through an extensive synthesis of published literature, verified media reports, and meticulously curated visual documentation contributed by citizen scientists, we present a comprehensive update on cuckoo–host diversity and their intricate ecological relationships across China. Our study identifies 17 cuckoo species, with 15 confirmed as brood parasites exploiting an extensive network of 142 host species spanning 74 genera and 34 families within the passerine assemblage. While we observed broad overlaps in the ranges of host body mass and egg volume across different cuckoo species, phylogenetic generalized linear mixed models reveal significant patterns of adaptive matching in both body mass and egg volume parameters between cuckoos and their respective hosts. Our findings demonstrate striking specificity in host selection, with minimal overlap in actual host species utilization among sympatric cuckoos, suggesting sophisticated niche partitioning strategies to mitigate interspecific competition. Nevertheless, critical knowledge gaps persist, particularly regarding the evolutionary dynamics of egg phenotype mimicry in relation to specific host species. Finally, we introduce a real‐time tracking program designed to engage citizen scientists in ongoing documentation of parasitism events, facilitating dynamic updates to host–parasite records.

## Graphical abstract

China is a global hotspot for cuckoo‐host diversity, with 17 cuckoo species, exploiting 142 host species. We reveal adaptive matching in body size and egg traits, along with niche partitioning among cuckoos, while identifying eight new host species. A real‐time tracking program was designed to engage citizen scientists in ongoing documentation of parasitism events, facilitating dynamic updates to host–parasite records.

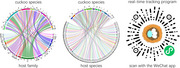

## 1 Introduction

Avian brood parasitism is a reproductive strategy in which a parasitic bird species does not build its own nest but lays its eggs in the nests of host species, transferring the cost of brooding and raising offspring to the hosts (Davies 2000). Brood parasitism may occur within the same species, known as intraspecific brood parasitism, or across different species, referred to as interspecific brood parasitism, with parasites being either facultative or obligate. Unlike facultative parasites, obligate parasitic species are entirely dependent on host birds for reproduction (Mann 2017; Soler 2017).

Cuckoos are the largest group of parasitic birds in the world, with nearly 60 obligate brood parasitic species in the Cuculidae family, which contains 144 species (Payne 2005; Hughes 2013). Cuckoos and their host species have undergone extensive coevolution over a long period (Feeney et al. 2014). The dynamic interaction between cuckoos and their hosts can spark coevolutionary arms races, driving host species to develop defensive strategies against parasitism, which in turn prompts parasites to evolve counteradaptations, leading to further host countermeasures, and perpetuating an ongoing cycle of adaptation and counteradaptation (Dawkins and Krebs 1979; Rothstein 1990; Soler 2014). This interaction, which has captivated researchers for a long time, promotes exploration of the causes, processes, and outcomes of coevolution, holding significant importance in coevolutionary studies (Servedio and Lande 2003; Antonov et al. 2006; Yang et al. 2010b; Feeney et al. 2012).

Investigating the parasitism patterns of cuckoos across different host species is critical for understanding the coevolution between cuckoos and their hosts (Feeney et al. 2014). This involves identifying which hosts are exploited by cuckoos, the frequency with which certain hosts are parasitized, and the degree of matching between cuckoo eggs and host eggs in terms of size, coloration, and spot patterns (Krüger and Davies 2002; Servedio and Lande 2003; Kilner 2006). These findings enhance our understanding of the interactions between cuckoos and their hosts, including whether there is overlap and competition among cuckoos in host exploitation (Brooker and Brooker 1990; Møller et al. 2011; Langmore et al. 2024).

Furthermore, by examining the matching of cuckoo eggs to host eggs, insights can be gained into the parasitic history of cuckoos with respect to different hosts (Krüger 2006; Yang et al. 2015). For hosts that construct open nests, a high morphological similarity between cuckoo eggs and host eggs suggests a long history of parasitism by the cuckoo on that host (Servedio and Lande 2003; Kilner 2006; Yang et al. 2010b). Conversely, if cuckoo eggs are highly dissimilar to host eggs, it is likely that the host is a newly exploited target for the cuckoo (Takasu et al. 2009; Yang et al. 2012a). For example, the Chinese babax (*Babax lanceolatus*) produces deep blue eggs, whereas the white‐browed laughingthrush (*Pterorhinus sannio*) lays pure white and pale blue eggs. The large hawk‐cuckoo (*Hierococcyx sparverioides*), which parasitizes both Chinese babax and white‐browed laughingthrush, deposits eggs that closely resemble both pure white and pale blue egg morph of the laughingthrush under avian visual perception. These findings align with the prediction that the white‐browed laughingthrush serves as the original and primary host, having engaged in a more prolonged coevolutionary relationship with the large hawk‐cuckoo compared to the Chinese babax, which represents a more recent host that the large hawk‐cuckoo has adopted through a host shift (Yang et al. 2015).

These fundamental data also provide crucial insights for investigating the coevolutionary process between cuckoos and their hosts under multiple cuckoo species in China (Liang et al. 2017a). China harbors a rich diversity of cuckoos, with a total of 17 species across 7 genera recorded, making it an ideal region for studying the cuckoos and their hosts (Yang et al. 2012c; Zheng 2023). Research on cuckoos in China began relatively late and some cuckoos species within this group have been largely overlooked, remaining far more obscure compared to the well‐studied Common Cuckoo (Liang et al. 2017a). Even so, ongoing studies are continuously investigating host diversity, host egg recognition abilities, host anti‐parasitism strategies, and the coevolutionary dynamics of cuckoo–host interactions in China (e.g., Wang et al. 2023; Yang and Zhang 2024; Zhao et al. 2024).

To minimize detection by returning hosts, cuckoos complete egg deposition in host nests rapidly and covertly, rendering this parasitic behavior particularly challenging to observe. Therefore, understanding the diversity of cuckoo's hosts is a process of long‐term accumulation. Over a decade ago, Yang et al. (2012c) provided a comprehensive overview of cuckoo and host diversity in China, documenting a total of 55 host species belonging to 15 families parasitized by 11 species of cuckoos. Since then, numerous studies on Chinese cuckoos and their hosts have been published, with continuous updates on records of cuckoo parasitism and an increasing array of host species. Concurrently, with the rise in the number of birdwatchers and bird photographers in China, the likelihood of discovering avian brood parasitism has increased. However, these reports and records of parasitism are relatively scattered, and some records have not been formally published. The present study collects and analyzes published literature records, media reports, as well as images and video footage provided by volunteers, to compile a comprehensive account of cuckoos' host diversity and the relationship between cuckoos and their hosts in China. It aims to provide updated information, differentiating between the known and unknown, and to serve as a reference for further research on the interactions between cuckoos and their hosts. To address critical data gaps in parasitism records, we further developed a real‐time tracking program designed to facilitate ongoing documentation and public participation in cuckoo research.

## 2 Data Collection and Analyses

We collected records of brood parasitism by cuckoos through published literature, videos, and photographs taken by bird photographers, and media reports. First, we conducted a comprehensive search on the Web of Science and the Chinese literature database “CNKI” for published articles or reports without language restrictions ranging in date from April 1, 2012, to April 20, 2025. Articles documenting avian brood parasitism in China were included by using search terms: the English and scientific names of the 17 parasitic cuckoo species confirmed in China (Table 1) as keywords, with “and” or “or” as conjunctions, supplemented by “brood parasitism,” “host,” “parasitic eggs,” and “parasitic chicks” as additional search terms.

**TABLE 1:** Body mass and egg volume of host species parasitized by different cuckoo species.

Cuckoo species		Body mass of host species (g)			Egg volume of host species (mm^3^)		
English name	Latin name	Mean	CV	Range (*n*)	Mean	CV	Range (*n*)
Asian koel	*Eudynamys scolopaceus*	204.9	87.6%	51.5–725.0 (12)	9.7	44.2%	5.6–19.0 (11)
Chestnut‐winged cuckoo	*Clamator coromandus*	83.8	56.2%	32.0–183.5 (11)	6.3	42.5%	3.0–11.6 (11)
Large hawk‐cuckoo	*Hierococcyx sparverioides*	60.5	92.4%	12.2–230.8 (15)	5.3	68.1%	1.6–15.3 (13)
Indian cuckoo	*Cuculus micropterus*	54.1	51.0%	15.0–96.5 (10)	4.7	31.6%	3.1–6.8 (14)
Square‐tailed drongo‐cuckoo	*Surniculus lugubris*	35.0	86.5%	9.5–87.5 (6)	3.4	68.5%	1.3–7.0 (6)
Northern hawk‐cuckoo	*Hierococcyx hyperythrus*	27.2	74.5%	11.9–69.8 (7)	2.8	43.7%	1.5–4.8 (7)
Common cuckoo	*Cuculus canorus*	27.0	96.7%	6.9–149.5 (45)	2.7	60.2%	0.8–8.9 (44)
Hodgson's hawk‐cuckoo	*Hierococcyx nisicolor*	19.6	49.4%	10.5–34.3 (13)	2.5	33.6%	1.6–4.1 (12)
Himalayan cuckoo	*Cuculus saturatus*	14.6	66.3%	5.0–32.0 (9)	2.1	57.2%	1.1–4.2 (9)
Oriental cuckoo	*Cuculus optatus*	13.2	42.9%	5.4–26.0 (27)	1.8	44.3%	0.9–3.8 (27)
Lesser cuckoo	*Cuculus poliocephalus*	10.8	52.5%	6.6–25.0 (9)	1.8	54.9%	1.1–4.2 (9)
Asian emerald cuckoo	*Chrysococcyx maculatus*	10.9	84.1%	5.0–32.5 (8)	2.0	72.4%	0.9–4.5 (8)
Plaintive cuckoo	*Cacomantis merulinus*	8.1	20.7%	6.4–12.0 (11)	1.1	20.6%	1.0–1.7 (10)
Banded bay cuckoo	*Cacomantis sonneratii*	14.3	NA	14.3 (1)	1.8	NA	1.8 (1)
Violet cuckoo	*Chrysococcyx xanthorhynchus*	12.2	NA	12.2 (1)	1.6	NA	1.6 (1)
Pied cuckoo	*Clamator jacobinus*	NA	NA	NA	NA	NA	NA
Common hawk‐cuckoo	*Hierococcyx varius*	NA	NA	NA	NA	NA	NA

*Note*: The “*n*” in parentheses represents the sample size.

Abbreviation: CV = coefficient of variation.

Second, the collection of photographs and videos involved gathering materials provided by local bird researchers and volunteers, as well as searching for brood parasitism records on platforms such as the Chinese bird photography website “Birdnet (www.birdnet.cn)” and social network platforms in China, including “WeChat Public Accounts (https://mp.weixin.qq.com),” “Weibo (https://www.weibo.com/),” “Video Channels (https://channels.weixin.qq.com),” and “Tik Tok (www.douyin.com),” using the Chinese names of the 17 parasitic cuckoo species confirmed in China (Table 1) combined with Chinese term “鸟类巢寄生(brood parasitism)” or “宿主 (host)” as keywords. We identified valid records based on photographs or videos showing adult hosts feeding cuckoo nestlings or fledglings (species identifiable) within China.

Third, for the collection of media reports, the Chinese search engine “Baidu” was used with the Chinese, English, and scientific names of the 17 parasitic cuckoo species confirmed in China (Table 1) as keywords, again with “and” or “or” as conjunctions, supplemented by Chinese term “鸟类巢寄生 (brood parasitism),” “宿主 (host),” “寄生卵 (parasitic eggs),” and “寄生的雏鸟 (parasitic chicks).” Reports of brood parasitism events occurring within China, supported by photographs or videos, were considered valid records.

For observational records sourced from media reports and social media platforms, we systematically extracted temporal and spatial data regarding brood parasitism events based on published content. When necessary, we contacted original publishers to verify and supplement this information. Only records with clearly documented occurrence times and locations were included in our analysis. For the identification of cuckoo nestlings and fledglings, we referenced the photographic documentation and morphological descriptions provided by Yang et al. (2012b, 2012c). Data on body weight, egg volume (calculated based on egg dimensions, Hoyt 1979), egg weight, and egg phenotypes for different cuckoo and host species were derived from the corresponding references (see Supporting Information S1), as well as from the book *Ornis of China (Volume 2 Passeriformes)* (Zhao 2001) and *Birds of the World* (https://birdsoftheworld.org). For the differentiation and delineation of various egg phenotypes, as well as the determination of whether cuckoo eggs exhibit polymorphism or monomorphism, we referred to the methodology outlined by Yang et al. (2020).

The phylogenetic trees of hosts were derived from the global bird phylogeny (http://birdtree.org) using the option “Hackett All Species: a set of 10 000 trees with 9993 OTUs each” (Jetz et al. 2012). From these data, we generated 5000 pseudo‐posterior distributions and built a Maximum Clade Credibility tree with mean node heights using TreeAnnotator v1.8.2 of the BEAST package (Drummond and Rambaut 2007; Ricklefs and Jønsson 2014). This final phylogenetic tree (Supporting Information S2) served as the basis for subsequent phylogenetic analyses. We used the phylogenetic generalized linear mixed model (PGLMM) to test for associations between the body mass of cuckoos and their hosts, as well as between the egg volume of cuckoos and their hosts following the method of Ives and Helmus (2011) and Willis et al. (2014). The MCMCglmm (generalized linear mixed models using Markov chain Monte Carlo techniques) was employed (Hadfield 2010). We accounted for phylogenetic dependence among host species by specifying the egg volume and body mass of hosts as the predictor variables and the egg volume and body mass of cuckoos as the response variable. The model was run with two parallel Markov chains, each iterated 10 000 times. We discarded the initial 1000 iterations as burn‐in and applied a thinning interval of 20. Convergence was verified using the Gelman–Rubin statistic, with acceptable diagnostic values below 1.1 (Gelman and Rubin 1992). A host species effect on host exploitation by cuckoos was deemed statistically significant if the 95% Bayesian credible intervals for parameter estimates excluded zero (Kéry and Royle 2016). Additionally, we employed the phylogenetic diversity (PD) measurements for each cuckoo's host assemblage. The PD was measured as the sum of branch lengths linking the sampled individuals within a given tree (Faith 1992), consistently incorporating the path to the root—including the basal branches uniting all taxa (Rodrigues and Gaston 2002). The PD values in this study are expressed in millions of years (Myr), consistent with the branch length measurements from the original phylogeny (Jetz et al. 2012). The PGLMMs analyses, PD calculation, data distribution density analyses, and data visualization were performed using the *MCMCglmm*, *phytools*, *vegan*, *coda*, *picante*, *tidyverse*, *ape*, and *ggplot2* packages in R (v.4.4.2) for Windows (Plummer et al. 2006; Hadfield 2010; Kembel et al. 2010; R Core Team 2024).

## 3 The Number of Host Species of Cuckoos in China

**FIGURE 1:**
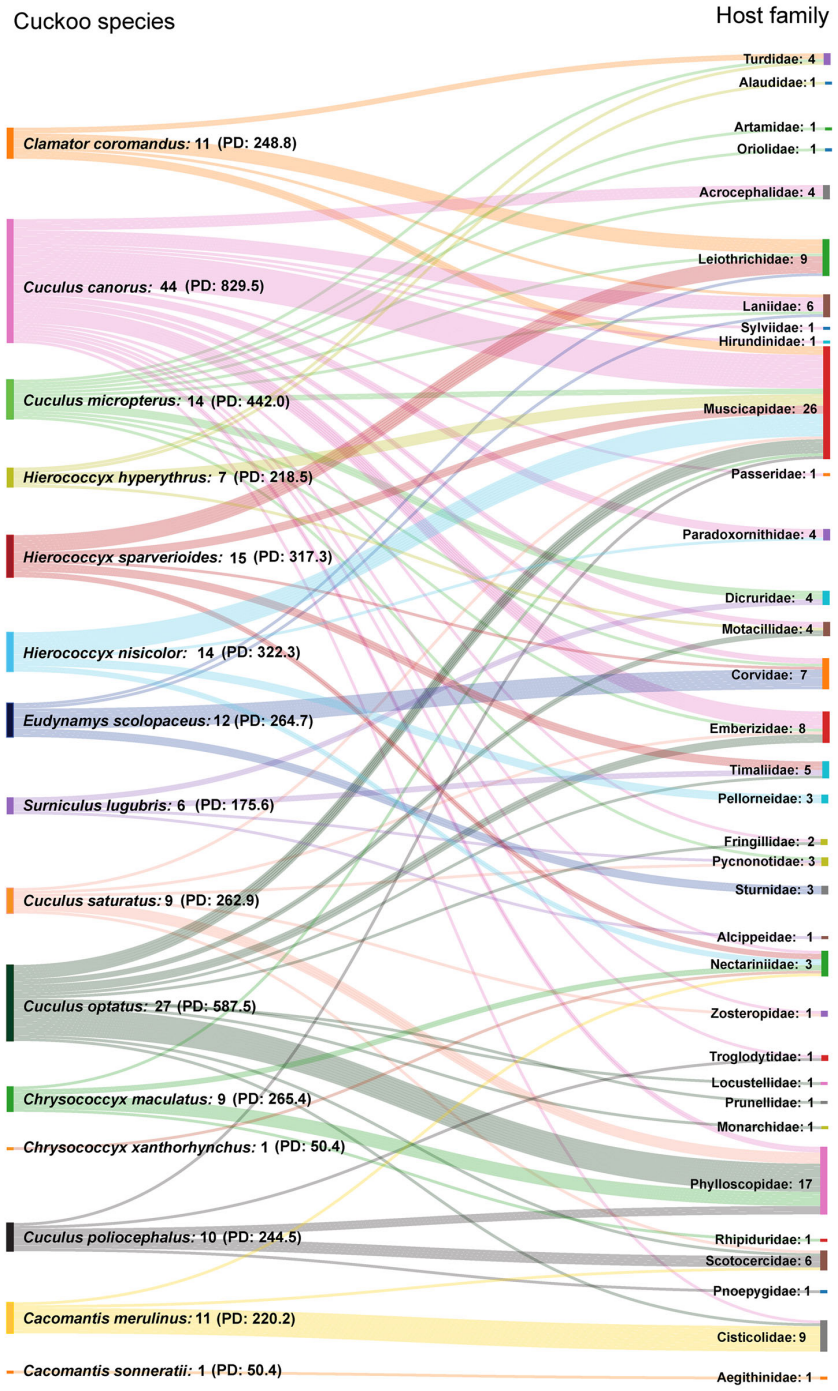
The number of actual and potential cuckoo hosts in China. The numbers following cuckoo species names indicate the number of host species they parasitize and the numbers following family names denote the number of host species included within that family. PD: the value of phylogenetic diversity of the host species targeted by each cuckoo species.

Through a comprehensive review of literature, media reports, and images and video footage provided by volunteers, a total of 191 nest parasitism records related to cuckoos in China and its neighboring regions were identified. These records encompass 15 cuckoo species across 7 genera within the Cuculidae family. We classify the cuckoos’ hosts actually recorded in China as actual hosts, while those recorded in neighboring regions of China that also have breeding distributions within China are classified as potential hosts. The actual and potential host species of these cuckoos in China comprise 142 species of passerine birds, belonging to 74 genera and 34 families. The total PD across all host species was 2162.6. Among these, 95 species of actual hosts (PD = 1541.2) were recorded, distributed across 54 genera and 27 families (Figure 1; Supporting Information S1). In the following analysis, we combined both actual and potential hosts for statistical analysis.

Among the cuckoo species documented in China, the common cuckoo exploits the largest number of hosts, parasitizing 44 species (31% of the total 142 recorded host species), belonging to 25 genera and 16 families. Correspondingly, its host assemblage also displays the highest PD (PD = 829.5). In contrast, the banded bay cuckoo (*Cacomantis sonneratii*) and the violet cuckoo (*Chrysococcyx xanthorhynchus*) exhibit the most restricted host specificity, each reported to parasitize only a single host species. Consequently, these two cuckoo species share the lowest observed PD value (50.4; Figure 1).

Among the host species, those belonging to the Muscicapidae family constitute the largest proportion, with 26 species. For the host species, the little spiderhunter (*Arachnothera longirostra*) is parasitized by the highest (five) number of cuckoo species (Figure 1; Supporting Information S1).

## 4 The Relationship Between Cuckoos and Their Hosts in Terms of Body Mass and Egg Volume

The Asian koel (*Eudynamys scolopaceus*) exploits hosts with the largest average body mass and egg volume, as well as the broadest range of host body mass and egg volume, with an average host body weight of 204.9 g (CV: 87.6%, range: 51.5–725.0) and an average egg volume of 9.7 mm^3^ (CV: 44.2%, range: 5.6–19.0). In contrast, the plaintive cuckoo (*Cacomantis merulinus*) exploits hosts with the smallest average body mass and egg volume, as well as the narrowest range of host body mass and egg volume, with an average host body weight of 8.1 g (CV: 20.7%, range: 6.4–12.0) and average egg volume of 1.1 mm^3^ (CV: 20.6%, range: 1.0–1.7; Table 1).

There is considerable overlap in the range of host body mass and egg volume utilized by cuckoos. Approximately 66.7% (10/15) of cuckoo species exhibit a preference for using hosts whose average body mass is < 30 g and average egg volume is < 3 mm^3^. Conversely, 33.3% (5/15) of cuckoo species tend to exploit hosts whose average body mass is ≥30 g and average egg volume is ≥3 mm^3^ (Table 1).

Although the host groups parasitized by cuckoos exhibit overlap in body mass and egg volume, cuckoos preferentially parasitize host species that match their own body mass and egg volume. In the PGLMMs analysis that controlled for phylogeny, a significant positive correlation was found between the body mass of cuckoos and their hosts (95% CI: 0.26–0.68), as well as between the egg volume of cuckoos and their hosts (95% CI: 0.34–0.52) indicating that cuckoos exhibit matching adaptations in body mass and egg volume relative to their hosts.

**FIGURE 2:**
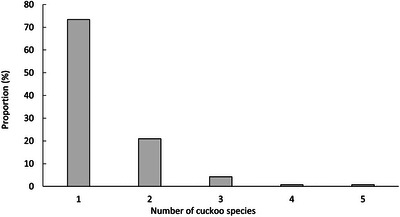
Relative proportion of host species parasitized by a specific number of cuckoos.

Furthermore, there is minimal overlap in the host species exploited by different cuckoo species, with most host species (73.4%, 105/143) being parasitized by a single cuckoo species (Figure 2). This phenomenon may be attributed to two primary factors. First, it likely reflects a strategy to avoid interspecific competition (Baker 1942). By selecting different host species and varying the timing of parasitism, sympatric cuckoos can reduce competition for host resource utilization (Brooker and Brooker 1989; Higuchi 1989; Møller et al. 2011). Second, it may result from shifts in suitable hosts. Over time, prolonged parasitic pressure drives hosts to evolve defensive mechanisms against brood parasitism, such as increased aggression toward parasitic cuckoos and enhanced egg recognition capabilities, thereby reducing the likelihood of successful parasitism (Feeney et al. 2014). As a consequence, these hosts become less suitable for parasitism, prompting cuckoos to seek new, more suitable host species (Moksnes et al. 2013; Feeney et al. 2014).

## 5 The Level of Egg Polymorphism in Cuckoos

**FIGURE 3:**
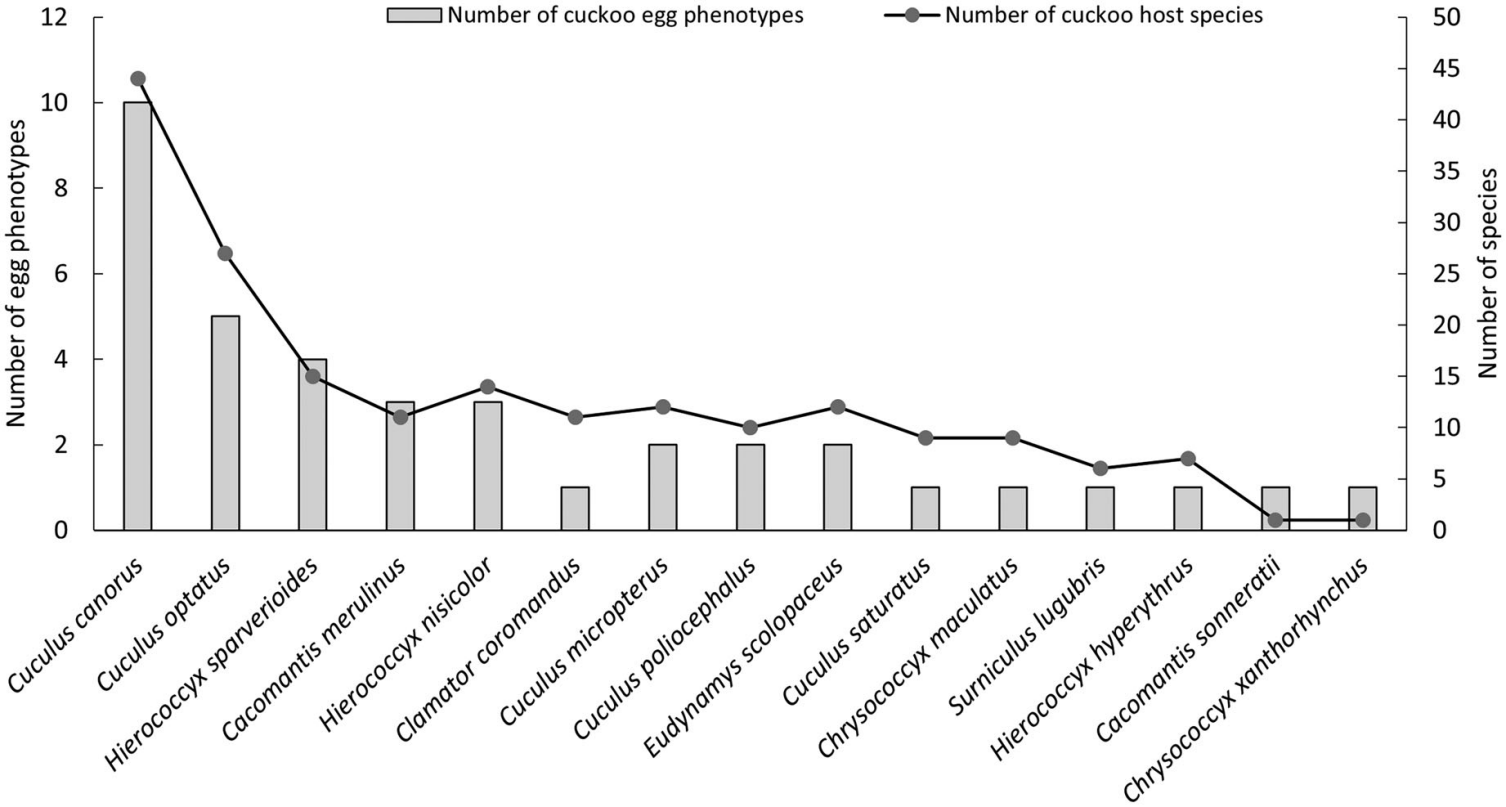
Number of cuckoo hosts and egg phenotypes in China.

The level of egg polymorphism in cuckoos reflects the number of host species they exploit, with a higher level of egg polymorphism corresponding to a greater number of host species, a significant correlation was observed between these two variables (*r* = 0.863, *p* = 0.001, Spearman correlation). In this study, the common cuckoo has the highest number of host species and also exhibits the greatest egg polymorphism, with a total of 10 distinct egg phenotypes. This is followed by the oriental cuckoo and the large hawk‐cuckoo, each displaying five and four egg phenotypes, respectively (Figure 3). In contrast, the chestnut‐winged cuckoo (*Clamator coromandus*), Himalayan cuckoo (*Cuculus saturatus*), northern hawk‐cuckoo (*Hierococcyx hyperythrus*), Asian emerald cuckoo (*Chrysococcyx maculatus*), square‐tailed drongo‐cuckoo (*Surniculus lugubris*), banded bay cuckoo, and violet cuckoo show the least egg polymorphism, each with only a single egg phenotype. However, most of the parasitic cuckoos might simply be understudied and therefore we do not know if they have multiple egg phenotypes or not.

The egg polymorphism arises from coevolutionary arms races between cuckoos and their hosts. This phenomenon is driven by reciprocal adaptations and counteradaptations: hosts evolve egg polymorphism as a defense mechanism to detect and reject parasitic eggs, while cuckoos evolve mimetic egg phenotypes to evade detection (Kilner 2006; Yang et al. 2017, 2020). For instance, ashy‐throated parrotbills (*Sinosuthora alphonsiana*) exhibit polymorphic egg phenotypes, and common cuckoos parasitizing this host species have evolved egg phenotypes that precisely match those of their hosts (Yang et al. 2010b). An analogous pattern is observed in plaintive cuckoos and their common tailorbird (*Orthotomus sutorius*) hosts, both of which display polymorphic eggs with corresponding phenotypes (Yang et al. 2016). A comparable scenario has been documented in common cuckoos and their *Fringilla* finch hosts (Vikan et al. 2011). Additionally, as we have mentioned, large hawk‐cuckoos, which parasitize white‐browed laughingthrushes, have developed polymorphic eggs—either pure white or pale blue—that mimic the egg phenotypes of their laughingthrush hosts (Yang et al. 2015). While the co‐occurrence of egg polymorphism in both brood parasites and their hosts is relatively rare, cuckoo eggs can also exhibit polymorphism as a result of frequency‐dependent selection pressures (Yang et al. 2017).

## 6 The Egg Phenotypes Imitation of Cuckoos

**FIGURE 4:**
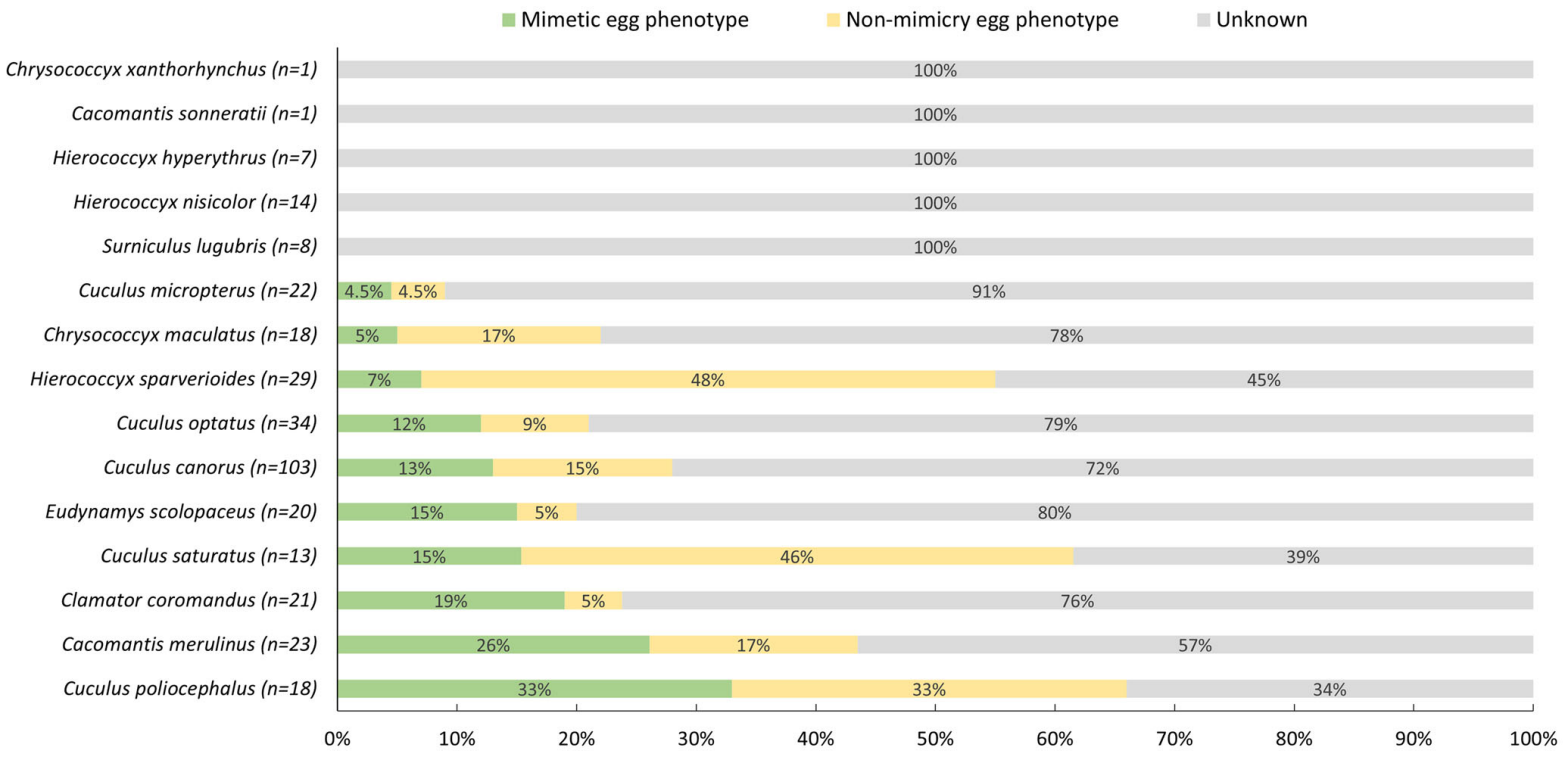
The proportion of cuckoo egg phenotypes mimicking host eggs. The *n* value represents the number of records of cuckoo parasitism on different hosts.

Although most cuckoos have evolved polymorphic eggs, not all egg morphs match those of their host species. For instance, the common cuckoo exhibits ten distinct egg morphs. Among the parasitism records of common cuckoo, 13% of cases demonstrated egg morphs mimicry of host eggs, 15% showed no mimicry, and 72% remained uncertain as to whether mimicry occurred (Figure 4, Supporting Information S1). In China, the common cuckoo has been documented parasitizing 44 host species, with over half of these hosts having very few parasitism records (no more than two records). It causes the knowledge gap on egg polymorphism of cuckoos. On the other hand, when some hosts are unsuitable for parasitism, cuckoos may explore new hosts (Moksnes et al. 2013; Feeney et al. 2014). Given that egg morph evolution is a prolonged process (Kilner 2006), cuckoo egg morphs may not immediately match those of newly targeted hosts.

Additionally, while the lesser cuckoo (*Cuculus poliocephalus*), plaintive cuckoo (*Cacomantis merulinus*), and chestnut‐winged cuckoo (*Clamator coromandus*) exhibit only two, three, and one egg morphs respectively, their parasitism records show mimicry rates of 33%, 26%, and 19%, respectively, which are higher than that of the common cuckoo (Figure 4). This may be attributed to their relatively limited host exploitation—10, 11, and 11 host species, respectively—driving them to enhance egg mimicry to increase parasitism success. In our findings, the mimicry status of egg morphs among most cuckoo species in relation to their hosts remains unclear, primarily due to the lack of records and observations of their parasitic eggs, especially for rare cuckoo species in China, such as the Northern hawk‐cuckoo (*Hierococcyx hyperythrus*), banded bay cuckoo, and violet cuckoo.

## 7 Host Information by Cuckoo Species

### 7.1 Common Cuckoo *Cuculus canorus*

The common cuckoo is a widely distributed avian species, with its breeding range extending across the Eurasian continent (Payne et al. 2020a). As one of the earliest studied obligate brood parasitic birds, the common cuckoo parasitizes 184 host species globally (Moksnes et al. 2013; Yang et al. 2020). In China, the common cuckoo is distributed across all provinces and is one of the most prevalent cuckoo species (Yang et al. 2012c; Zheng 2023). It also parasitizes the highest number of host species in the country (Figure 1).

The common cuckoo exhibits the most extensive egg polymorphism with 10 distinct egg phenotypes currently documented (Figure 3); three of them match the egg phenotypes of the ashy‐throated parrotbill, including (1) turquoise, (2) pale blue, and (3) pure white (Yang et al. 2010b). Furthermore, three additional egg phenotypes—(4) light cyan with brown spots, (5) off‐white with brown spots, and (6) off‐white with olive spots—also demonstrate mimicry of host egg phenotypes (Zhang 1989; Tian et al. 1991; Yang et al. 2012c; Liu et al. 2022). However, four other egg phenotypes—(7) turquoise with violet spots, (8) white with gray spots, (9) light taupe with dark brown spots, and (10) pinkish white with sandy brown spots—do not mimic the egg phenotypes of their respective host species (Supporting Information S1). Some host species parasitized by the common cuckoo have been recorded during the nestling or fledgling stages (Yang et al. 2012c). The cuckoo's egg phenotypes associated with these hosts remain undocumented and warrant further research.

### 7.2 Oriental Cuckoo *Cuculus optatus*

The oriental cuckoo has a breeding range that spans northern Eurasia, encompassing regions such as Kazakhstan, Russia, China, South Korea, and Japan. In China, it is distributed across the northern and southeastern parts of the country (Payne and Kirwan 2020d). Within China, this cuckoo species parasitizes nine host species, which belong to seven genera and five families. Additionally, 18 host species parasitized by the oriental cuckoo have been recorded in neighboring countries of China (Higuchi 1998; Meshcheryagina et al. 2017, 2018; Kim 2011; Payne and Kirwan 2020d). Notably, all 18 of these host species also breed in China.

To date, five egg phenotypes of the oriental cuckoo have been documented within China. In Taiwan province, the oriental cuckoo parasitizes the yellow‐bellied prinia (*Prinia flaviventris*), and three egg phenotypes have been observed in this context. Two of these phenotypes—(1) pink with dense red spots and (2) unspotted chocolate—match the egg phenotypes of the host, while the third phenotype, (3) white with brown spots, does not (La Touche 1927; Zhang 1980; Wang 2015). Furthermore, the phenotype (4) turquoise with red spots (parasitizing the lesser shortwing, *Brachypteryx leucophrys*) and (5) pure white (parasitizing the Hume's warbler, *Phylloscopus humei*, and the pale‐legged leaf warbler, *Phylloscopus tenellipes*) both closely match the egg phenotypes of their respective hosts (La Touche 1927; Wang et al. 2014, 2004).

### 7.3 Hodgson's Hawk‐Cuckoo *Hierococcyx nisicolor*

The Hodgson's hawk‐cuckoo has a breeding range that extends from Nepal through the eastern Himalayas and southern Assam in India to eastern Myanmar, southern and eastern China, Thailand, and northern and central Laos (del Hoyo et al. 2020a). In China, this cuckoo species has been recorded parasitizing four host species belonging to the Muscicapidae family (Su et al. 2016; Luo et al. 2018). Additionally, while 10 host species of the Hodgson**’**s hawk‐cuckoo have been documented outside of China— 7 in Myanmar and 3 in India (del Hoyo et al. 2020a)—all 10 of these host species also breed within China, making them potential hosts for the Hodgson's hawk‐cuckoo in the country (Figure 1).

To date, three egg phenotypes of Hodgson**’**s hawk‐cuckoo have been documented: (1) unspotted chocolate, (2) plain olive‐brown becoming darker at the larger end, and (3) olive‐brown becoming green at the larger end. The first phenotype has been recorded in China, while the latter two were documented in India. However, the egg phenotypes associated with most of the Hodgson**’**s hawk‐cuckoo**’**s host species remain poorly understood (del Hoyo et al. 2020a).

### 7.4 Chestnut‐Winged Cuckoo *Clamator coromandus*

The chestnut‐winged cuckoo has a breeding range that includes northern India, eastern Nepal, eastern and southern China, and the Thai‐Malay Peninsula (Payne and Kirwan 2020a). In China, this cuckoo species has been recorded parasitizing five host species (Yang et al. 2012c; Huo et al. 2014). Additionally, six host species that also breed within China have been documented outside of China (Erritzøe et al. 2012; Figure 1).

The chestnut‐winged cuckoo employs a unique parasitic strategy, often laying two or even multiple eggs in a single host nest. Unlike some cuckoo species, the nestling of the chestnut‐winged cuckoo does not exhibit obligate eviction behavior and can coexist with host chicks. However, they gain a competitive advantage through superior weight and begging behavior, which enhances their survival (Soler 1990). The egg phenotype of the chestnut‐winged cuckoo is relatively uniform, with only one phenotype currently documented: the turquoise. This phenotype closely resemble the light turquoise eggs of the Hwamei and Chinese babax (Huo et al. 2014). For the masked laughingthrush (*Pterorhinus perspicillatus*), which lays eggs of two phenotypes—unspotted light cyan and light green with auburn spots, partial egg phenotype mimicry is observed (La Touche 1927).

### 7.5 Large Hawk‐Cuckoo *Hierococcyx sparverioides*

The large hawk‐cuckoo has a breeding range that encompasses the Himalayas, extending from northern Pakistan and India through Nepal, the Naga Hills, Manipur, and Meghalaya, eastward to China (south of the Yellow River valley), and southward to Myanmar, Thailand, and Indochina (Payne and Kirwan 2020c). In China, this cuckoo species has been recorded parasitizing 11 host species, which belong to nine genera and four families (Yang et al. 2012c; Hu et al. 2013). Additionally, four host species that also breed within China have been documented as being parasitized in Indochina (Clement and Christie 2020; Payne and Kirwan 2020c).

To date, four egg phenotypes of the large hawk‐cuckoo have been documented: (1) pure white, (2) off‐white with brown spots, (3) turquoise, and (4) olive gray with brown spots. Only the pure white morph matches one of the two egg phenotypes (pure white and pure blue) of the white‐browed laughingthrush (Yang et al. 2012c).

### 7.6 Lesser Cuckoo *Cuculus poliocephalus*

The Lesser cuckoo has a breeding range that extends from northern Pakistan and Kashmir eastward through the Himalayan foothills to the Khasi and Naga Hills, northern Myanmar, and northern Indochina, and across China to Ussuriland, Korea, and Japan (Payne et al. 2020b). In China, this cuckoo species has been recorded parasitizing seven host species (Yang et al. 2012c; Wang et al. 2016; Yang et al. 2023). Additionally, three host species have been documented as being parasitized in India, but all three of these host species also breed within China (Ali and Ripley 1973; Becking 1981; Marchetti 1992).

In China, two egg phenotypes of the Lesser Cuckoo have been documented. The egg phenotype (1) unspotted chocolate was found in the nests of the brownish‐flanked bush warbler *Horornis ffortipes*, which lays pure chocolate eggs (Yang et al. 2012c), and (2) unspotted light cyan, found in the nests of the blue‐and‐white flycatcher, which lays pure white eggs (Zhao and He 1981; Zhao 1985). Furthermore, in India, an unmarked white egg of the lesser cuckoo has been recorded, which closely resembles the egg phenotype of its host, the large crowned warbler *Phylloscopus occipitalis* (Payne et al. 2020b).

### 7.7 Himalayan Cuckoo *Cuculus saturatus*

The breeding range of the Himalayan Cuckoo extends from the Himalayas to eastern and southern China (Payne and Kirwan 2020b). The cuckoo has been recorded with a total of nine host species in China, belonging to six genera and six families (Figure 1; Supporting Information S1). Currently, only one egg phenotype of this cuckoo has been documented, which is white with a few brown spots. This egg phenotype is found in the nests of hosts that lay different egg phenotypes, such as Blyth's leaf warbler (*Phylloscopus reguloides*) with pure white eggs, brownish‐flanked bush warbler (*Cettia fortipes*) with unspotted chocolate eggs, yellow‐throated bunting (*Emberiza elegans*) (off‐white with black spots eggs), and collared finchbill (*Spizixos semitorques*) (pink with violet spots eggs) (Yang et al. 2011, 2012c; Su et al. 2014).

### 7.8 Plaintive Cuckoo *Cacomantis merulinus*

The breeding range of the plaintive cuckoo extends from eastern India to Myanmar, southern China, Indochina, and as far south as Malaysia and Indonesia (Payne and Kirwan 2020e). This cuckoo has been recorded with a total of five host species in China (Yang et al. 2012c; Huang et al. 2015; Liang et al. 2017b). In addition, there are six host species with parasitic records from the Indian subcontinent (Well 1999; Payne and Kirwan 2020e), which also breed in China and are potential hosts for the plaintive cuckoo in China (Figure 1). The cuckoo currently has three documented egg phenotypes. The egg phenotype (1) pale green with light rufous spots and (2) white with brown spots correspond to the two egg phenotypes of the host, the common tailorbird (Yang et al. 2012c). The egg phenotype (3) blue with brown spots matches only a subset of egg phenotypes for the rufescent prinia (Liang et al. 2017b).

### 7.9 Indian Cuckoo *Cuculus micropterus*

The breeding range of the Indian cuckoo extends from India and Nepal eastward to eastern China, northward to northeastern China and Russia, and southward to Indochina and the Malay Peninsula (Payne 2020d). In China, the Indian cuckoo has been documented to parasitize a total of 12 host species, which belong to 12 genera and 11 families (Figure 1). Additionally, two other host species that also breed in China have been recorded in Indochina (Payne 2020d). To date, only two egg phenotypes of the Indian cuckoo have been documented (Yang et al. 2012c): (1) pinkish white with rufous spots, which is associated with parasitism of the black drongo (*Dicrurus macrocercus*), and (2) off‐white with rufous spots, which is associated with parasitism of the Chinese blackbird (*Turdus mandarinus*).

### 7.10 Asian Koel *Eudynamys scolopacea*

The breeding range of the Asian koel encompasses the Indian Subcontinent and mainland Southeast Asia, extending eastward to southern and eastern China, and southward to the Philippines and the Sundaic Islands (Limparungpatthanakij 2020). In China, the Asian koel has been documented to parasitize a total of eight host species, which belong to seven genera and three families, with the majority being members of the Corvidae family (Yang et al. 2012c; Lin et al. 2024). Additionally, four other host species that also breed in China have been recorded as hosts in Bangladesh and Thailand (Begum et al. 2011; Jadav and Parasharya 2014; Limparungpatthanakij 2020).

In China, only two egg phenotypes of the Asian koel have been documented: (1) sage green, associated with parasitism of the black‐collared starling (*Gracupica nigricollis*), and (2) turquoise with numerous brownish spots, associated with parasitism of the house crow (*Corvus splendens*). Both egg phenotypes closely resemble the egg phenotypes of their respective hosts (Yang et al. 2012c). Outside of China, the Asian koel exhibits egg polymorphism. Specifically, when parasitizing crow nests, the eggs display a range of ground colors, including gray‐blue, pale green, and yellowish hues, often accompanied by reddish‐brown and black spotting. Additionally, eggs with a pale reddish‐salmon coloration have been observed in the nests of various other host species (Erritzøe et al. 2012).

### 7.11 Asian Emerald Cuckoo *Chrysococcyx maculatus*

The breeding range of the Asian emerald cuckoo spans the Himalayas, extending from approximately Garhwal eastward through Nepal, Bhutan, northeastern India, Bangladesh, and southern China, and southward to Myanmar, northwestern Thailand, northern Laos, and northern and central Vietnam (Payne 2020a). In China, this cuckoo has been documented to parasitize a total of seven host species, which belong to three genera and three families (Figure 1). Due to its relatively small body size, the Asian emerald cuckoo primarily targets smaller host species, particularly those within the *Phylloscopus* genus, such as Bianchi's warbler (*Phylloscopus valentini*) (Yang et al. 2012c; Zhang et al. 2021; Lin et al. 2024). Additionally, two other host species that also breed in China have been recorded as hosts in India (Payne 2020a). To date, only one egg phenotype has been documented for the Asian emerald cuckoo: white with brown spots. However, the egg phenotypes of its primary *Phylloscopus* hosts are typically pure white, which does not match the cuckoo's egg phenotype (Yang et al. 2012c; Lin et al. 2024). This lack of egg phenotype mimicry may be attributed to the semi‐open nest structure of *Phylloscopus* species, where low light conditions inside the nest reduce the reliance on egg color or spotting patterns as cues for egg recognition by the host. Consequently, the Asian emerald cuckoo's parasitic eggs do not exhibit phenotype mimicry in these hosts (Ye et al. 2023).

### 7.12 Square‐Tailed Drongo‐Cuckoo *Surniculus lugubris*

The breeding range of the square‐tailed drongo‐cuckoo extends from northeastern India, northern Myanmar, and northern Thailand to northern Indochina, as well as southern and southeastern China, and southward through the Malay Peninsula to Sumatra, Borneo, and the southwestern Philippines (Payne and Kirwan 2020f). In China, this cuckoo has been documented to parasitize five host species (Yang et al. 2012c; Su et al. 2016; Payne and Kirwan 2020f).

The egg phenotype of the square‐tailed drongo‐cuckoo in its Chinese hosts remains unclear. However, outside of China, the species exhibits egg polymorphism. For instance, in Borneo, the eggs are white with fine purple splotches. In Java, three distinct phenotypes have been observed, corresponding to different hosts: (1) pale salmon‐pink with brownish, purplish, or bluish‐grey markings (associated with *Malacocincla sepiarium*), (2) white with brown markings (associated with *Mixornis flavicollis*), and (3) pure white (associated with *Stachyris melanothorax*) (Payne and Kirwan 2020f).

### 7.13 Northern Hawk‐Cuckoo *Hierococcyx hyperythrus*

The breeding range of the northern hawk‐cuckoo extends from northeastern and southeastern China to Ussuriland and Sakhalin in Russia, and southward to Korea and Japan (del Hoyo et al. 2020b). The host species of this cuckoo in China remain unknown, but seven host species have been recorded in Japan. As these hosts also breed in China, they are considered potential hosts for the northern hawk‐cuckoo in China (Figure 1). In Japan, the parasitic eggs of the northern hawk‐cuckoo have been documented as pale blue; however, other egg phenotypes remain unrecorded or poorly understood (del Hoyo et al. 2020b).

### 7.14 Other Cuckoos in China

In China, the banded bay cuckoo is found in southern Yunnan, southwestern Sichuan, and northeastern Guangxi province (Zheng 2023; Payne and Hansasuta 2024); the violet cuckoo is restricted to southeastern Tibet and southwestern Yunnan province (Payne 2020e; Zheng 2023); the pied cuckoo occurs in southern Tibet (Xizang), southern Yunnan province, and Hong Kong, China (Payne et al. 2021; Zheng 2023); and the common hawk‐cuckoo is only found in southeastern Tibet (Payne 2020c; Zheng 2023).

Due to their limited distribution in China and the difficulty in detecting them, there are no direct records of their parasitic activities in the country. However, the common iora (*Aegithina tiphia*) parasitized by the banded bay cuckoo in India and the little spiderhunter parasitized by the violet cuckoo in Indochina both hosts also breed in China, making them potential hosts for these two cuckoos in China (Cheke et al. 2020; Payne 2020b). In contrast, the direct and potential hosts of the pied cuckoo and common hawk‐cuckoo in China remain undocumented.

## 8 Summary and Perspective

China has a rich diversity of cuckoos and their hosts. A total of 17 cuckoo species have been recorded, with 15 of these species known to parasitize 142 actual and potential host species belonging to 74 genera and 34 families. This diversity makes China an excellent location for studying the interactions and co‐evolution between cuckoos and their hosts.

Despite overlaps in the ranges of host body mass and egg volume utilized by different cuckoo species, cuckoos exhibit adaptive matching in body mass and egg volume relative to their hosts. A significant positive correlation was observed between the body mass of cuckoos and their hosts, as well as between the egg volume of cuckoos and their hosts, after controlling for host species and their phylogeny. Furthermore, there is minimal overlap in the specific host species exploited. Most host species are parasitized by a single cuckoo species. This specialization can be attributed to two main factors: avoidance of interspecific competition and shifts in the availability of suitable hosts over time. Regarding egg polymorphism, cuckoos display a range of egg phenotypes that correlate with the number of their host species. The greater the number of host species, the more pronounced the egg polymorphism in cuckoos. This polymorphism is also linked to co‐evolutionary dynamics, where hosts may develop more varied egg phenotypes to defend against cuckoo parasitism, and cuckoos, in turn, evolve to mimic these colors to counteract host defenses. Although most cuckoos have evolved polymorphic eggs, not all egg morphs match those of their host species. This is likely because some hosts are unsuitable for parasitism, prompting cuckoos to explore new hosts. Given that egg morph evolution is a prolonged process, cuckoo egg morphs may not immediately match those of newly targeted hosts.

Due to the relatively late start of cuckoo research in China, knowledge gaps remain in our understanding of cuckoo species, including the egg phenotype mimicry of cuckoos in relation to different host species and the host species of the chestnut‐winged cuckoo, plaintive cuckoo, northern hawk‐cuckoo, and Hodgson's hawk‐cuckoo, as well as their parasitic patterns. To address these gaps, we emphasize the need for systematic field investigations and comparative studies. Finally, we developed a real‐time parasitism tracking program that enables citizen scientists to contribute observational data, which our team then verifies and incorporates into an updated database of host–parasite interactions (Supporting Information S3). This platform aims to serve as a comprehensive resource for researchers, citizen scientists, and the general public to advance understanding of avian brood parasitism.

## Conflicts of Interest

The authors declare no conflicts of interest.

## Supporting information




**Supplementary Information 1** Appendix: Parasitic cuckoo species and their hosts in China
**Supplementary Information 2** The phylogenetic tree of host species
**Supplementary Information 3** The real‐time tracking program for parasitism records

**SUPPLEMENTARY MATERIAL**: inz213009‐sup‐0002‐SuppMat.docx

## Data Availability

Data from this study are provided in Supporting Information S1.
