# Supplementary material for: Host Exploitation by Cuckoos in China: A Review and Real‐Time Tracking Program for Parasitism Records
Source: Integr Zool. 2025 Jul 17;21(2):218–30. doi: 10.1111/1749-4877.13009 (PMC12971627; doi:10.1111/1749-4877.13009)
Supplement: Supplementary file 1 — Supplementary Information 1 Appendix: Parasitic cuckoo species and their hosts in China Supplementary Information 2 The phylogenetic tree of host species Supplementary Information 3 The real‐time tracking program for parasitism records [file INZ2-21-218-s001.pdf]

SUPPLEMENTARY MATERIAL

Supplementary Information 1

Appendix: Parasitic cuckoo species and their hosts in China

**Cuckoo name**	**Host name**	**Cuckoo state**	**Note contents**	**Cuckoo egg mass (g) and size (mm)**	**Cuckoo egg morph**	**Host egg mass (g) and size (mm)**	**Host egg morph**	**Site and Year**	**Source**
Chestnut-winged Cuckoo *Clamator coromandus*	Hwamei *Garrulax canorus*	Nestling	1C+3H					Guizhou province, May 2005	(Jia et al., 2007)
		Nestling	1C+3H					Guizhou province, May 2005	(Jia et al., 2007)
		Egg			blue		blue		(La Touche, 1927)
		Egg	4C+2H	7.49 ± 0. 14, 26.82 ± 0.73 × 22.50 ± 0. 69	turquoise, blue	5.62 ± 0.74, 26.16 ± 1.03 × 20.50 ± 0.80	light turquoise	Guizhou province, 2013	(Huo et al., 2014)
		Fledgling						Xiamen, 2021	website 1
		Fledgling						Xiamen, 2022	website 2
	Masked Laughingthrush *Pterorhinus perspicillatus*	Egg			blue	27~28×19~21	Dusty blue; Light cyan; Light green with auburn spots	Eastern China	(La Touche, 1927)
	Greater Necklaced Laughingthrush *Pterorhinus pectoralis*	Nestling	1C					Hong Kong, May 2005	(Carey et al., 2001)
		Nestling	1C					Hong Kong, July 1995	(Carey et al., 2001)

	Nestling	1C					Hong Kong, August 1996	(Carey et al., 2001)
	Nestling	1C					Hong Kong, May 1998	(Carey et al., 2001)
	Egg	2C+6H	25.4~29.9×20.3~24.4	blue	28.7~33.8×20.9~24.1	blue	Hubei province, June 2006	(Yang et al., 2012b)
	Fledgling						Yunnan province, 2023	website 3
Oriental Magpie Robin *Copsychus saularis*	Egg		25.4~29.9×20.3~24.4	blue	20.4~23.0×16.1~17.4	Reseda with dark brown spots	Guangdong province	(Guangdong Institute of Entomology, Sun Yat-sen University, 1983)
Chinese Babax *Babax lanceolatus*	Egg	2C+2H	7.49 ± 0. 14, 26.82 ± 0.73 × 22.50 ± 0. 69	turquoise	5.92 ± 0. 62, 27.53 ± 1.03 × 20.53 ± 0.61	deep turquoise	Guizhou province, 2013	(Huo et al., 2014)
*Slaty-backed Forktail *Enicurus schistaceus*			25.4–29·9×20.3–24.4		2.6~2.7, 20.6~22.0×16.2~16.8	White with brown spots	Mainland Southeast Asia	(Erritzøe et al., 2012)
*Lesser Necklaced Laughingthrush *Garrulax monileger*			25.4–29·9×20.3–24.4		28.4×21.3	Dark turquoise	Mainland Southeast Asia	(Erritzøe et al., 2012)

	*Orange-headed Thrush *Geokichla citrina*			25.4–29·9×20.3–24.4		21~28×17~21.6		Mainland Southeast Asia	(Erritzøe et al., 2012)
	*Long-tailed Shrike *Lanius schach*			25.4–29·9×20.3–24.4		6.5~8.1, 22.4~23.7×27.2~30.5		Mainland Southeast Asia	(Erritzøe et al., 2012)
	*Blue Whistling-thrush *Myophonus caeruleus*			25.4–29·9×20.3–24.4		11.3~12.6, 34.9~37.2×24.7~25.6		Mainland Southeast Asia	(Erritzøe et al., 2012)
	*Black-breasted Thrush *Turdus dissimilis*			25.4–29·9×20.3–24.4		21.1~29×18.3~21		Mainland Southeast Asia	(Erritzøe et al., 2012)
Large Hawk-Cuckoo *Hierococcyx sparverioides*	Chinese Babax *Babax lanceolatus*	Egg	1C+2H	6.6, 27.1 × 21.4	white	6.0, 27.8 × 20.6	deep sky blue	Guizhou province, May 2008	(Yang et al., 2012a)
		Egg	1C+2H		white		deep sky blue	Guizhou province, June 1999	(Yang et al., 2012b)
		Egg	1C+1H		white		deep sky blue	Guizhou province, July 1999	(Yang et al., 2012b)
		Egg	1C+1H		white		deep sky blue	Guizhou province, July 1999	(Yang et al., 2012b)
		Egg	1C+2H		white		deep sky blue	Guizhou province, August 1999	(Yang et al., 2012b)
		Egg						Guizhou province	(Yang et al., 2015)

White-browed Laughingthrush *Pterorhinus sannio*	Egg	1C+4 H	6.2, 27.0 × 20.6	white	5.7, 27.8 × 19.7	white	Guizhou province, May 2006	(Jiang et al., 2007)
	Egg	1C+2 H	7.5, 30.4 × 21.8	white	5.3, 26.1 × 20.0	white	Guizhou province, May 2008	(Yang et al., 2012b)
	Egg	1C+1 H		white		blue	Guizhou province July 1999	(Yang et al., 2012b)
	Egg	1C+1 H		white		blue	Guizhou province July 1999	(Yang et al., 2015, 2012b)
	Fledging							website 4
Moustached Laughingthrush *Ianthocincla cineracea*					25~28.5×17.5~20	blue or plain greenish turquoise	Yunnan province	(Liu et al., 2025)
Black-necklaced Scimitar-babbler *Erythrogenys erythrocnemis*							Taiwan province	(Payne and Kirwan, 2020a)
White-bellied Redstart *Luscinia phaenicuroides*	Egg	1C+2 H	6.09, 20.20×27.73	white	2.34, 20.05×14.94; 2.40, 19.95×15.09	blue	Guizhou province, 2015	(Huo et al., 2016)
Elliot's Laughingthrush *Trochalopteron elliotii*	Egg		4.5, 25.0×19.0; 5.0, 27.0×19.5	offwhite with brown spots	5.8, 28.2×20.1	light sky blue with brown spots and steaks	Shanxi province, May 1996	(Fan et al., 2000; Hu et al., 2013; Yi, 2020)
	Egg		6.9,	turquoi	5.8, 28.2×20.1	light sky blue with brown spots	Gansu	(Hu et al.,

			29.76×20.40; 7.2, 28.40×21.68	se			province	2013)
	Fledging						Gansu province	(Hu et al., 2013)
Masked Laughingthrush *Pterorhinus perspicillatus*	Egg		4.5, 25.0×19.0; 5.0, 27.0×19.5	offwhite with brown spots	5.6, 29.0×21.0	light cyan	Shanxi province, May 1996	(Fan et al., 2000)
	Fledging							website 5
	Fledging							website 6
Spot-breasted Scimitar Babbler *Erythrogenys gravivox*	Egg		4.5, 25.0 ×19.0; 5.0, 27.0 × 19.5	offwhite with brown spots	6.5, 28.3×20.7	white	Shanxi province, May 1996	(Fan et al., 2000)
Streak-breasted Scimitar Babbler *Pomatorhinus ruficollis*	Fledging		5.0, 27.0 × 19.5		4~4.5; 24~25×17~18	white	Yunnan province, May 2022	(Gao et al., 2024)
Hwamei *Garrulax canorus*	Nestling	1C					Guangxi province, May 2003	(Yang et al., 2012b)
	Egg	1C+2H	5.0, 27.0 × 19.5	white	3.6~6.3, 24.0~28.4×18.8~21.8	blue	Guizhou province, June 1999	(Yang et al., 2012b)
Oriental Magpie *Pica serica*	Egg	2C+4H	4.7, 26.2 × 19.2	olive grey with brown spots	9~13, 23~26×32~38	light turquoise with brown or black spots	Beijing, June 1973	(Cheng et al., 1991)

	*Little Spiderhunter *Arachnothera longirostra*			17.0~19.1×12.5~13.9	dull white or creamy with zone of heavy red-brown or purple-brown spots	India	(Payne and Kirwan, 2020a)
	*Streaked Spiderhunter *Arachnothera magna*			20.9~24.2×15~16.4	caesious with brown spots	India	(Payne and Kirwan, 2020a)
	*Lesser Shortwing *Brachypteryx leucophris*			18.5~23.0×14~15	olive-green with light reddish-brown freckles	India	(Payne and Kirwan, 2020a)
	*Hainan Blue Flycatcher *Cyornis hainanus*					Mainland Southeast Asia	(Clement and Christie, 2020)
Indian Cuckoo *Cuculus micropterus*	Azure-winged Magpie *Cyanopica cyanus*	Nestling				Jiangsu province, 1974	(Yan, 1985)
		Egg	4.0, 21.0×17.5; 24.0×18.0	6.0, 28.1×20.2	lightcyan with brown and purple spots	Shanxi province, June 1996	(Bu et al., 1999)
		Fledgling				Shandong province, 2009	(Yang et al., 2012b)
		Egg				Shandong province	(Zhang et al., 2017)
		Nestling				Shandong province	(Zhang et al., 2017)
		Fledgling				Shandong province	(Zhang et al., 2017)

Ashy Woodswallow *Artamus fuscus*				22.0~23.4×16.7~17.1	offwhite or pale greenish-white, covered with spots of varying sizes ranging from brownish-yellow to tan, some also interspersed with sparse gray, reddish-brown, and pale purple speckles	Guangdong province	(Liu et al., 2025)
				22.0~23.4×16.7~17.1	offwhite or pale greenish-white, covered with spots of varying sizes ranging from brownish-yellow to tan, some also interspersed with sparse gray, reddish-brown, and pale purple speckles	Guangxi province	(Liu et al., 2025)
Meadow Bunting *Emberiza cioides*	Egg			1.8~2.0, 19~21×15~17	white with brown linear, wavy, or tadpole-like stripes and spots	Shandong province	(Liu et al., 1992)
White-crowned Forktail *Enicurus leschenaulti*				3.61±0.17, 23.55×17.61	white with brown spots	Hainan province	(Liu et al., 2025)
Plain Laughingthrush *Pterorhinus davidi*	Egg	2C	3.4, 23~18×22~17	25~27×19~21	plain turquoise-blue	Shanxi province	(Wang, 2012)
Oriental Reed Warbler *Acrocephalus orientalis*	Egg		4.0, 21.0×17.5; 24.0×18.0	3.8, 22.4×16.0	offwhite with olive spots	Shanxi province, June 1996	(Bu et al., 1999; Hao and Wang, 1992)

	Nestling						Jiangsu province, 1980	(Hao and Wang, 1992; Yan, 1985)
Light-vented Bulbul *Pycnonotus sinensis*	Nestling		5.4, 25.25×20.1		2.6~3.3, 21.5~24×16~16.6	pink with purple spots	Jiangsu province, 1983	(Liu and Long, 1986; Yan, 1985)
Black Drongo *Dicrurus macrocercus*	Egg	1C+2H		pinkish white with firebrick spots	4.8, 24.7×18.6	lightpink with brown spots	Hong Kong	(Vaughan and Jones, 1913; Zhang, 2001)
	Fledgling						Guangxi province, July 2019	website 7
	Fledgling						Shandong province, July 2024	website 8
Chinese Blackbird *Turdus mandarinus*	Egg	1C+2H	6.8, 29.5×22.7	offwhite with firebrick spots	7.2, 29.1×21.4	lightcyan with brown spots	Anhui province, April 1996	(Zhang, 2001)
Siberian Stonechat *Saxicola torquata*	Nestling				1.5~2.1, 16~19.8×12~15.6	turquoise with reddish-brown spots	Yangtze Basin	(Cheng et al., 1991)
Black-naped Oriole Oriolus chinensis	Fledgling						Guangdong province, 2023	(Liu et al. 2025); website 9
Brown Shrike *Lanius cristatus*	Fledgling				3.1~3.5, 15~19×21.1~24.5	White with fulvous spots; Grey with fulvous spots	Jilin province,	(Payne, 2020a);

								2023	website 10
	*Grey Drongo *Dicrurus leucophaeus*					18~20×23~26	light pink with grey, dark red, claybank, and black brown spots	India	(Payne, 2020a)
	*Greater Racquet-tailed Drongo *Dicrurus paradiseus*					26~32.4×20~22.5	white; white with light red spots	Indonesia	(Payne, 2020a)
Common Cuckoo *Cuculus canorus*	Ashy-throated Parrotbill *Suthora alphonsiana*	Egg	1C+4H	2.6, 20.8×16.2	turquoise	1.3, 16.1×12.7	turquoise	Guizhou province, June 2008	(Yang et al., 2010b)
		Nestling	1C					Guizhou province, June 2008	(Yang et al., 2012b)
		Nestling	1C					Guizhou province, July 2008	(Yang et al., 2012b)
		Egg	1C+2H		blue, pale blue, white		blue, pale blue, white	Guizhou province, June	(Yang et al., 2010b)
		Egg	1C+3H		turquoise		turquoise	Guizhou province, June 1999	(Yang et al., 2010b)
		Nestling	1C					Guizhou province, July 1999	(Yang et al., 2012b)
		Nestling	1C					Guizhou province, August 2006	(Yang et al., 2012b)

	Nestling	1C					Guizhou province, June 2005	(Yang et al., 2012b)
	Nestling	1C					Guizhou province, June 2007	(Yang et al., 2012b)
	Egg	1C	3.0, 21.4×16.2	pale blue			Guizhou province, June 2011	(Yang et al., 2012b)
	Egg	1C+2H	3.1, 21.6×16.2	white	1.2, 16.7×12.4	paleblue	Guizhou province, June 2011	(Yang et al., 2012b)
Red-billed Blue Magpie *Urocissa erythrorhyncha*					5.6~9.2, 28.1~32.5×21.2~23.2	earthy yellow with reddish-brown spots	Henan province	(Guo et al., 2022)
Yellow-throated Bunting *Emberiza elegans*	Egg	1C+1H	3.21, 22.32×17.32	light turquoise	2.08±0.18, 19.15±0.87×15.01±0.29	offwhite with black spots	Guizhou province	(Zhang et al., 2023)
Crested Bunting *Emberiza lathami*					17.9~22.0×13.0~17.0	offwhite with reddish brown spots	Yunnan province	(Liu et al. 2025)
Southern Rock Bunting *Emberiza yunnanensis*	Egg	1C+1H	3.26, 22.35×17.78	light turquoise	2.61±0.25, 20.58±0.83×15.62±0.50	offwhite with reddish brown spots and lines	Guizhou province	(Zhang et al., 2023)
Swinhoe's White-eye *Zosterops simples*					1.09±0.09, 15.93±0.55×11.89±0.46	white	Yunnan province	(Liu et al. 2025)

Siberian Rubythroat *Calliope calliope*				2.0~2.2, 19.0~20.5×15.0~16.5	turquoise	Qinghai province	(Liu et al. 2025)
Great Reed Warbler *Acrocephalus arundinaceus*				21~25×15~17	turquoise with brown spots, some with small charcoal grey or grey spots	Xinjiang province	(Liu et al. 2025)
White-throated Redstart *Phoenicurus schisticeps*				19~20×15~16	pink with brown spots	Xizang province	(Liu et al. 2025)
Red-tailed Shrike *Lanius phoenicuroides*	Egg	1C+2H	white with brown spots	21~24×15.6~17.8	pink with brown spots	Xinjiang province, 2022	website 34
Vinous-throated Parrotbill *Suthora webbianus*	Fledgling					Sichuan province	(Yang et al., 2012b)
	Egg		turquoise with violet spots	1.5~2, 15~19×12~13.4	white; light blue; turquoise; pastel green	Shaanxi province	(Cheng, 1973, 1963)
	Fledgling					Shandong province, 2023	website 11
Reed Parrotbill *Paradoxornis heudei*	Egg	1C+2H	offwhite with olive spots	1.6~2.1, 18.0~19.0×14.0~14.8	offwhite with olive spots	Shandong province, June 2008	(Yang et al., 2012b)
	Fledgling					Shandong province, 2023	website 12

Daurian Redstart *Phoenicurus auroreus*	Nestling	1C					Guizhou province, June 2005	(Yang et al., 2012b)
	Egg	1C+4 H	2.8, 20.8 × 16.3	white with grey spots	1.7, 17.9 × 13.7	pale turquoise with firebrick spots	Guizhou province, July 1999	(Yang et al., 2012b)
	Egg	1C+5 H	2.8, 20.8 × 16.3	white with grey spots	1.7, 17.9 × 13.7	white with firebrick spots	Guizhou province, June 1992	(Yang et al., 2012b)
	Egg	1C+3 H					Guizhou province, June 1992	(Yang et al., 2012b)
	Nestling	1C					Guizhou, province June 1992	(Yang et al., 2012b)
	Egg	1C+3 H		white with grey spots		white with firebrick spots	Guizhou province, July 1994	(Yang et al., 2012b)
	Nestling	1C					Guizhou province, May 2008	(Yang et al., 2012b)
	Nestling	1C					Shaanxi province	(Cheng, 1973, 1963)
	Fledgling						Shaanxi province, 2020	website 13
Black Redstart *Phoenicurus ochruros*	Nestling	1C			18~21×13~15.1	light turquoise; light turquoise with black-brown spots	Qinghai province, July 2008	(Yang et al., 2012b)

	Fledgling						Gansu province, August 2005	(Yang et al., 2012b)
	Fledgling						Xizang province, 2020	website 14
White-bellied Redstart *Luscinia phaenicuroides*	Egg	1C+3H	3.8, 25.9×17.9	dark turquoise	2.0~3.2, 18.6~23.6×15.0~16.9	dark turquoise	Xizang province, 1999	(Yang et al., 2012b)
	Egg	1C+3H	3.8, 25.1×18.0	dark turquoise	2.0~3.2, 18.6~23.6×15.0~16.9	dark turquoise	Xizang province, 1999	(Yang et al., 2012b)
	Egg		3.76±0.16, 22.19±0.52×17.62±0.47	light blue; dark turquoise	2.87±0.23, 21.66±1.03×15.97±0.44	dark turquoise	Gansu province, 2009~2012	(Hu et al., 2013)
Plumbeous Water Redstart *Phoenicurus fuliginosus*	Nestling	1C			1.9~2.4, 18.6~20.0×14.2×15.2	white with brown spots	Jiangsu province	(Yang et al., 2012b)
	Fledgling							website 15
	Fledgling						Shannxi province, 2013	website 16
Bluethroat *Luscinia svecica*	Nestling				17~21×13~15	pale green with brown spots	Xinjiang province	(Qian and Zhang, 1965)
Siberian Stonechat *Saxicola*	Fledgling						Guizhou province, July 2007	(Yang et al., 2012b)

maurus	Egg		3.83, 23.18×17.38	light taupe with dark brown spots	1.81, 17.48×13.80	turquoise with orange-brown spots and light brown striolae	Hebei province, 2012~2015	(Wang, 2018)
	Nestling						Hebei province, 2012~2015	(Wang, 2018)
Grey Bushchat *Saxicola ferreus*	Egg	1C+3H	2.7, 20.1×16.0	dark turquoise	1.9, 17.6×14.4	medium turquoise	Guizhou province, May 2007	(Yang et al., 2012b)
	Egg	1C+4H					Guizhou province, August 1999	(Yang et al., 2012b)
White Wagtail *Motacilla alba*	Fledgling						Guizhou province, July 2006	(Yang et al., 2012b)
	Nestling	1C					Sichuan province	(Li, 1985)
	Egg	1C+2H	3.89, 23.40×17.46	white with light brown spots	2.21±0.21, 20.46±0.52×15.19±0.44	white with brown spots	Guizhou province, 2020	(Liu et al., 2022)
Richard's Pipit *Anthus richardi*	Fledgling						Qinghai province, 2007	(Yang et al., 2012b)
Oriental Reed Warbler	Nestling						Shanxi province,	(Hao and Wang, 1992;

Acrocephalus orientalis							June 1985	Lu, 1988)
	Fledgling						Heilongjiang province, July 2007	(Yang et al., 2012b)
	Fledgling						Liaoning province	(Yang et al., 2012b)
	Egg		4.4, 24.0×18.0	offwhite with brown spots	3.8, 22.4×16.0	offwhite with olive spots	Shandong province, 1984-1986	(Tian et al., 1991)
	Fledgling						Beijing province, 2011	(Yang et al., 2012b)
Blunt-winged Warbler *Acrocephalus concinens*	Egg		21.8× 16.22	offwhite with brown spots	15.8~18×11.8~12.8	Light green with tawny spots	Hebei province, 2016-2017	(Ma, 2018)
Black-browed Reed Warbler *Acrocephalus bistrigiceps*	Egg	1C+4H	3.6, 25.0×17.3	lightcyan with brown spots	1.3, 13.8×10.7	offwhite with brown spots	Shanxi province, June 1981	(Liu et al., 1984)
Pale-legged Leaf Warbler *Phylloscopus tenellipes*	Egg		1.8, 21.5 × 13.3	pinkish white with sandy brown spots	1.5, 15.6 × 12.3	pinkish white	Jilin province, June 1979	(Gao, 2004)
	Nestling							(Fu et al., 1984)

Zitting Cisticola *Cisticola juncidis*	Egg			turquoise with violet spots	13.8~16.8×10.2~12.3	white with rufous spots; light blue with rufous spots	North China	(Cheng et al., 1991)
	Fledgling							website 17
Isabelline Shrike *Lanius isabellinus*	Fledgling						Xinjiang province	(Yang et al., 2012b)
	Fledgling						Xinjiang province	(Yang et al., 2012b)
	Fledgling						Shanxi province, 2011	(Yang et al., 2012b)
	Egg	1C+5H	24.11~25.78×16.27~17.76	White	21.04±0.31×16.83±0.18	White with brown spots; White with light brown spots; Pink with reddish brown spots	Gansu province, 2010	(Ma et al., 2012)
	Nestling	1C					Gansu province, 2010	(Ma et al., 2012)
	Fledgling						Gansu province, 2010	(Ma et al., 2012)
Brown Shrike *Lanius cristatus*	Eggs				3.1~3.5, 15~19×21.1~24.5	white with fulvous spots; grey with fulvous spots	Xinjiang province	(Qian and Zhang, 1965)
	Fledgling						Beijing, 2011	(Yang et al., 2012b)
	Fledgling						Henan province, 2011	(Yang et al., 2012b)
Grey-backed Shrike *Lanius tephronotus*	Fledgling				22.7~27.0×18.0~19.3	offwhite with brownness and purple spots	Chongqing, 2009	(Yang et al., 2012b)

Lesser Grey Shrike *Lanius minor*	Fledgling				23~28×16~20	light yellow or light green with light brown or purplish-brown spots	Xinjiang province, 2021	website 18
Blue-and-white Flycatcher *Cyanoptila cyanomelana*	Nestling				1.2~2.8, 16~23×14~17	white; white with light brown spots	Jilin province	(Fu et al., 1984)
Black-faced Bunting *Emberiza spodocephala*	Fledgling				1.3~2.1, 18~21×13~16	offwhite with reddish-brown spots; white with chestnut brown spots; light blue with red spots	Qinghai province, 2007	(Yang et al., 2012b)
	Nestling							(Fu et al., 1984)
Jankowski's Bunting *Emberiza jankowskii*	Fledgling						Heilongjiang province, 2005	(Yang et al., 2012b)
	Egg	1C+5H			2.0~2.5, 19.5~21×15~18	offwhite with brown spots	Jilin province, 2011	(Wang et al., 2011)
Azure-winged Magpie *Cyanopica cyanus*	Egg	1C+4H	5.4, 26.0×20.0	lightcyan with brown spots	5.7, 27.4×19.3	lightcyan with brown and purple spots	Shandong province, April 1979	(Zhang, 1989)
Desert Finch *Rhodospiza obsoleta*	Nestling				17.4~22.0×12.0~15.1	light blue with purplish black spots; white; turquoise	Xinjiang province	(Qian and Zhang, 1965)

Barn Swallow *Hirundo rustica*	Fledgling		1.3~2.5, 13~16×18~20	White with reddish-brown spots	Gansu province, 2007; Hebei province, 2007, 2010; Jilin province, 2011; Shanxi; Shandong ; Heilongjiang province	(Su et al., 2017)
Barred Warbler *Curruca nisoria*	Fledgling		18~22.6×14.5~16.3	white with grey spots; white with lavender spots	Xinjiang province, 2021	website 32; (Yang et al., 2023)
Oriental Magpie Robin Copsychus saularis	Fledgling					website 19
	Fledgling		20.4~23.0×16.1~17.4	Reseda with dark brown spots	Jiangxi and Hubei province, August 2022	(Lin et al., 2024)
	Fledgling					website 20
Meadow Bunting Emberiza cioides	Fledgling		1.8~2.0, 19~21×15~17	white with brown spots	Shandong province, 2023	website 21
Tree Sparrow Passer montanus	Fledgling		2~2.6, 17.1~21.5×12.6~15.4	offwhite with purplish brown spots	Yunnan province, 2021	website 22

									(Yang et al., 2023)
	Eurasian Wren Troglodytes troglodyte	Nestling				1~1.4, 16.4~18.1×10.3~13.0	pink with brown spots; white with reddish-brown spots	Yunnan province, 2021	website 22
	Chestnut-bellied Rock-Thrush *Monticola rufiventris*	Fledgling					creamy-white with reddish-brown speckles	Yunnan province, 2024	website 35
	Buff-throated Warbler Phylloscopus subaffinis	Fledgling				0.7~1.2, 13.6~15.7×10.9~12.8	white	Chongqing province, 2017	website 23
		Fledgling							website 24
	*Little Spiderhunter *Arachnothera longirostra*					17.0~19.1×12.5~13.9	creamy with red-brown or purple-brown spots	Thailand	(Cheke et al., 2020)
Himalayan Cuckoo *Cuculus saturatus*	Blyth's Leaf Warbler *Phylloscopus reguloides*	Egg		21.5×14.0	white with a few brown spots	1.0, 14.9×12.2	white	Fujian province, 1931	(La Touche, 1927)
		Egg	1C+3H	2.1, 20.6×13.5	white with a few brown spots	1.1, 14.4×11.8	white	Guizhou province, June 2009	(Yang et al., 2011)
		Egg						Fujian province	(La Touche, 1927)

Bianchi's Warbler *Phylloscopus valentini*	Nestling				1.2, 15.8 × 12.0	white	Guizhou province, 2018	(Lin et al., 2024)
Brownish-flanked Bush Warbler *Cettia fortipes*	Egg	1C+1H	2.5, 23.4×14.4	white with a few brown spots	1.5, 17.7×13.0	chocolate	Guizhou province, May 2011	(Yang et al., 2012b)
Slaty-backed Forktail *Enicurus schistaceus*	Nestling				2.6~2.7; 20.6~22.0×16.2~16.8	white with brown spots	Eastern China	(La Touche, 1927)
Yellow-throated Bunting *Emberiza elegans*	Egg	1C+2H	2.7, 22.8×14.9	white with a few brown spots	1.4, 16.4×13.1	offwhite with black sptos	Guizhou province, May 2011	(Yang et al., 2012b)
	Nestling							(Fu et al., 1984)
	Egg	1C+1H	2.39±0.14, 21.45±1.03×14.29±0.24	White with a few brown spots	2.0±0.23, 18.44±0.70×14.71±0.55	offwhite with black sptos	Guizhou province, June 2012	(Su et al., 2014)
Swinhoe's White-eye *Zosterops simples*	Egg	1C+2H	2.39±0.14, 21.45±1.03×14.29±0.24	White with a few brown spots	1.09±0.09, 15.93±0.55×11.89±0.46	white	Guizhou province, June 2012	(Su et al., 2014)

	Buff-throated Warbler *Phylloscopus subaffinis*	Egg	1C+3H	2.39±0.14, 21.45±1.03×14.29±0.24	White with a few brown spots	1.09±0.08, 15.08±0.32×11.75±0.35	white	Guizhou province, June 2012	(Su et al., 2014)
	Collared Finchbill *Spizixos semitorques*	Egg	1C+2H	3.0, 21.2×16.1	white with a few brown spots	3.3, 24.6×17.1	pink with violet spots	Guizhou province, June 2011	(Yang et al., 2012b)
	Large-billed Leaf Warbler *Phylloscopus magnirostris*	Fledgling				16~20×12.7~13.9	white	Weining, Guizhou, 2013	(Yang et al., 2023)
Oriental Cuckoo *Cuculus optatus*	Yellow-rumped Flycatcher *Ficedula zanthopygia*	Egg			white	1.5~2, 15.0~19.0×11.8~14.5	white with rufous spots	Beijing	(Deng, 2013)
	Siberian Stonechat *Saxicola torquata*	Nestling				1.5~2.1, 16~19.8×12~15.6	turquoise with reddish-brown spots	Eastern China	(La Touche, 1927)
	Lesser Shortwing *Brachypteryx leucophrys*	Egg		21.0×14.5	turquoise with red spots	18.5~23.0×14~15	olive-green to sea-green with light reddish-brown freckles	Eastern China	(La Touche, 1927)
	Humes' Warbler *Phylloscopus humei*	Egg	1C+3H	2.5, 20.7×14.0	white	1.1, 15.0×11.1	white	Gansu province, June 2002	(Wang et al., 2004)
	Pale-legged Leaf Warbler	Nestling						Jilin province	(Fu et al., 1984)

Phylloscopus tenellipes	Egg			1.5~2.3, 14~17×11.6~12.3	white	Jilin province	(Meshcheryagina et al., 2018)
Eastern Crowned Warbler *Phylloscopus coronatus*	Nestling					Heilongjiang province, June 1987	(Gao et al., 1990)
	Egg		White	15.5~17×12~13	white	Beijing, 2013	(Wang et al., 2014)
	Nestling					Beijing, 2013	(Wang et al., 2014)
	Fledgling					Beijing, 2013	(Wang et al., 2014)
White Wagtail *Motacilla alba*	Fledgling			2.4~2.8, 19.8~22.2×14.9~15.7	white with brown spots and microgroove	Xinjiang province, July 2002	(Yang et al., 2012b)
Yellow-bellied Prinia *Prinia flaviventris*	Egg	21.0×14.7	pink with dense red spots	2.4~2.8, 19.8~22.2×14.9~15.7	pink with dense red spots	Taiwan province	(La Touche, 1927)
	Egg		white with brown spots	13.8~15.0×11.2~12.0	pink with dense red spots	Taiwan province	(Zhang, 1980)
	Egg	2.1, 18.86×13.83	chocolate	13.8~15.0×11.2~12.0	chocolate	Taiwan province, 2010	(Wang, 2015)
	Fledgling					Yunnan province, 2021	website 22

Rufous-capped Babbler *Cyanoderma ruficeps*	Egg	1C+2H	brown	1.0~1.7, 14.1~17.9×11.8~13.6	white	Taiwan province	(Lin, 2008)
*Lanceolated Warbler *Locustella lanceolata*	Egg			16.2~19×12.6~14.1	white with reddish-brown spots	Russia	(Meshcheryagina et al., 2017)
*Tree Pipit *Anthus trivialis*	Egg			1.8~2.0, 14.5~17×20~23.3	pale blue with fuchsia spots	Russia	(Payne and Kirwan, 2020b)
*Black-throated Accentor *Prunella atrogularis*	Egg			17.4~21.3×13.4~16.8	light blue	Russia	(Meshcheryagina et al., 2017)
*Daurian Redstart *Phoenicurus auroeus*				1.7, 17.9 × 13.7	pale turquoise with firebrick spots; white with firebrick spots	South Korea	(Kim, 2011)
*Japanese Paradise-flycatcher *Terpsiphone atrocaudata*				17~21×14~15	white with rufous spots; white with caesious spots	South Korea	(Kim, 2011)
*Asian Stubtail *Urosphena squameiceps*	Egg			1.7~1.9, 16.5~17.0×12.5~13.5	grey with reddish-brown spots	Japan	(Higuchi, 1998; Payne and Kirwan, 2020b)
*Arctic Warbler *Phylloscopus borealis*	Egg			15~17.5×12~12.5	white with pale pink spots	Russia	(Meshcheryagina et al., 2018)

*Common Chiffchaff *Phylloscopus collybita tristis*	Egg	14~15×11~12.7	white with brown spots	Russia	(Meshcheryagina et al., 2018)
*Yellow-browed Warbler *Phylloscopus inornatus*	Egg	0.9~1.0, 12.5~16.0×11.5~12.0	white with reddish-brown spots	Russia	(Meshcheryagina et al., 2018)
*Pallas's Leaf Warbler *Phylloscopus proregulus*	Egg	0.9~1.0, 12.5~16.0×11.5~12.0	white with reddish-brown spots	Russia	(Meshcheryagina et al., 2018)
*Dusky Warbler *Phylloscopus fuscatus*	Egg	15~18×12~13	white	Russia	(Meshcheryagina et al., 2017)
*Radde's Warbler *Phylloscopus schwarzi*	Egg	1.7~2.1, 16.8~19.0×13.0~14.5	white with brown spots	Russia	(Meshcheryagina et al., 2017)
*Greenish Warbler *Phylloscopus trochiloides*	Egg	14.0~17.1×11.2~12.3	white	Russia	(Meshcheryagina et al., 2017)
*Red-flanked Bluetail *Tarsiger cyanurus*	Egg	2.0~2.5, 17.5~18×13.0~14.5	white with reddish-brown spots	Russia	(Meshcheryagina et al., 2017)
*Common Rosefinch *Carpodacus erythrinus*	Egg	18.7~22×13.2~15.2	light turquoise with brown spots	Russia	(Meshcheryagina et al., 2017)

	*Black-faced Bunting *Emberiza spodocephala*	Egg				1.3~2.1, 18~21×13~16	offwhite with reddish-brown spots	Russia	(Meshcheryagina et al., 2017)
	*Tristram's Bunting *Emberiza tristrami*	Egg				2.0~2.2, 15~18×18~21	gray with dark-brown spots; light turquoise with dark-brown spots	Russia	(Meshcheryagina et al., 2017)
	*Chestnut Bunting *Emberiza rutila*	Egg				17.0~18.7×13.7~14.5	white with brown spots; light blue with brown spots	Russia	(Meshcheryagina et al., 2017)
Lesser Cuckoo *Cuculus poliocephalus*	Brownish-flanked Bush Warbler *Horornis ffortipes*	Egg	1C+3H	2.6, 22.1×15.2	chocolate	1.5, 17.2×13.4	chocolate	Guizhou province, July 2008	(Yang et al., 2010a)
		Nestling						Guizhou province, June 2006	Jiang et al. 2006
		Nestling						Guizhou province, June 2004	(Yang et al., 2012b)
		Nestling						Guizhou province, June 2006	(Yang et al., 2012b)
		Egg	1C+2H	2.6, 22.1×15.2	chocolate	1.5, 17.7×13.0	chocolate	Guizhou province, July 1999	(Yang et al., 2010a)
		Egg	1C+3H		chocolate		chocolate	Guizhou province, July 1999	(Yang et al., 2010a)
		Egg	1C+3H		chocolate		chocolate	Guizhou province, June 1999	(Yang et al., 2010a)

	Egg			chocolate		chocolate	Hubei province, July 2006	(Yang et al., 2012b)
Eurasian Wren *Troglodytes troglodyte*	Nestling						Sichuan province, August 1985	(Li, 1985)
	Egg			chocolate	1~1.4, 16.4~18.1×10.3~13.0	pink with brown spots; white with reddish-brown spots	Japan	(Higuchi, 1998)
Blue-and-white Flycatcher *Cyanoptila cyanomelana*	Egg	1C+2H		lightcyan	2.3, 20.0×15.0	white	Jilin province, 1978	(Zhao, 1985; Zhao and He, 1981)
Manchurian Bush Warbler *Horornis canturians*							Shandong province	(Liu et al., 2025)
Aberrant Bush Warbler *Horornis flavolivaceus*	Fledgling				16.1~18.5×11.8~13.1	offwhite with chestnut spots	Sichuan province, August 2006	(Yang et al., 2012b)
Pygmy Wren Babbler *Pnoepyga pusilla*	Egg	1C+2H	2.1, 20.57×15.74	chocolate	1.2, 18.64×13.55; 1.1, 17.93×13.49	white	Sichuan province, 2015	(Wang et al., 2016)
Buff-throated Warbler *Phylloscopus subaffinis*				chocolate	0.7~1.2, 13.6~15.7×10.9~12.8	white	Guizhou province	(Yang et al., 2023)

	*Blyth's Leaf Warbler *Phylloscopus reguloides*	Egg		chocolate	0.9~1.2, 13.4~16.2×11.4~12.1	white	India	(Ali and Ripley, 1973)
	*Pale-footed Bush Warbler *Hemitesia pallidipes*	Egg		chocolate	16~18.2×12~14.0	erythrinus; chocolate	India	(Becking, 1981)
	*Tickell's Leaf Warbler *Phylloscopus affinis*	Egg		chocolate	15~16.5×11.5~13	white with chocolate spots	India	(Marchetti, 1992)
Plantive Cuckoo *Cacomantis merulinus*	Common Tailorbird *Orthotomus sutorius*	Fledgling					Guangdong province, July 2004	(Yang et al., 2012b)
		Egg	1.45, 18.3×13.5	white with light rufous spots		white with light rufous spots	Hong Kong	(Vaughan and Jones, 1913)
		Fledgling					Guangdong province, May 2006	(Yang et al., 2012b)
		Nestling					Guangxi province, July 2004	(Yang et al., 2012b)
		Nestling					Guangxi province, April 2005	(Yang et al., 2012b)

	Egg	1C+3H		palegreen with light rufous spots		pale green with light rufous spots	Guangxi province, May 2011	(Yang et al., 2012b)
	Egg	1C+4H		white with light rufous spots		white with light rufous spots	Guangxi province, May 2011	(Yang et al., 2012b)
	Fledgling						Guangxi province, 2011	(Yang et al., 2012b)
	Fledgling						Guangdong province, 2023	website 25
Mountain Tailorbird *Phyllergates cuculatus*	Egg	1C+2H; 1C+3H	1.45±0.09, 19.07×13.53	white with brown spots	1.05±0.03	light blue; white	Guangxi province	(Huang et al., 2015)
Dark-necked Tailorbird *Orthotomus atrogularis*	Egg	1C+3H	1.45±0.09, 17.8-18.5×13.0-13.7	white with brown spots	0.89±0.02, 14.6~16.2×10.8~12.5	white with reddish-brown spots	Guangxi province	(Huang et al., 2015)
Striated Prinia *Prinia striata*	Fledgling				1.5, 17~19×11~13	light blue with ochre spots; white, pink, light blue or turquoise with light red, rufous or black spots	Yunnan province	(Guo et al., 2024a)

	Rufescent Prinia *Prinia rufescens*	Egg		white with brown spots; blue with brown spots		immaculate white; white with brown spots; immaculate blue; blue with brown spots	South China	(Liang et al., 2017)
	*Yellow-bellied Prinia *Prinia flaviventris*				13.8~15.0×11.2~12.0	pink with dense red spots	Indonesia	(Payne and Kirwan, 2020c; Well, 1999)
	*Hill Prinia *Prinia superciliaris*				14.4~18.4×11.9~13.6	white; pink	Indian	(Payne and Kirwan, 2020c)
	*Grey-breasted Prinia *Prinia hodgsonii*				14.7×11.7	white; blue; pinkish-white with light reddish-brown spots	Indian	(Payne and Kirwan, 2020c)
	*Plain Prinia *Prinia inornata*				0.9~1.0, 15.4~15.9×11.4~11.7	reseda with large brown spots	Indian	(Payne and Kirwan, 2020c; Yang et al., 2023)
	*Zitting Cisticola *Cisticola juncidis*				13.8~16.8×10.2~12.3	white with rufous spots; light blue with rufous spots	Indian	(Payne and Kirwan, 2020c)
	*Crimson Sunbird *Aethopyga siparaja*				14.3~16.3×11.2~12.0	white with purplish brown spots; grey with purplish brown spots	Indonesia	(Payne and Kirwan, 2020c)
Asian Emerald	Bianchi’s Warbler	Nestling	1C		1.2, 15.8×12.0	white	Guizhou province,	(Yang et al., 2008)

Cuckoo *Chrysococcyx maculatus*	*Phylloscopus valentini*							June 2007	
		Egg	1C+1H	1.5, 17.2×12.4	white with brown spots	1.3, 15.0×12.3	white	Guizhou province, June 2011	(Yang et al., 2012b)
		Nestling	1C					Guizhou province, June 2011	(Yang et al., 2012b)
		Nestling	1C					Guizhou province, June 2011	(Yang et al., 2012b)
		Nestling	1C					Guizhou province, June 2011	(Yang et al., 2012b)
		Nestling	1C					Guizhou province, June 2011	(Yang et al., 2012b)
		Nestling	1C					Guizhou province, June 2011	(Yang et al., 2012b)
	Chestnut-crowned Warbler *Phylloscopus castaniceps*	Egg	1C+4H	1.3, 17.0×12.8	white with brown spots	0.9, 15.0×11.1	white	Guizhou province, June 2011	(Yang et al., 2012b)
	Blyth's Leaf Warbler *Phylloscopus reguloides*	Nestling	1C			1.0, 14.9 × 12.2	White	Guizhou province, June 2011	(Yang et al., 2012b)
	Grey-crowned Warbler *Phylloscopus tephrocephalus*							Yunnan province	(Liu et al. 2025)

	Buff-throated Warbler *Phylloscopus subaffinis*	Nestling						Sichuan province, 1985	(Li, 1985)
		Egg	1C+3H	1.5, 17.2×12.4	white with brown spots	0.7~1.2, 13.6~15.7×10.9~12.8	white	Guizhou province, July 2020	(Lin et al., 2024; Zhang et al., 2021)
		Nestling						Guizhou province, July 2020	(Zhang et al., 2021)
		Fledgling						Guizhou province, June 2020	website 26
	White-throated Fantail *Rhipidura albicollis*	Fledgling				16.1~18.2×12.0~13.5	white with brown spots	Guangxi province, July 2012	(Jiang, 2013; Jiang et al., 2014)
	White-crowned Forktail *Enicurus leschenaulti*	Egg/Fledgling	1C+2H	1.84 ± 0.54; 19.08×13.37	white with brown spots	3.61 ± 0.17; 23.55×17.61	white with brown spots	Guizhou province, April 2018	(Lin et al., 2024)
	*Crimson Sunbird *Aethopyga siparaja*					14.3~16.3×11.2~12.0	white with purplish brown spots	India	(Payne, 2020b)
	*Little Spiderhunter *Arachnothera longirostra*					17.0~19.1×12.5~13.9	creamy with zone of heavy red-brown or purple-brown spots	India	(Payne, 2020b)
Square-tailed Drongo-C	David's Fulvetta *Alcippe davidi*	Fledgling				1.7~2.6, 18.0~20.5×14.2~15.6	white with small reddish-brown spots	Fujian province, June 2011	(Yang et al., 2012b)

uckoo *Surniculus lugubris*	Rufous-capped Babbler *Cyanoderma ruficeps*	Fledgling			1.0~1.7, 14.1~17.9×11.8~13.6	white; white with small brown spots and lines	Guangxi province, July 2013	Su et al. 2016
		Fledgling					Hainan province, 2021	website 27
		Fledgling					Guangdong province, July 2020	unpablished data
	Red-whiskered Bulbul *Pycnonotus jocosus*				2.81, 20~24×15~18	pink, densely covered with dark red and pale purple spots, particularly prominent at the blunt end	Guangxi province	(Liu et al. 2025)
	Black Drongo *Dicrurus macrocercus*				24~26×18~20	white with reddish-brown spots; pink with reddish-brown spots	China	(Payne and Kirwan, 2020d)
	Hair-crested Drongos *Dicrurus hottentottus*				6~8, 25~34.5×19.8~23	pale cream with longitudinal blotches of rather pale reddish brown, lilac or purple-red, also sparsely with deep purple freckles	China	(Payne and Kirwan, 2020d)
	*Striped Tit-babbler *Mixornis gularis*				16.6~17.1×12.7~13	white with rufous spots	Malaysia	(Payne and Kirwan, 2020d)
Asian Koel *Eudynamys scolopaceus*	Black-collared Starling *Gracupica nigricollis*	Egg	33.5×22.9	sage green	29.4×21.7	turquoise	Hong Kong	(Vaughan and Jones, 1913)
		Egg					Hong Kong, April 1931	(Carey et al., 2001)

	Fledgling					Hainan province, August 2023	website 28
Red-billed Blue Magpie *Urocissa erythrorhyncha*	Nestling	1C+2H				Sichuan province, June 1963	(Li, 1985)
	Nestling					Hong Kong	(Lewthwaite, 1996)
	Fledgling		33.5×22.9	7~8, 31~36×23~24	earthy yellow with reddish-brown spots	Guangdong province, June 2022	website 29
Eurasian Jay *Garrulus glandarius*				8.5, 28.5~33.0×22.0~24.5	Bluish-gray, green, or yellowish-green with purplish-brown, grayish-brown, or yellowish-brown spots, particularly dense at the blunt end	Guangxi province	(Liu et al. 2025)
Taiwan Blue Magpie Urocissa caerulea	Fledgling					Taiwan province	website 22; website 36
Oriental Magpie *Pica serica*	Nestling	1C		9~13, 23~26×32~38	light turquoise with brown or black spots	Hong Kong	(Carey et al., 2001)
	Nestling	1C				Hong Kong	(Carey et al., 2001)
Azure-winged Magpie *Cyanopica cyanus*	Nestling	2C		6.0, 28.1×20.2	grey with brown spots	Hubei province, August 2021	(Lin et al., 2024); website 30

Masked Laughingthrush *Pterorhinus perspicillatus*	Nestling	1C			27~28×19~21	light cyan; light green with auburn spots	Hong Kong	(Carey et al., 2001)
House Crows *Corvus splendens*	Egg		8.18±0.35, 28.08 ± 0.66×22.05 ± 0.59	Turquoise with numerous brownish spots	35~37×26~27	Turquoise with numerous brownish spots	Indonesia	(Begum et al., 2011; Jadav and Parasharya, 2014)
	Nestling							(Jadav and Parasharya, 2014)
	Fledging						China	website 22
*Long-tailed Shrike *Lanius schach*	Egg			turquoise with numerous brownish spots; celadon with numerous brownish spots	6.5~8.1, 22.4~23.7×27.2~30.5	pale green with rufous spots; white with rufous spots; pink with rufous spots	Thailand	(Begum et al., 2011; Limparungpatthanakij, 2020)

	*Common Mynas *Acridotheres tristis*	Egg		turquoise with numerous brownish spots	27.6~35×19.2~23.2	turquoise	Thailand	(Begum et al., 2011)
	*Great Mynas *Acridotheres grandis*		8.18±0.35, 28.08 ± 0.66× 22.05 ± 0.59		26.8~31×19.8~21.9	blue	Thailand	(Limparungpatthanakij, 2020)
	*Large-billed Crow *Corvus macrorhynchos*	Egg	8.79 ± 0.83, 29.97 ± 1.16× 22.97 ± 0.84		41~48.8×27.4~30.2	dark turquoise with taupe spots	Indonesia	(Jadav and Parasharya, 2014)
		Nestling						(Jadav and Parasharya, 2014)
Hodgson's Hawk-cuckoo *Hierococcyx nisicolor*	Hainan Blue Flycatcher *Cyornis hainanus*	Fledging	22·5×15·4				Guangxi province, Junly 2013	(Su et al., 2016)
	White-rumped Shama Copsychus malabaricus	Fledging			3.05±0.20, 21.68±0.63×16.37±0.47	turquoise with numerous brownish spots	Yunan province	website 22
	Hill Blue-flycatcher *Cyornis banyumas*	Nestling	22·6×16·3		4.5~5.5, 23~24×18~19	light turquoise	Yunnan province, June 2017	(Luo et al., 2018)
	Rufous-bellied Niltava Niltava sundara	Egg, Nestling, Fledging		chocolate	16~19×12.9~14.2	leather yellow	Yunnan province, July 2021	website 31

*Lesser Shortwing *Brachypteryx leucophris*	Egg	22.6×16.3	plain olive-brown becoming darke	18.5~23.0×14~15	olive-green with light reddish-brown freckles	India	(del Hoyo et al., 2020a)
*Small Niltava *Niltava macgrigoriae*	Egg	22.6×16.3	plain olive-brown becoming darke	16~19.1× 12.9~14.2	white with dark red spots	India	(del Hoyo et al., 2020a); website 33
*Buff-breasted Babbler *Pellorneum tickelli*	Egg	22.6×16.3	plain olive-brown becoming darke	19.9×15.7	light celadon with light rufous or purple grey spots	India	(del Hoyo et al., 2020a)
*Spotted Forktail *Enicurus maculatus*	Egg	22.5×15.4	uniform olive-brown to green, darker	23~26×16~18	laurel-green with reddish-brown spots	Myanmar	(del Hoyo et al., 2020a)
*Plumbeous Water-redstart *Phoenicurus fuliginosus*	Egg	22.5×15.4	uniform olive-brown to green, darker	1.9~2.4, 18.6~20.0 ×14.2×15.2	white with brown spots	Myanmar	(del Hoyo et al., 2020a)

	*Eyebrowed Wren-babbler *Napothera epilepidota*	Egg	22.5×15.4	uniform olive-brown to green, darker			Myanmar	(del Hoyo et al., 2020a)
	*Yellow-throated Fulvetta *Schoeniparus cinereus*	Egg	22.5×15.4	uniform olive-brown to green, darker	18×14	white with ochre spots	Myanmar	(del Hoyo et al., 2020a)
	*Grey-headed Parrotbill *Paradoxornis gularis*	Egg	22.5×15.4	uniform olive-brown to green, darker			Myanmar	(del Hoyo et al., 2020a)
	*Little Spiderhunter *Arachnothera longirostra*	Egg	22.5×15.4	uniform olive-brown to green, darker	17.0~19.1× 12.5~13.9	creamy with zone of heavy red-brown or purple-brown spots	Myanmar	(del Hoyo et al., 2020a)
	*Streaked Spiderhunter *Arachnothera magna*	Egg	22.5×15.4	uniform olive-brown to green, darker	20.9~24.2× 15~16.4	caesious with brown spots	Myanmar	(del Hoyo et al., 2020a)
Northern Hawk-cuckoo	*Eurasian Skylark *Alauda arvensis*	Egg	28×20	pale blue	2.5~3.0, 20~27.5× 15~17.5	offwhite with black-brown spots	Japan	(del Hoyo et al., 2020b)

Hierococcyx hyperythrus	*Japanese Thrush *Turdus cardis*	Egg	26.4×18.9	blue with light brown spots	Japan	(del Hoyo et al., 2020b)
	*Siberian Blue Robin *Larvivora cyane*	Egg	2.0~2.1, 17~21× 13~15.5	sky blue; turquoise	Japan	(del Hoyo et al., 2020b)
	*Red-flanked Bluetail *Tarsiger cyanurus*	Egg	2.0~2.5, 17.5~18× 13.0~14.5	white with reddish-brown spots	Japan	(del Hoyo et al., 2020b)
	*Asian Brown Flycatcher *Muscicapa dauurica*	Egg	16.2~17.6× 12.3~14.0	offwhite; light turquoise	Japan	(del Hoyo et al., 2020b)
	*Blue-and-white Flycatcher *Cyanoptila cyanomelana*	Egg	1.2~2.8, 16~23× 14~17	white; white with light brown spots	Japan	(del Hoyo et al., 2020b)
	*Olive-backed Pipit *Anthus hodgsoni*	Egg	1.8~2.0, 14.5~17× 20~23.3	pale blue with fuchsia spots	Japan	(del Hoyo et al., 2020b)
Banded Bay Cuckoo *Cacomantis sonneratii*	*Common Iora *Aegithina tiphia*		16.2~19×13.2~15	pale cream yellow or offwite with grey stripes	India	(Payne, 2020c)
Violet Cuckoo *Chrysococcyx xanthorhy*	*Little Spiderhunter *Arachnothera longirostra*		17.0~19.1×12.5~13.9	creamy with zone of heavy red-brown or purple-brown spots	India	(Cheke et al., 2020)

nchus	
Pied Cuckoo *Clamator jacobinus*	Unknown
Common Hawk-cuckoo *Hierococcyx varius*	Unknown

Note: The sign "*" indicates that parasitism had been recorded outside of China, but the recorded cuckoos and hosts also breed in China, suggesting that this host is a potential host for the cuckoo in the Chinese context. The sign "#" indicates that the host is newly recorded host species. Note contents: the number of eggs or nestlings of cuckoos (C) and hosts (H) in one nest.

Website source:

website 1: https://mp.weixin.qq.com/s/y3EzB_IamWVX3sgGiq15-A

website 2: https://mp.weixin.qq.com/s/MSGNJGc1hH4mEw-eqBJwSg

website 3: https://h.xinhuaxmt.com/vh512/share/11667070?d=134b2f9&channel=weixin

website 4: https://www.bilibili.com/video/BV1jM411Q7fd/

website 5: https://www.douyin.com/video/7396114869298203938

website 6: https://bbs.fengniao.com/forum/11553520.html

website 7: https://mp.weixin.qq.com/s/3vSTD7dQEvFUQpqwjWkc3A

website 8: https://www.douyin.com/video/7387764611681963274

website 9: https://channels.weixin.qq.com/platform/sphdsQRJxAZjjEJ

website 10: https://mp.weixin.qq.com/s/9ktUCcDJflljJpZAI8TF0Q

website 11: https://mbd.baidu.com/newspage/data/videolanding?nid=sv_5008765907881444423&sourceFrom=qmj

website 12: https://birdnet.cn/forum.php?mod=viewthread&tid=7853282&highlight=%B6%C5%BE%E9

website 13: https://www.bilibili.com/video/av927043270/?p=1

website 14: https://mp.weixin.qq.com/s/2WdM6x_dNPStCgcHO9UvYQ

website 15: https://www.bilibili.com/video/BV1Qu411n7XT/
website 16: https://www.kepu.net.cn/blog/public/201904/t20190401_477950.html
website 17: https://baijiahao.baidu.com/s?id=1751890423548631575&wfr=spider&for=pc
website 18: https://mp.weixin.qq.com/s/SJ1lobb9ib71d6Ko0jyKcQ
website 19: https://birdnet.cn/forum.php?mod=viewthread&tid=4761716&highlight=%C8%B5%F0%B6%2B%B6%C5%BE%E9
website 20: https://birdnet.cn/thread-7420435-1-1.html
website 21: https://www.birdnet.cn/forum.php?mod=viewthread&tid=7828760&highlight=%B6%C5%BE%E9
website 22: https://mp.weixin.qq.com/s/SJ1lobb9ib71d6Ko0jyKcQ
website 23: https://www.birdnet.cn/thread-2409798-1-1.html
website 24: https://www.birdnet.cn/thread-4056469-1-1.html
website 25: https://mp.weixin.qq.com/s/RY6QoARzLZQPkiinwUPd4w
website 26: https://www.birdnet.cn/forum.php?mod=viewthread&tid=7785944&highlight=%B4%E4%BD%F0%BE%E9
website 27: https://mp.weixin.qq.com/s/fhGZGNepXQysurPvZiMq-g
website 28: https://m.gmw.cn/2023-08/29/content_1303498415.htm
website 29: https://mp.weixin.qq.com/s/KQC9RrVprrKDa-uZt1Ynnw
website 30: https://www.bilibili.com/video/BV1qg411z7DT/
website 31: https://mp.weixin.qq.com/s/r_HL1ADnkd0Jc5vA35Ig6A
website 32: https://wap.sciencenet.cn/blog-2048045-1296278.html
website 33: https://s.weibo.com/weibo?q=%E5%A4%A7%E9%B9%B0%E9%B9%83&nodup=1&page=6
website 34: https://s.weibo.com/weibo?q=%E4%B8%AD%E6%9D%9C%E9%B9%83%E9%B8%9F%E7%B1%BB&page=46
website 35: https://mp.weixin.qq.com/s/b9fDxn-KIicOTTZSjYebVQ
website 36: https://ebird.org/checklist/S58482677?_gl=1*vbfuhu*_gcl_au*MTUzMDA4NTM3NS4xNzQyNjQxNzE4*_ga*MjExMTYzNDExMC4xNzM0ODUzNDE0*_ga_QR4NVXZ8BM*czE3NDY2MzI5ODMkbzk2JGcxJHQxNzQ2NjMzMDIwJGoyMyRsMCRoMA..&_ga=2.124103050.108023473.1746623824-2111634110.1734853414

References:

Ali S, Ripley SD (1973). ‘Handbook of the birds of India and Pakistan. Vol.8’. (Oxford Universty Press: London, UK)

Becking JH (1981). Notes on the breeding of Indian cuckoos. *Journal of the Bombay Natural History Society* **782**, 201–231.

Begum S, Moksnes A, Røskaft E, Stokke BG (2011). Interactions between the Asian koel (*Eudynamys scolopacea*) and its hosts. *Behaviour* **148**, 325–340.

Bu F, Zhang L, Ren B (1999). Breeding ecology of Indian Cuckoo in Xiaodian area of Taiyuan city, Shanxi Province. *Shanxi Forestry Science and Technology* **3**, 36–38.

Carey GJ, Chalmers ML, Diskin DA, Kennerley PR, Leader PJ, Leven MR, Lewthwaite RW, Melvilee DS, Turnbull M, Young L (2001). ‘The avifauna of Hong Kong’. (Hong Kong Bird Watching Society: Hong Kong, China)

Cheke R, Mann C, Kirwan GM (2020). Little Spiderhunter (*Arachnothera longirostra*), version 1.0. In ‘Birds of the World’. (Cornell Lab of Ornithology: Ithaca, NY, USA) Available at:

https://birdsoftheworld.org/bow/species/litspi1/cur/introduction

Cheng T (1973). ‘Avifauna of Qingling Mountain’. (Science Press: Beijing, China)

Cheng T (1963). ‘Resource fauna of China. Aves.’ (Science Press: Beijing, China)

Cheng T, Xian Y, Guan G (1991). ‘Fauna Sinica: Aves, Columbiformes, Psittaciformes, Cuculiformes and Strigiformes’. (Science Press: Beijing, China)

Clement P, Christie DA (2020). Hainan Blue Flycatcher (*Cyornis hainanus*), version 1.0. In ‘Birds of the World’. (Cornell Lab of Ornithology: Ithaca, NY, USA)

Collar N (2020). Chestnut-bellied Rock-Thrush (*Monticola rufiventris*), version 1.0. In ‘Birds of the World’. (Cornell Lab of Ornithology: Ithaca, NY, USA)

Deng W (2013). Brood parasitism on the Yellow-rumped Flycatcher (*Ficedula zanthopygia*) by the Oriental Cuckoo (*Cuculus optatus*) in an artificial nestbox in Beijing. *Chinese Birds* **4**, 187–188.

Erritzøe J, Mann CF, Brammer FP, Fuller RA (2012). ‘Cuckoos of the World’. (Christopher Helm: London, UK)

Fan L, Liu R, Song Q (2000). Breeding ecology of Large-Hawk cuckoo in Lishan Nature Reserve. *Sichuan Journal of Zoology* **19**, 85–86.

Fu T, Gao W, Song Y (1984). ‘Birds of Changbai Mountain’. (Northeastern China Normal University Press: Jilin)

Gao G, Yuan Q, Huang M, Liang D, Li B, Luo X (2024). Brood Parasitism on the Streak-Breasted Scimitar Babbler *Pomatorhinus ruficollis* by the Large Hawk Cuckoo *Hierococcyx sparverioides* Was Found in the Gaoligong Mountains, Yunnan, China. *Chinese Journal of Zoology* **59**, 981–986.

Gao W (2004). ‘The Ecology of Cavity-nesting Birds of Northeastern China’. (Jilin Science and Technology Press: Jilin)

Gao W, Yang Z, Luo W (1990). Observation on breeding behavior of three bird species. *Chinese Journal of Wildlife* **4**, 10–11.

Grim T, Samaš P, Procházka P, Rutila J (2014). Are tits really unsuitable hosts for the Common Cuckoo? *Ornis Fennica* **91**. doi:10.51812/of.133853

Guangdong Institute of Entomology, Sun Yat-sen University (1983). ‘Birds and Mammals of Hainan Island’. (Science Press: Beijing, China)

Guo L, Liu J, Liang W (2024). Brood Parasitism on Himalayan Prinia *Prinia crinigera* by Plaintive Cuckoo *Cacomantis merulinus* in Yunnan, Southwestern China. *Chinese Journal of Zoology* **59**, 815–816.

Guo W, Hu Z, Lin B, Kuang Y, Cao H, Wang C (2022). Nest site selection and breeding ecology of the red-billed blue magpie *Urocissa erythrorhyncha* in central China. *Animal Biology* **72**, 153–164.

Hao S, Wang Y (1992). Breeding ecology of the Oriental Reed Warbler. *Shandong Forestry Science and Technology* **1**, 20–22.

Higuchi H (1998). Host Use and Egg Color of Japanese Cuckoos. In ‘Parasitic Birds and Their Hosts, Studies in Coevolution’. (Eds SI Rothstein, SK Robinson.) (Oxford University Press: Oxford, United Kingdom)

del Hoyo J, Collar N, Kirwan GM (2020a). Hodgson’s Hawk-Cuckoo (*Hierococcyx nisicolor*), version 1.0. In ‘Birds of the World’. (Cornell Lab of Ornithology: Ithaca, NY, USA)

del Hoyo J, Collar N, Kirwan GM (2020b). Northern Hawk-Cuckoo (*Hierococcyx hyperythrus*), version 1.0. In ‘Birds of the World’. (Cornell Lab of Ornithology: Ithaca, NY, USA)

Hu Y, Wang X, Chang H, Sun Y (2013). Brood Parasitism on Elliot’s Laughingthrush by Large Hawk Cuckoo. *Chinese Journal of Zoology* **48**, 292–293.

Huang Q, Wang L, Yang C, Liang W (2015). Brood Parasitism on Two Tailorbird Hosts (*Orthotomus spp.*) by Plaintive Cuckoo (*Cacomantis merulinus*). *Chinese Journal of Zoology* **50**, 790–794.

Huo J, Su T, Yang C, Liang W (2014). Brood Parasitism and Egg Mimicry on *Garrulax canorus* and *Babax lanceolatus* by *Clamator coromandus*. *Sichuan Journal of Zoology* **33**, 337–341.

Huo J, Su T, Yao X, Yang C, Liang W (2016). Brood Parasitism on White-bellied Redstart (*Hodgsonius phaenicuroides*) by Large Hawk-cuckoo (*Cuculus sparverioides*). *Chinese Journal of Zoology* **51**, 1101–1105.

Jadav PCA, Parasharya BM (2014). Intensity of Brood Parasitism of Asian Koel (*Eudynamys scolopacea*) in the Nest of House Crow (*Corvus splendens*) and Jungle Crow (*Corvus macrorhynchos*) in Anand Region of Gujarat, India. *Trends in Biosciences* **7**, 4471–4476.

Jia C, Liang W, Gong H (2007). Chestnut-winged Cuckoo parasitized the Hwamei. *Chinese Journal of Zoology* **42**, 38.

Jiang A (2013). Brood parasitism on White-throated Fantail (*Rhipidura albicollis*) by Asian Emerald Cuckoo (*Chrysococcyx maculatus*) in Guangxi, southwestern China. *Journal of Hainan Normal University (Natural Science)* **26**, 198–199.

Jiang A, Zhou F, Liu N (2014). Significant recent ornithological records from the limestone area of south-west Guangxi, south China, 2004–2012. *Forktail* **30**, 122–129.

Jiang Y, Liang W, Yang C, Sun Y (2007). Large Hawk-cuckoo parasitized the White-browed Laughingthrush. *Sichuan Journal of Zoology* **26**, 509.

Kim Y (2011). A study on the breeding ecology of *Terpsiphone atrocaudata* on Jeju Island, Korea. Ph.D. thesis, Jeju National University Jeju, Korea.

La Touche JDD (1927). 'A Handbook of the Birds of Eastern China'. (Taylor and Francis: London)

Lewthwaite RW (1996). Forest Birds of Southeastern China: Observations during 1984–1996. Hong Kong Bird Report, Hong Kong.

Li G (1985). 'Fauna of Sichuan Province. Vol.3: Birds'. (Sichuan Science and Technology Press: Chengdu, China)

Liang W, Møller AP, Stokke BG, Yang C, Kovařík P, Wang H, Yao C-T, Ding P, Lu X, Moksnes A, Røskaft E, Grim T (2016). Geographic variation in egg ejection rate by great tits across 2 continents. *Behavioral Ecology* **27**, 1405–1412.

Liang W, Yang C, Takasu F (2017). How can distinct egg polymorphism be maintained in the rufescent prinia (*Prinia rufescens*)–plaintive cuckoo (*Cacomantis merulinus*) interaction—a modeling approach. *Ecology and Evolution* **7**, 5613–5620.

Limparungpatthanakij WL (2020). Asian Koel (*Eudynamys scolopaceus*), version 1.0. In 'Birds of the World'. (Cornell Lab of Ornithology: Ithaca, NY, USA)

Lin R (2008). Observation of Oriental Cuckoo parasitism on Rufous-capped Babbler in central Taiwan. *Natural Conservation Quarterly* **64**, 58–62.

Lin S, Guo S, Hao L, He L, Liang W (2024). New Host Species Recorded for Four Parasitic Cuckoo Species in China. *Chinese Journal of Zoology* **59**, 632–635.

Liu D, Xin M, Wang X (1992). Study on migration rules and feeding habits of Cuculiformes in Qingdao area. *Shandong Forestry Science and Technology* **1**, 25–26.

Liu H, Feng J, Su H (1984). Observation on egg-laying of the common cuckoo. *Sichuan Journal of Zoology* **3**, 14–16.

Liu H, Su H, Shen S, Lan Y, Ren J, Wu W (1988). Breeding ecology of the Eurasian Wren in Guandi Mountain, Shanxi Province. *Chinese Journal of Zoology* **23**, 8–12.

Liu J, Lin S, Liang W (2025). An updated list of parasitic cuckoos and their hosts in China. *Avian Research* **16**, 100249.

Liu X, Long G (1986). Breeding behavior of the Light-vented Bulbul. *Chinese Journal of Zoology* **5**, 12–15.

Liu X, Zhong G, Zhang Y, He G, Wang L, Liang W (2022). A Parasitism Case of the White Wagtail (*Motacilla alba*) Nest from Common Cuckoo (*Cuculus canorus*). *Chinese Journal of Wildlife* **43**, 725–730.

Lu X (1988). Common cuckoo parasitism on the oriental reed warbler. *Sichuan Journal of Zoology* **7**, 21–22.

Luo K, Guan S, Lu ZY, Zhao H, Li DL (2018). Brood Parasitism on Hill Blue-flycatcher (*Cyornis banyumas*) by Whistling Hawk Cuckoo (*Hierococcyx nisicolor*) in Yunnan, Southwestern China. *Chinese Journal of Zoology* **53**, 125, 142.

Ma L (2018). Comparison of counteradatations in four sympatric host species to defense against cuckoo parasitism. Ph.D. thesis, Hainan Normal University Haikou, China.

Ma W, Liu N, Ding W, Wang L, Bo X (2012). Brood Parasitism on *Lanius isabellinus* by *Cuculus canorus*. *Sichuan Journal of Zoology* **31**, 74–76.

Marchetti K (1992). Costs to host defence and the persistence of parasitic cuckoos. *Proceedings of the Royal Society of London. Series B: Biological Sciences* **248**, 41–45. doi:10.1098/rspb.1992.0040

Meshcheryagina S, Gennadiy B, Bourski O (2017). Distribution of the Oriental Cuckoo gentes in Russia: a review of brood parasitism records by the host species. *Fauna of the Urals and Siberia* **2**, 39–163.

Meshcheryagina SG, Mashanova A, Bachurin GN, Mitiay IS, Golovatin MG (2018). Host species determines egg size in Oriental cuckoo. *Journal of Zoology* **306**, 147–155.

Payne RB (2020a). Asian Emerald Cuckoo (*Chrysococcyx maculatus*), version 1.0. In 'Birds of the World'. (Cornell Lab of Ornithology: Ithaca, NY, USA)

Payne RB (2020b). Banded Bay Cuckoo (*Cacomantis sonneratii*), version 1.0. In 'Birds of the World'. (Cornell Lab of Ornithology: Ithaca, NY, USA)

Payne RB (2020c). Indian Cuckoo (*Cuculus micropterus*), version 1.0. In 'Birds of the World'. (Cornell Lab of Ornithology: Ithaca, NY, USA)

Payne RB, Kirwan GM (2020a). Large Hawk-Cuckoo (*Hierococcyx sparverioides*), version 1.0. In 'Birds of the World'. (Cornell Lab of Ornithology: Ithaca, NY, USA)

Payne RB, Kirwan GM (2020b). Oriental Cuckoo (*Cuculus optatus*), version 1.0. In 'Birds of the World'. (Cornell Lab of Ornithology: Ithaca, NY, USA)

Payne RB, Kirwan GM (2020c). Plaintive Cuckoo (*Cacomantis merulinus*), version 1.0. In 'Birds of the World'. (Cornell Lab of Ornithology: Ithaca, NY, USA)

Payne RB, Kirwan GM (2020d). Square-tailed Drongo-Cuckoo (*Surniculus lugubris*), version 1.0. In 'Birds of the World'. (Cornell Lab of Ornithology: Ithaca, NY, USA)

Qian Y, Zhang J (1965). 'Birds and Mammals of Southern Xinjiang'. (Science Press: Beijing, China)

Su T, Huo J, Yang C, Liang W (2017). Brood Parasitism on Barn Swallow (*Hirundo rustica*) Populations in China by Common Cuckoo (*Cuculus canorus*). *Chinese Journal of Zoology* **52**, 338–341.

Su T, Huo J, Yang C, Liang W (2014). Brood Parasitism on Three Host Species by Himalayan Cuckoo. *Chinese Journal of Zoology* **49**, 505–510.

Su T, Jiang A, Liang W (2016). New Host Records of Whistling Hawk Cuckoo and Drongo Cuckoo. *Chinese Journal of Zoology* **51**, 1142–1143.

Tian F, Song Y, Hao S, Feng Z, Wang Y (1991). Notes on ecology of the common cuckoo in Nansi Lake. *Shandong Forestry Science and Technology* **1**, 9–12.

Vaughan RE, Jones KH (1913). The Birds of Hong Kong, Macao, and the West River or Si Kiang in South-eastern China, with special reference to their Nidification and Seasonal Movements. *Ibis* **55**, 163–200.

Wang H, Jiang Y, Gao W (2011). Jankowski's bunting (*Emberiza jankowskii*): current status and conservation. *Chinese Birds* **1**, 251–258.

Wang J (2012). On the Ecology of Indian Cuckoo *Cuculus micropterus* in Luya National Nature Reserve, Shanxi Province. *Chinese Journal of Wildlife* **33**, 184–186.

Wang L (2015). Comparison of counter-adaptation in two sympatric prinias to defense against cuckoo parasitism. Doctoral dissertation, Wuhan University Wuhan, Chian.

Wang P, Huang X, Dong L, Zhang Z (2014). The Multiple Brood Parasitism of Eastern Crowned Warbler by Oriental Cuckoo in Xiaolongmen National Forest Park of Beijing. *Chinese Journal of Zoology* **49**, 511–515.

Wang P, Yang A, Zhang Z, Fu Y (2016). The Brood Parasitism of Pygmy Wren Babbler (*Pnoepyga pusilla*) by Lesser Cuckoo (*Cuculus poliocephalus*). *Chinese Journal of Zoology* **51**, 319–322.

Wang Y (2018). Nest Parasitism on Siberian Stonechat by Common Cuckoo in Saihanba Forest Region. *Chinese Journal of Wildlife* **39**, 699–701.

Wang Z, Jia C, Sun Y (2004). Parasitized breeding and nestlings growth in Oriental Cucko. *Chinese Journal of Zoology* **39**, 103–105.

Well DR (1999). 'The Birds of the Thai-Malay Peninsula. Vol. 1, Non-passerines'. (Academic Press: New York, USA)

Yan A (1985). Observation on Indian Cuckoo. *Chinese Biological Bulletin* **3**, 13.

Yang C, Antonov A, Cai Y, Stokke BG, Moksnes A, Røskaft Ei, Liang W (2012a). Large Hawk-Cuckoo *Hierococcyx sparverioides* parasitism on the Chinese Babax *Babax lanceolatus* may be an evolutionarily recent host–parasite system. *Ibis* **154**, 200–204.

Yang C, Cai Y, Liang W (2008). Asian Emerald Cuckoo parasitized the Bianchi's Warbler *Seicercus valentini*. *Chinese Journal of Zoology* **43**, 74–75.

Yang C, Cai Y, Liang W (2010a). Brood parasitism and egg mimicry on Brownish-flanked Bush Warbler (*Cettia fortipes*) by Lesser Cuckoo (*Cuculus poliocephalus*). *Zoological Research* **31**, 555−560.

Yang C, Cai Y, Liang W (2011). Visual modeling reveals cryptic aspect in egg mimicry of Himalayan Cuckoo (*Cuculus saturatus*) on its host Blyth's Leaf Warbler (*Phylloscopus reguloides*). *Zoological Research* **32**, 451–455.

Yang C, Lan H, Yao X, Yu D (2023). 'Cuckoos and Their Hosts in Kuankuoshui National Nature Reserve of Guizhou'. (China Forestry Publishing House: Beijing, China)

Yang C, Liang W, Antonov A, Cai Y, Stokke BG, Fossøy F, Moksnes A, Røskaft E (2012b). Diversity of parasitic cuckoos and their hosts in China. *Chinese Birds* **3**, 9–32.

Yang C, Liang W, Cai Y, Shi S, Takasu F, Møller AP, Antonov A, Fossøy F, Moksnes A, Røskaft E, Stokke BG (2010b). Coevolution in Action: Disruptive Selection on Egg Colour in an Avian Brood Parasite and Its Host. *PLoS ONE* **5**, e10816.

Yang C, Su T, Liang W, Møller AP (2015). Coevolution between the large hawk-cuckoo (*Cuculus sparverioides*) and its two sympatric Leiothrichidae hosts: evidence for recent expansion and switch in host use? *Biological Journal of the Linnean Society* **115**, 919–926.

Yi T (2020). Coevolution between large hawk cuckoos and its hosts. A Dissertation Submitted for the Degree of Ph.D, Hainan Normal University Haikou, China.

Zhang J (2001). Observation on the breeding habits of *Dicrurus macrocercus*. *Chinese Journal of Zoology* **36**, 60–63.

Zhang S, Zhang S, Yan F (2017). Investigation on the parasitic breeding habit of *Cuculus micropterus* and *Cuculus canorus bakeri*. *Shandong Forestry Science and Technology* **47**, 68–69.

Zhang T (1989). Studies on breeding ecology of the common cuckoo. *Shandong Forestry Science and Technology* **1**, 24–26.

Zhang W (1980). 'A Field Guide to the Birds of Taiwan'. (Insititue of Environmental Science, Tunghai University: Taiwan, China)

Zhang Y, Zhong G, He G, Liu X, Wang L, Liang W (2021). One nest of Buff-throated Warbler was parasitized by Asian Emerald Cuckoo in Guizhou. *Sichuan Journal of Zoology* **40**, 446–447.

Zhang Y, Zhong G, Wan G, Wang L, Liang W (2023). Brood parasitism and egg recognition in three bunting hosts of the cuckoos. *Ecology and Evolution* **13**, e10659.

Zhao Z (1985). 'The Avifauna of Changbai Mountain'. (Jilin Science and Technology Press: Changchun, China)

Zhao Z, He J (1981). Studies on the breeding biology of blue-and-white flycatcher. *Acta zoologica sinica* **27**, 388–394.

Supplementary Information 2 The phylogenetic tree of host species

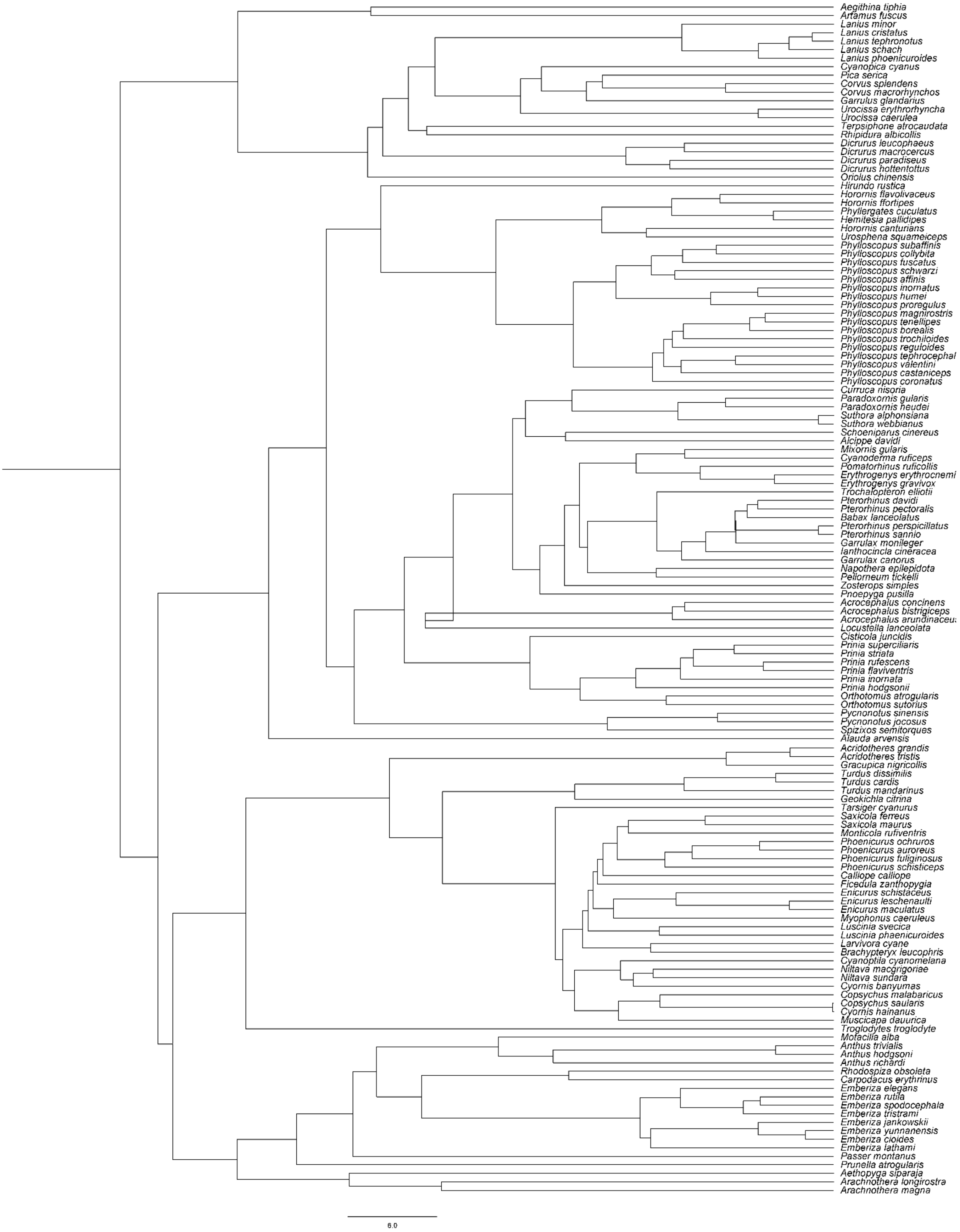

Supplementary Information 3 The real-time tracking program for parasitism records

To access and use the program, scan the QR code above with the WeChat app.
