# Supplementary material for: Host Exploitation by Cuckoos in China: A Review and Real‐Time Tracking Program for Parasitism Records
Source: Integr Zool. 2025 Jul 17;21(2):218–30. doi: 10.1111/1749-4877.13009 (PMC12971627; doi:10.1111/1749-4877.13009)
Supplement: Supplementary file 2 — SUPPLEMENTARY MATERIAL: inz213009‐sup‐0002‐SuppMat.docx [file INZ2-21-218-s002.docx]

**SUPPLEMENTARY MATERIAL**

**Supplementary Information 1**

Appendix: Parasitic cuckoo species and their hosts in China

| **Cuckoo name** | **Host name** | **Cuckoo state** | **Note contents** | **Cuckoo egg mass (g) and size (mm)** | **Cuckoo egg morph** | **Host egg mass (g) and size (mm)** | **Host egg morph** | **Site and Year** | **Source** |
| --- | --- | --- | --- | --- | --- | --- | --- | --- | --- |
| Chestnut-winged Cuckoo *Clamator coromandus* | Hwamei *Garrulax canorus* | Nestling | 1C+3H |  |  |  |  | Guizhou province, May 2005 | (Jia et al., 2007) |
|  |  | Nestling | 1C+3H |  |  |  |  | Guizhou province, May 2005 | (Jia et al., 2007) |
|  |  | Egg |  |  | blue |  | blue |  | (La Touche, 1927) |
|  |  | Egg | 4C+2H | 7.49 ± 0. 14, 26.82 ± 0.73 × 22.50 ± 0. 69 | turquoise, blue | 5.62 ± 0.74, 26.16 ± 1.03 × 20.50 ± 0.80 | light turquoise | Guizhou province, 2013 | (Huo et al., 2014) |
|  |  | Fledgling |  |  |  |  |  | Xiamen, 2021 | website 1 |
|  |  | Fledgling |  |  |  |  |  | Xiamen, 2022 | website 2 |
|  | Masked Laughingthrush *Pterorhinus perspicillatus* | Egg |  |  | blue | 27~28×19~21 | Dusty blue; Light cyan; Light green with auburn spots | Eastern China | (La Touche, 1927) |
|  | Greater Necklaced Laughingthrush *Pterorhinus pectoralis* | Nestling | 1C |  |  |  |  | Hong Kong, May 2005 | (Carey et al., 2001) |
|  |  | Nestling | 1C |  |  |  |  | Hong Kong, July 1995 | (Carey et al., 2001) |
|  |  | Nestling | 1C |  |  |  |  | Hong Kong, August 1996 | (Carey et al., 2001) |
|  |  | Nestling | 1C |  |  |  |  | Hong Kong, May 1998 | (Carey et al., 2001) |
|  |  | Egg | 2C+6H | 25.4~29.9×20.3~24.4 | blue | 28.7~33.8×20.9~24.1 | blue | Hubei province, June 2006 | (Yang et al., 2012b) |
|  |  | Fledgling |  |  |  |  |  | Yunnan province, 2023 | website 3 |
|  | Oriental Magpie Robin *Copsychus saularis* | Egg |  | 25.4~29.9×20.3~24.4 | blue | 20.4~23.0×16.1~17.4 | Reseda with dark brown spots | Guangdong province | (Guangdong Institute of Entomology, Sun Yat-sen University, 1983) |
|  | Chinese Babax *Babax lanceolatus* | Egg | 2C+2H | 7.49 ± 0. 14, 26.82 ± 0.73 × 22.50 ± 0. 69 | turquoise | 5.92 ± 0. 62, 27.53 ± 1.03 × 20.53 ± 0.61 | deep turquoise | Guizhou province, 2013 | (Huo et al., 2014) |
|  | *Slaty-backed Forktail *Enicurus schistaceus* |  |  | 25.4–29·9×20.3–24.4 |  | 2.6~2.7, 20.6~22.0×16.2~16.8 | White with brown spots | Mainland Southeast Asia | (Erritzøe et al., 2012) |
|  | *Lesser Necklaced Laughingthrush *Garrulax monileger* |  |  | 25.4–29·9×20.3–24.4 |  | 28.4×21.3 | Dark turquoise | Mainland Southeast Asia | (Erritzøe et al., 2012) |
|  | *Orange-headed Thrush *Geokichla citrina* |  |  | 25.4–29·9×20.3–24.4 |  | 21~28×17~21.6 |  | Mainland Southeast Asia | (Erritzøe et al., 2012) |
|  | *Long-tailed Shrike *Lanius schach* |  |  | 25.4–29·9×20.3–24.4 |  | 6.5~8.1, 22.4~23.7×27.2~30.5 |  | Mainland Southeast Asia | (Erritzøe et al., 2012) |
|  | *Blue Whistling-thrush *Myophonus caeruleus* |  |  | 25.4–29·9×20.3–24.4 |  | 11.3~12.6, 34.9~37.2×24.7~25.6 |  | Mainland Southeast Asia | (Erritzøe et al., 2012) |
|  | *Black-breasted Thrush *Turdus dissimilis* |  |  | 25.4–29·9×20.3–24.4 |  | 21.1~29×18.3~21 |  | Mainland Southeast Asia | (Erritzøe et al., 2012) |
| Large Hawk-Cuckoo *Hierococcyx sparverioides* | Chinese Babax *Babax lanceolatus* | Egg | 1C+2H | 6.6, 27.1 × 21.4 | white | 6.0, 27.8 × 20.6 | deep sky blue | Guizhou province, May 2008 | (Yang et al., 2012a) |
|  |  | Egg | 1C+2H |  | white |  | deep sky blue | Guizhou province, June 1999 | (Yang et al., 2012b) |
|  |  | Egg | 1C+1H |  | white |  | deep sky blue | Guizhou province, July 1999 | (Yang et al., 2012b) |
|  |  | Egg | 1C+1H |  | white |  | deep sky blue | Guizhou province, July 1999 | (Yang et al., 2012b) |
|  |  | Egg | 1C+2H |  | white |  | deep sky blue | Guizhou province, August 1999 | (Yang et al., 2012b) |
|  |  | Egg |  |  |  |  |  | Guizhou province | (Yang et al., 2015) |
|  | White-browed Laughingthrush *Pterorhinus sannio* | Egg | 1C+4H | 6.2, 27.0 × 20.6 | white | 5.7, 27.8 × 19.7 | white | Guizhou province, May 2006 | (Jiang et al., 2007) |
|  |  | Egg | 1C+2H | 7.5, 30.4 × 21.8 | white | 5.3, 26.1 × 20.0 | white | Guizhou province, May 2008 | (Yang et al., 2012b) |
|  |  | Egg | 1C+1H |  | white |  | blue | Guizhou province July 1999 | (Yang et al., 2012b) |
|  |  | Egg | 1C+1H |  | white |  | blue | Guizhou province July 1999 | (Yang et al., 2015, 2012b) |
|  |  | Fledging |  |  |  |  |  |  | website 4 |
|  | Moustached Laughingthrush *Ianthocincla cineracea* |  |  |  |  | 25~28.5×17.5~20 | blue or plain greenish turquoise | Yunnan province | (Liu et al., 2025) |
|  | Black-necklaced Scimitar-babbler *Erythrogenys erythrocnemis* |  |  |  |  |  |  | Taiwan province | (Payne and Kirwan, 2020a) |
|  | White-bellied Redstart *Luscinia phaenicuroides* | Egg | 1C+2H | 6.09, 20.20×27.73 | white | 2.34, 20.05×14.94; 2.40, 19.95×15.09 | blue | Guizhou province, 2015 | (Huo et al., 2016) |
|  | Elliot’s Laughingthrush *Trochalopteron elliotii* | Egg |  | 4.5, 25.0×19.0; 5.0, 27.0×19.5 | offwhite with brown spots | 5.8, 28.2×20.1 | light sky blue with brown spots and steaks | Shanxi province, May 1996 | (Fan et al., 2000; Hu et al., 2013; Yi, 2020) |
|  |  | Egg |  | 6.9, 29.76×20.40; 7.2, 28.40×21.68 | turquoise | 5.8, 28.2×20.1 | light sky blue with brown spots | Gansu province | (Hu et al., 2013) |
|  |  | Fledging |  |  |  |  |  | Gansu province | (Hu et al., 2013) |
|  | Masked Laughingthrush *Pterorhinus perspicillatus* | Egg |  | 4.5, 25.0×19.0; 5.0, 27.0×19.5 | offwhite with brown spots | 5.6, 29.0×21.0 | light cyan | Shanxi province, May 1996 | (Fan et al., 2000) |
|  |  | Fledging |  |  |  |  |  |  | website 5 |
|  |  | Fledging |  |  |  |  |  |  | website 6 |
|  | Spot-breasted Scimitar Babbler *Erythrogenys gravivox* | Egg |  | 4.5, 25.0 ×19.0; 5.0, 27.0 × 19.5 | offwhite with brown spots | 6.5, 28.3×20.7 | white | Shanxi province, May 1996 | (Fan et al., 2000) |
|  | Streak-breasted Scimitar Babbler *Pomatorhinus ruficollis* | Fledging |  | 5.0, 27.0 × 19.5 |  | 4~4.5; 24~25×17~18 | white | Yunnan province, May 2022 | (Gao et al., 2024) |
|  | Hwamei *Garrulax canorus* | Nestling | 1C |  |  |  |  | Guangxi province, May 2003 | (Yang et al., 2012b) |
|  |  | Egg | 1C+2H | 5.0, 27.0 × 19.5 | white | 3.6~6.3, 24.0~28.4×18.8~21.8 | blue | Guizhou province, June 1999 | (Yang et al., 2012b) |
|  | Oriental Magpie  *Pica serica* | Egg | 2C+4H | 4.7, 26.2 × 19.2 | olive grey with brown spots | 9~13, 23~26×32~38 | light turquoise with brown or black spots | Beijing, June 1973 | (Cheng et al., 1991) |
|  | *Little Spiderhunter *Arachnothera longirostra* |  |  |  |  | 17.0~19.1×12.5~13.9 | dull white or creamy with zone of heavy red-brown or purple-brown spots | India | (Payne and Kirwan, 2020a) |
|  | *Streaked Spiderhunter *Arachnothera magna* |  |  |  |  | 20.9~24.2×15~16.4 | caesious with brown spots | India | (Payne and Kirwan, 2020a) |
|  | *Lesser Shortwing *Brachypteryx leucophris* |  |  |  |  | 18.5~23.0×14~15 | olive-green with light reddish-brown freckles | India | (Payne and Kirwan, 2020a) |
|  | *Hainan Blue Flycatcher *Cyornis hainanus* |  |  |  |  |  |  | Mainland Southeast Asia | (Clement and Christie, 2020) |
| Indian Cuckoo *Cuculus micropterus* | Azure-winged Magpie *Cyanopica cyanus* | Nestling |  |  |  |  |  | Jiangsu province, 1974 | (Yan, 1985) |
|  |  | Egg |  | 4.0, 21.0×17.5; 24.0×18.0 |  | 6.0, 28.1×20.2 | lightcyan with brown and purple spots | Shanxi province, June 1996 | (Bu et al., 1999) |
|  |  | Fledgling |  |  |  |  |  | Shandong province, 2009 | (Yang et al., 2012b) |
|  |  | Egg |  |  |  |  |  | Shandong province | (Zhang et al., 2017) |
|  |  | Nestling |  |  |  |  |  | Shandong province | (Zhang et al., 2017) |
|  |  | Fledgling |  |  |  |  |  | Shandong province | (Zhang et al., 2017) |
|  | Ashy Woodswallow  *Artamus fuscus* |  |  |  |  | 22.0~23.4×16.7~17.1 | offwhite or pale greenish-white, covered with spots of varying sizes ranging from brownish-yellow to tan, some also interspersed with sparse gray,  reddish-brown, and pale purple speckles | Guangdong province | (Liu et al., 2025) |
|  |  |  |  |  |  | 22.0~23.4×16.7~17.1 | offwhite or pale greenish-white, covered with spots of varying sizes ranging from brownish-yellow to tan, some also interspersed with sparse gray,  reddish-brown, and pale purple speckles | Guangxi province | (Liu et al., 2025) |
|  | Meadow Bunting *Emberiza cioides* | Egg |  |  |  | 1.8~2.0, 19~21×15~17 | white with brown linear, wavy, or tadpole-like stripes and spots | Shandong province | (Liu et al., 1992) |
|  | White-crowned Forktail *Enicurus leschenaulti* |  |  |  |  | 3.61±0.17, 23.55×17.61 | white with brown spots | Hainan province | (Liu et al., 2025) |
|  | Plain Laughingthrush *Pterorhinus davidi* | Egg | 2C | 3.4, 23~18×22~17 |  | 25~27×19~21 | plain turquoise-blue | Shanxi province | (Wang, 2012) |
|  | Oriental Reed Warbler  *Acrocephalus orientalis* | Egg |  | 4.0, 21.0×17.5; 24.0×18.0 |  | 3.8, 22.4×16.0 | offwhite with olive spots | Shanxi province, June 1996 | (Bu et al., 1999; Hao and Wang, 1992) |
|  |  | Nestling |  |  |  |  |  | Jiangsu province, 1980 | (Hao and Wang, 1992; Yan, 1985) |
|  | Light-vented Bulbul *Pycnonotus sinensis* | Nestling |  | 5.4, 25.25×20.1 |  | 2.6~3.3, 21.5~24×16~16.6 | pink with purple spots | Jiangsu province, 1983 | (Liu and Long, 1986; Yan, 1985) |
|  | Black Drongo *Dicrurus macrocercus* | Egg | 1C+2H |  | pinkish white with firebrick spots | 4.8, 24.7×18.6 | lightpink with brown spots | Hong Kong | (Vaughan and Jones, 1913; Zhang, 2001) |
|  |  | Fledgling |  |  |  |  |  | Guangxi province, July 2019 | website 7 |
|  |  | Fledgling |  |  |  |  |  | Shandong province, July 2024 | website 8 |
|  | Chinese Blackbird *Turdus mandarinus* | Egg | 1C+2H | 6.8, 29.5×22.7 | offwhite with firebrick spots | 7.2, 29.1×21.4 | lightcyan with brown spots | Anhui province, April 1996 | (Zhang, 2001) |
|  | Siberian Stonechat *Saxicola torquata* | Nestling |  |  |  | 1.5~2.1, 16~19.8×12~15.6 | turquoise with reddish-brown spots | Yangtze Basin | (Cheng et al., 1991) |
|  | Black-naped Oriole Oriolus chinensis | Fledgling |  |  |  |  |  | Guangdong province, 2023 | (Liu et al. 2025); website 9 |
|  | Brown Shrike *Lanius cristatus* | Fledgling |  |  |  | 3.1~3.5, 15~19×21.1~24.5 | White with fulvous spots; Grey with fulvous spots | Jilin province, 2023 | (Payne, 2020a); website 10 |
|  | *Grey Drongo *Dicrurus leucophaeus* |  |  |  |  | 18~20×23~26 | light pink with grey, dark red, claybank, and black brown spots | India | (Payne, 2020a) |
|  | *Greater Racquet-tailed Drongo *Dicrurus paradiseus* |  |  |  |  | 26~32.4×20~22.5 | white; white with light red spots | Indonesia | (Payne, 2020a) |
| Common Cuckoo *Cuculus canorus* | Ashy-throated Parrotbill *Suthora alphonsiana* | Egg | 1C+4H | 2.6, 20.8×16.2 | turquoise | 1.3, 16.1×12.7 | turquoise | Guizhou province, June 2008 | (Yang et al., 2010b) |
|  |  | Nestling | 1C |  |  |  |  | Guizhou province, June 2008 | (Yang et al., 2012b) |
|  |  | Nestling | 1C |  |  |  |  | Guizhou province, July 2008 | (Yang et al., 2012b) |
|  |  | Egg | 1C+2H |  | blue, pale blue, white |  | blue, pale blue, white | Guizhou province, June | (Yang et al., 2010b) |
|  |  | Egg | 1C+3H |  | turquoise |  | turquoise | Guizhou province, June 1999 | (Yang et al., 2010b) |
|  |  | Nestling | 1C |  |  |  |  | Guizhou province, July 1999 | (Yang et al., 2012b) |
|  |  | Nestling | 1C |  |  |  |  | Guizhou province, August 2006 | (Yang et al., 2012b) |
|  |  | Nestling | 1C |  |  |  |  | Guizhou province, June 2005 | (Yang et al., 2012b) |
|  |  | Nestling | 1C |  |  |  |  | Guizhou province, June 2007 | (Yang et al., 2012b) |
|  |  | Egg | 1C | 3.0, 21.4×16.2 | pale blue |  |  | Guizhou province, June 2011 | (Yang et al., 2012b) |
|  |  | Egg | 1C+2H | 3.1, 21.6×16.2 | white | 1.2, 16.7×12.4 | paleblue | Guizhou province, June 2011 | (Yang et al., 2012b) |
|  | Red-billed Blue Magpie *Urocissa erythrorhyncha* |  |  |  |  | 5.6~9.2, 28.1~32.5×21.2~23.2 | earthy yellow with reddish-brown spots | Henan province | (Guo et al., 2022) |
|  | Yellow-throated Bunting *Emberiza elegans* | Egg | 1C+1H | 3.21, 22.32×17.32 | light turquoise | 2.08±0.18, 19.15±0.87×15.01±0.29 | offwhite with black spots | Guizhou province | (Zhang et al., 2023) |
|  | Crested Bunting *Emberiza  lathami* |  |  |  |  | 17.9~22.0×13.0~17.0 | offwhite with reddish brown spots | Yunnan province | (Liu et al. 2025) |
|  | Southern Rock Bunting *Emberiza yunnanensis* | Egg | 1C+1H | 3.26, 22.35×17.78 | light turquoise | 2.61±0.25, 20.58±0.83×15.62±0.50 | offwhite with reddish brown spots and lines | Guizhou province | (Zhang et al., 2023) |
|  | Swinhoe's White-eye *Zosterops simples* |  |  |  |  | 1.09±0.09, 15.93±0.55×11.89±0.46 | white | Yunnan province | (Liu et al. 2025) |
|  | Siberian Rubythroat *Calliope calliope* |  |  |  |  | 2.0~2.2, 19.0~20.5×15.0~16.5 | turquoise | Qinghai province | (Liu et al. 2025) |
|  | Great Reed Warbler *Acrocephalus arundinaceus* |  |  |  |  | 21~25×15~17 | turquoise with brown spots, some with small charcoal grey or grey spots | Xinjiang province | (Liu et al. 2025) |
|  | White-throated Redstart *Phoenicurus schisticeps* |  |  |  |  | 19~20×15~16 | pink with brown spots | Xizang province | (Liu et al. 2025) |
|  | Red-tailed Shrike *Lanius phoenicuroides* | Egg | 1C+2H |  | white with brown spots | 21~24×15.6~17.8 | pink with brown spots | Xinjiang province, 2022 | website 34 |
|  | Vinous-throated Parrotbill *Suthora webbianus* | Fledgling |  |  |  |  |  | Sichuan province | (Yang et al., 2012b) |
|  |  | Egg |  |  | turquoise with violet spots | 1.5~2, 15~19×12~13.4 | white; light blue; turquoise; pastel green | Shaanxi province | (Cheng, 1973, 1963) |
|  |  | Fledgling |  |  |  |  |  | Shandong province, 2023 | website 11 |
|  | Reed Parrotbill *Paradoxornis heudei* | Egg | 1C+2H |  | offwhite with olive spots | 1.6~2.1, 18.0~19.0×14.0~14.8 | offwhite with olive spots | Shandong province, June 2008 | (Yang et al., 2012b) |
|  |  | Fledgling |  |  |  |  |  | Shandong province, 2023 | website 12 |
|  | Daurian Redstart *Phoenicurus auroreus* | Nestling | 1C |  |  |  |  | Guizhou province, June 2005 | (Yang et al., 2012b) |
|  |  | Egg | 1C+4H | 2.8, 20.8 × 16.3 | white with grey spots | 1.7, 17.9 × 13.7 | pale turquoise with firebrick spots | Guizhou province, July 1999 | (Yang et al., 2012b) |
|  |  | Egg | 1C+5H | 2.8, 20.8 × 16.3 | white with grey spots | 1.7, 17.9 × 13.7 | white with firebrick spots | Guizhou province, June 1992 | (Yang et al., 2012b) |
|  |  | Egg | 1C+3H |  |  |  |  | Guizhou province, June 1992 | (Yang et al., 2012b) |
|  |  | Nestling | 1C |  |  |  |  | Guizhou, province June 1992 | (Yang et al., 2012b) |
|  |  | Egg | 1C+3H |  | white with grey spots |  | white with firebrick spots | Guizhou province, July 1994 | (Yang et al., 2012b) |
|  |  | Nestling | 1C |  |  |  |  | Guizhou province, May 2008 | (Yang et al., 2012b) |
|  |  | Nestling | 1C |  |  |  |  | Shaanxi province | (Cheng, 1973, 1963) |
|  |  | Fledgling |  |  |  |  |  | Shaanxi province, 2020 | website 13 |
|  | Black Redstart  *Phoenicurus ochruros* | Nestling | 1C |  |  | 18~21×13~15.1 | light turquoise; light turquoise with black-brown spots | Qinghai province, July 2008 | (Yang et al., 2012b) |
|  |  | Fledgling |  |  |  |  |  | Gansu province, August 2005 | (Yang et al., 2012b) |
|  |  | Fledgling |  |  |  |  |  | Xizang province, 2020 | website 14 |
|  | White-bellied Redstart *Luscinia phaenicuroides* | Egg | 1C+3H | 3.8, 25.9×17.9 | dark turquoise | 2.0~3.2, 18.6~23.6×15.0~16.9 | dark turquoise | Xizang province, 1999 | (Yang et al., 2012b) |
|  |  | Egg | 1C+3H | 3.8, 25.1×18.0 | dark turquoise | 2.0~3.2, 18.6~23.6×15.0~16.9 | dark turquoise | Xizang province, 1999 | (Yang et al., 2012b) |
|  |  | Egg |  | 3.76±0.16, 22.19±0.52×17.62±0.47 | light blue; dark turquoise | 2.87±0.23, 21.66± 1.03×15.97±0.44 | dark turquoise | Gansu province, 2009~2012 | (Hu et al., 2013) |
|  | Plumbeous Water Redstart *Phoenicurus fuliginosus* | Nestling | 1C |  |  | 1.9~2.4, 18.6~20.0×14.2×15.2 | white with brown spots | Jiangsu province | (Yang et al., 2012b) |
|  |  | Fledgling |  |  |  |  |  |  | website 15 |
|  |  | Fledgling |  |  |  |  |  | Shannxi province, 2013 | website 16 |
|  | Bluethroat *Luscinia svecica* | Nestling |  |  |  | 17~21×13~15 | pale green with brown spots | Xinjiang province | (Qian and Zhang, 1965) |
|  | Siberian Stonechat *Saxicola maurus* | Fledgling |  |  |  |  |  | Guizhou province, July 2007 | (Yang et al., 2012b) |
|  |  | Egg |  | 3.83, 23.18×17.38 | light taupe with dark brown spots | 1.81, 17.48×13.80 | turquoise with orange-brown spots and light brown striolae | Hebei province, 2012~2015 | (Wang, 2018) |
|  |  | Nestling |  |  |  |  |  | Hebei province, 2012~2015 | (Wang, 2018) |
|  | Grey Bushchat  *Saxicola ferreus* | Egg | 1C+3H | 2.7, 20.1×16.0 | dark turquoise | 1.9, 17.6×14.4 | medium turquoise | Guizhou province, May 2007 | (Yang et al., 2012b) |
|  |  | Egg | 1C+4H |  |  |  |  | Guizhou province, August 1999 | (Yang et al., 2012b) |
|  | White Wagtail *Motacilla alba* | Fledgling |  |  |  |  |  | Guizhou province, July 2006 | (Yang et al., 2012b) |
|  |  | Nestling | 1C |  |  |  |  | Sichuan province | (Li, 1985) |
|  |  | Egg | 1C+2H | 3.89, 23.40×17.46 | white with light brown spots | 2.21±0.21, 20.46±0.52×15.19±0.44 | white with brown spots | Guizhou province, 2020 | (Liu et al., 2022) |
|  | Richard’s Pipit *Anthus richardi* | Fledgling |  |  |  |  |  | Qinghai province, 2007 | (Yang et al., 2012b) |
|  | Oriental Reed Warbler *Acrocephalus orientalis* | Nestling |  |  |  |  |  | Shanxi province, June 1985 | (Hao and Wang, 1992; Lu, 1988) |
|  |  | Fledgling |  |  |  |  |  | Heilongjiang province, July 2007 | (Yang et al., 2012b) |
|  |  | Fledgling |  |  |  |  |  | Liaoning province | (Yang et al., 2012b) |
|  |  | Egg |  | 4.4, 24.0×18.0 | offwhite with brown spots | 3.8, 22.4×16.0 | offwhite with olive spots | Shandong province, 1984-1986 | (Tian et al., 1991) |
|  |  | Fledgling |  |  |  |  |  | Beijing province, 2011 | (Yang et al., 2012b) |
|  | Blunt-winged Warbler *Acrocephalus concinens* | Egg |  | 21.8× 16.22 | offwhite with brown spots | 15.8~18×11.8~12.8 | Light green with tawny spots | Hebei province, 2016-2017 | (Ma, 2018) |
|  | Black-browed Reed Warbler  *Acrocephalus bistrigiceps* | Egg | 1C+4H | 3.6, 25.0×17.3 | lightcyan with brown spots | 1.3, 13.8×10.7 | offwhite with brown spots | Shanxi province, June 1981 | (Liu et al., 1984) |
|  | Pale-legged Leaf Warbler *Phylloscopus tenellipes* | Egg |  | 1.8, 21.5 × 13.3 | pinkish white with sandy brown spots | 1.5, 15.6 × 12.3 | pinkish white | Jilin province, June 1979 | (Gao, 2004) |
|  |  | Nestling |  |  |  |  |  |  | (Fu et al., 1984) |
|  | Zitting Cisticola *Cisticola juncidis* | Egg |  |  | turquoise with violet spots | 13.8~16.8×10.2~12.3 | white with rufous spots; light blue with rufous spots | North China | (Cheng et al., 1991) |
|  |  | Fledgling |  |  |  |  |  |  | website 17 |
|  | Isabelline Shrike  *Lanius isabellinus* | Fledgling |  |  |  |  |  | Xinjiang province | (Yang et al., 2012b) |
|  |  | Fledgling |  |  |  |  |  | Xinjiang province | (Yang et al., 2012b) |
|  |  | Fledgling |  |  |  |  |  | Shanxi province, 2011 | (Yang et al., 2012b) |
|  |  | Egg | 1C+5H | 24.11~25.78×16.27~17.76 | White | 21.04±0.31×16.83±0.18 | White with brown spots; White with light brown spots; Pink with reddish brown spots | Gansu province, 2010 | (Ma et al., 2012) |
|  |  | Nestling | 1C |  |  |  |  | Gansu province, 2010 | (Ma et al., 2012) |
|  |  | Fledgling |  |  |  |  |  | Gansu province, 2010 | (Ma et al., 2012) |
|  | Brown Shrike  *Lanius cristatus* | Eggs |  |  |  | 3.1~3.5, 15~19×21.1~24.5 | white with fulvous spots; grey with fulvous spots | Xinjiang province | (Qian and Zhang, 1965) |
|  |  | Fledgling |  |  |  |  |  | Beijing, 2011 | (Yang et al., 2012b) |
|  |  | Fledgling |  |  |  |  |  | Henan province, 2011 | (Yang et al., 2012b) |
|  | Grey-backed Shrike *Lanius tephronotus* | Fledgling |  |  |  | 22.7~27.0×18.0~19.3 | offwhite with brownness and purple spots | Chongqing, 2009 | (Yang et al., 2012b) |
|  | Lesser Grey Shrike *Lanius minor* | Fledgling |  |  |  | 23~28×16~20 | light yellow or light green with light brown or purplish-brown spots | Xinjiang province, 2021 | website 18 |
|  | Blue-and-white Flycatcher *Cyanoptila cyanomelana* | Nestling |  |  |  | 1.2~2.8, 16~23×14~17 | white; white with light brown spots | Jilin province | (Fu et al., 1984) |
|  | Black-faced Bunting *Emberiza spodocephala* | Fledgling |  |  |  | 1.3~2.1, 18~21×13~16 | offwhite with reddish-brown spots; white with chestnut brown spots; light blue with red spots | Qinghai province, 2007 | (Yang et al., 2012b) |
|  |  | Nestling |  |  |  |  |  |  | (Fu et al., 1984) |
|  | Jankowski's Bunting *Emberiza jankowskii* | Fledgling |  |  |  |  |  | Heilongjiang province, 2005 | (Yang et al., 2012b) |
|  |  | Egg | 1C+5H |  |  | 2.0~2.5, 19.5~21×15~18 | offwhite with brown spots | Jilin province, 2011 | (Wang et al., 2011) |
|  | Azure-winged Magpie  *Cyanopica cyanus* | Egg | 1C+4H | 5.4, 26.0×20.0 | lightcyan with brown spots | 5.7, 27.4×19.3 | lightcyan with brown and purple spots | Shandong province, April 1979 | (Zhang, 1989) |
|  | Desert Finch *Rhodospiza obsoleta* | Nestling |  |  |  | 17.4~22.0×12.0~15.1 | light blue with purplish black spots; white; turquoise | Xinjiang province | (Qian and Zhang, 1965) |
|  | Barn Swallow *Hirundo rustica* | Fledgling |  |  |  | 1.3~2.5, 13~16×18~20 | White with reddish-brown spots | Gansu province, 2007; Hebei province, 2007, 2010; Jilin province, 2011; Shanxi; Shandong; Heilongjiang province | (Su et al., 2017) |
|  | Barred Warbler *Curruca nisoria* | Fledgling |  |  |  | 18~22.6×14.5~16.3 | white with grey spots; white with lavender spots | Xinjiang province, 2021 | website 32; (Yang et al., 2023) |
|  | Oriental Magpie Robin Copsychus saularis | Fledgling |  |  |  |  |  |  | website 19 |
|  |  | Fledgling |  |  |  | 20.4~23.0×16.1~17.4 | Reseda with dark brown spots | Jiangxi and Hubei province, August 2022 | (Lin et al., 2024) |
|  |  | Fledgling |  |  |  |  |  |  | website 20 |
|  | Meadow Bunting Emberiza cioides | Fledgling |  |  |  | 1.8~2.0, 19~21×15~17 | white with brown spots | Shandong province, 2023 | website 21 |
|  | Tree Sparrow Passer montanus | Fledgling |  |  |  | 2~2.6, 17.1~21.5×12.6~15.4 | offwhite with purplish brown spots | Yunnan province, 2021 | website 22 |
|  |  |  |  |  |  |  |  |  | (Yang et al., 2023) |
|  | Eurasian Wren Troglodytes troglodyte | Nestling |  |  |  | 1~1.4, 16.4~18.1×10.3~13.0 | pink with brown spots; white with reddish-brown spots | Yunnan province, 2021 | website 22 |
|  | Chestnut-bellied Rock-Thrush *Monticola rufiventris* | Fledgling |  |  |  |  | creamy-white with reddish-brown speckles | Yunnan province, 2024 | website 35 |
|  | Buff-throated Warbler Phylloscopus subaffinis | Fledgling |  |  |  | 0.7~1.2, 13.6~15.7×10.9~12.8 | white | Chongqing province, 2017 | website 23 |
|  |  | Fledgling |  |  |  |  |  |  | website 24 |
|  | *Little Spiderhunter *Arachnothera longirostra* |  |  |  |  | 17.0~19.1×12.5~13.9 | creamy with red-brown or purple-brown spots | Thailand | (Cheke et al., 2020) |
| Himalayan Cuckoo *Cuculus saturatus* | Blyth's Leaf Warbler *Phylloscopus reguloides* | Egg |  | 21.5×14.0 | white with a few brown spots | 1.0, 14.9×12.2 | white | Fujian province, 1931 | (La Touche, 1927) |
|  |  | Egg | 1C+3H | 2.1, 20.6×13.5 | white with a few brown spots | 1.1, 14.4×11.8 | white | Guizhou province, June 2009 | (Yang et al., 2011) |
|  |  | Egg |  |  |  |  |  | Fujian province | (La Touche, 1927) |
|  | Bianchi’s Warbler *Phylloscopus valentini* | Nestling |  |  |  | 1.2, 15.8 × 12.0 | white | Guizhou province, 2018 | (Lin et al., 2024) |
|  | Brownish-flanked Bush Warbler *Cettia fortipes* | Egg | 1C+1H | 2.5, 23.4×14.4 | white with a few brown spots | 1.5, 17.7×13.0 | chocolate | Guizhou province, May 2011 | (Yang et al., 2012b) |
|  | Slaty-backed Forktail *Enicurus schistaceus* | Nestling |  |  |  | 2.6~2.7; 20.6~22.0×16.2~16.8 | white with brown spots | Eastern China | (La Touche, 1927) |
|  | Yellow-throated Bunting *Emberiza elegans* | Egg | 1C+2H | 2.7, 22.8×14.9 | white with a few brown spots | 1.4, 16.4×13.1 | offwhite with black sptos | Guizhou province, May 2011 | (Yang et al., 2012b) |
|  |  | Nestling |  |  |  |  |  |  | (Fu et al., 1984) |
|  |  | Egg | 1C+1H | 2.39±0.14, 21.45±1.03×14.29±0.24 | White with a few brown spots | 2.0±0.23, 18.44±0.70×14.71±0.55 | offwhite with black sptos | Guizhou province, June 2012 | (Su et al., 2014) |
|  | Swinhoe's White-eye *Zosterops simples* | Egg | 1C+2H | 2.39±0.14, 21.45±1.03×14.29±0.24 | White with a few brown spots | 1.09±0.09, 15.93±0.55×11.89±0.46 | white | Guizhou province, June 2012 | (Su et al., 2014) |
|  | Buff-throated Warbler *Phylloscopus subaffinis* | Egg | 1C+3H | 2.39±0.14, 21.45±1.03×14.29±0.24 | White with a few brown spots | 1.09±0.08, 15.08±0.32×11.75±0.35 | white | Guizhou province, June 2012 | (Su et al., 2014) |
|  | Collared Finchbill *Spizixos semitorques* | Egg | 1C+2H | 3.0, 21.2×16.1 | white with a few brown spots | 3.3, 24.6×17.1 | pink with violet spots | Guizhou province, June 2011 | (Yang et al., 2012b) |
|  | Large-billed Leaf Warbler *Phylloscopus magnirostris* | Fledgling |  |  |  | 16~20×12.7~13.9 | white | Weining, Guizhou, 2013 | (Yang et al., 2023) |
| Oriental Cuckoo *Cuculus optatus* | Yellow-rumped Flycatcher *Ficedula zanthopygia* | Egg |  |  | white | 1.5~2, 15.0~19.0×11.8~14.5 | white with rufous spots | Beijing | (Deng, 2013) |
|  | Siberian Stonechat *Saxicola torquata* | Nestling |  |  |  | 1.5~2.1, 16~19.8×12~15.6 | turquoise with reddish-brown spots | Eastern China | (La Touche, 1927) |
|  | Lesser Shortwing *Brachypteryx leucophrys* | Egg |  | 21.0×14.5 | turquoise with red spots | 18.5~23.0×14~15 | olive-green to sea-green with light reddish-brown freckles | Eastern China | (La Touche, 1927) |
|  | Humes’ Warbler *Phylloscopus humei* | Egg | 1C+3H | 2.5, 20.7×14.0 | white | 1.1, 15.0×11.1 | white | Gansu province, June 2002 | (Wang et al., 2004) |
|  | Pale-legged Leaf Warbler *Phylloscopus tenellipes* | Nestling |  |  |  |  |  | Jilin province | (Fu et al., 1984) |
|  |  | Egg |  |  |  | 1.5~2.3, 14~17×11.6~12.3 | white | Jilin province | (Meshcheryagina et al., 2018) |
|  | Eastern Crowned Warbler *Phylloscopus coronatus* | Nestling |  |  |  |  |  | Heilongjiang province, June 1987 | (Gao et al., 1990) |
|  |  | Egg |  |  | White | 15.5~17×12~13 | white | Beijing, 2013 | (Wang et al., 2014) |
|  |  | Nestling |  |  |  |  |  | Beijing, 2013 | (Wang et al., 2014) |
|  |  | Fledgling |  |  |  |  |  | Beijing, 2013 | (Wang et al., 2014) |
|  | White Wagtail  *Motacilla alba* | Fledgling |  |  |  | 2.4~2.8, 19.8~22.2×14.9~15.7 | white with brown spots and microgroove | Xinjiang province, July 2002 | (Yang et al., 2012b) |
|  | Yellow-bellied Prinia *Prinia flaviventris* | Egg |  | 21.0×14.7 | pink with dense red spots | 2.4~2.8, 19.8~22.2×14.9~15.7 | pink with dense red spots | Taiwan province | (La Touche, 1927) |
|  |  | Egg |  |  | white with brown spots | 13.8~15.0×11.2~12.0 | pink with dense red spots | Taiwan province | (Zhang, 1980) |
|  |  | Egg |  | 2.1, 18.86×13.83 | chocolate | 13.8~15.0×11.2~12.0 | chocolate | Taiwan province, 2010 | (Wang, 2015) |
|  |  | Fledgling |  |  |  |  |  | Yunnan province, 2021 | website 22 |
|  | Rufous-capped Babbler *Cyanoderma ruficeps* | Egg | 1C+2H |  | brown | 1.0~1.7, 14.1~17.9×11.8~13.6 | white | Taiwan province | (Lin, 2008) |
|  | *Lanceolated Warbler *Locustella lanceolata* | Egg |  |  |  | 16.2~19×12.6~14.1 | white with reddish-brown spots | Russia | (Meshcheryagina et al., 2017) |
|  | *Tree Pipit *Anthus trivialis* | Egg |  |  |  | 1.8~2.0, 14.5~17×20~23.3 | pale blue with fuchsia spots | Russia | (Payne and Kirwan, 2020b) |
|  | *Black-throated Accentor *Prunella atrogularis* | Egg |  |  |  | 17.4~21.3×13.4~16.8 | light blue | Russia | (Meshcheryagina et al., 2017) |
|  | *Daurian Redstart *Phoenicurus auroeus* |  |  |  |  | 1.7, 17.9 × 13.7 | pale turquoise with firebrick spots; white with firebrick spots | South Korea | (Kim, 2011) |
|  | *Japanese Paradise-flycatcher *Terpsiphone atrocaudata* |  |  |  |  | 17~21×14~15 | white with rufous spots; white with caesious spots | South Korea | (Kim, 2011) |
|  | *Asian Stubtail *Urosphena squameiceps* | Egg |  |  |  | 1.7~1.9, 16.5~17.0×12.5~13.5 | grey with reddish-brown spots | Japan | (Higuchi, 1998; Payne and Kirwan, 2020b) |
|  | *Arctic Warbler *Phylloscopus borealis* | Egg |  |  |  | 15~17.5×12~12.5 | white with pale pink spots | Russia | (Meshcheryagina et al., 2018) |
|  | *Common Chiffchaff *Phylloscopus collybita tristis* | Egg |  |  |  | 14~15×11~12.7 | white with brown spots | Russia | (Meshcheryagina et al., 2018) |
|  | *Yellow-browed Warbler *Phylloscopus inornatus* | Egg |  |  |  | 0.9~1.0, 12.5~16.0×11.5~12.0 | white with reddish-brown spots | Russia | (Meshcheryagina et al., 2018) |
|  | *Pallas's Leaf Warbler *Phylloscopus proregulus* | Egg |  |  |  | 0.9~1.0, 12.5~16.0×11.5~12.0 | white with reddish-brown spots | Russia | (Meshcheryagina et al., 2018) |
|  | *Dusky Warbler *Phylloscopus fuscatus* | Egg |  |  |  | 15~18×12~13 | white | Russia | (Meshcheryagina et al., 2017) |
|  | *Radde's Warbler *Phylloscopus schwarzi* | Egg |  |  |  | 1.7~2.1, 16.8~19.0×13.0~14.5 | white with brown spots | Russia | (Meshcheryagina et al., 2017) |
|  | *Greenish Warbler *Phylloscopus trochiloides* | Egg |  |  |  | 14.0~17.1×11.2~12.3 | white | Russia | (Meshcheryagina et al., 2017) |
|  | *Red-flanked Bluetail *Tarsiger cyanurus* | Egg |  |  |  | 2.0~2.5, 17.5~18×13.0~14.5 | white with reddish-brown spots | Russia | (Meshcheryagina et al., 2017) |
|  | *Common Rosefinch *Carpodacus erythrinus* | Egg |  |  |  | 18.7~22×13.2~15.2 | light turquoise with brown spots | Russia | (Meshcheryagina et al., 2017) |
|  | *Black-faced Bunting *Emberiza spodocephala* | Egg |  |  |  | 1.3~2.1, 18~21×13~16 | offwhite with reddish-brown spots | Russia | (Meshcheryagina et al., 2017) |
|  | *Tristram's Bunting *Emberiza tristrami* | Egg |  |  |  | 2.0~2.2, 15~18×18~21 | gray with dark-brown spots; light turquoise with dark-brown spots | Russia | (Meshcheryagina et al., 2017) |
|  | *Chestnut Bunting *Emberiza rutila* | Egg |  |  |  | 17.0~18.7×13.7~14.5 | white with brown spots; light blue with brown spots | Russia | (Meshcheryagina et al., 2017) |
| Lesser Cuckoo *Cuculus poliocephalus* | Brownish-flanked Bush Warbler *Horornis ffortipes* | Egg | 1C+3H | 2.6, 22.1×15.2 | chocolate | 1.5, 17.2×13.4 | chocolate | Guizhou province, July 2008 | (Yang et al., 2010a) |
|  |  | Nestling |  |  |  |  |  | Guizhou province, June 2006 | Jiang et al. 2006 |
|  |  | Nestling |  |  |  |  |  | Guizhou province, June 2004 | (Yang et al., 2012b) |
|  |  | Nestling |  |  |  |  |  | Guizhou province, June 2006 | (Yang et al., 2012b) |
|  |  | Egg | 1C+2H | 2.6, 22.1×15.2 | chocolate | 1.5, 17.7×13.0 | chocolate | Guizhou province, July 1999 | (Yang et al., 2010a) |
|  |  | Egg | 1C+3H |  | chocolate |  | chocolate | Guizhou province, July 1999 | (Yang et al., 2010a) |
|  |  | Egg | 1C+3H |  | chocolate |  | chocolate | Guizhou province, June 1999 | (Yang et al., 2010a) |
|  |  | Egg |  |  | chocolate |  | chocolate | Hubei province, July 2006 | (Yang et al., 2012b) |
|  | Eurasian Wren *Troglodytes troglodyte* | Nestling |  |  |  |  |  | Sichuan province, August 1985 | (Li, 1985) |
|  |  | Egg |  |  | chocolate | 1~1.4, 16.4~18.1×10.3~13.0 | pink with brown spots; white with reddish-brown spots | Japan | (Higuchi, 1998) |
|  | Blue-and-white Flycatcher *Cyanoptila cyanomelana* | Egg | 1C+2H |  | lightcyan | 2.3, 20.0×15.0 | white | Jilin province, 1978 | (Zhao, 1985; Zhao and He, 1981) |
|  | Manchurian Bush Warbler *Horornis canturians* |  |  |  |  |  |  | Shandong province | (Liu et al., 2025) |
|  | Aberrant Bush Warbler *Horornis flavolivaceus* | Fledgling |  |  |  | 16.1~18.5×11.8~13.1 | offwhite with chestnut spots | Sichuan province, August 2006 | (Yang et al., 2012b) |
|  | Pygmy Wren Babbler *Pnoepyga pusilla* | Egg | 1C+2H | 2.1, 20.57×15.74 | chocolate | 1.2, 18.64×13.55; 1.1, 17.93×13.49 | white | Sichuan province, 2015 | (Wang et al., 2016) |
|  | Buff-throated Warbler *Phylloscopus subaffinis* |  |  |  | chocolate | 0.7~1.2, 13.6~15.7×10.9~12.8 | white | Guizhou province | (Yang et al., 2023) |
|  | *Blyth's Leaf Warbler *Phylloscopus reguloides* | Egg |  |  | chocolate | 0.9~1.2, 13.4~16.2×11.4~12.1 | white | India | (Ali and Ripley, 1973) |
|  | *Pale-footed Bush Warbler *Hemitesia pallidipes* | Egg |  |  | chocolate | 16~18.2×12~14.0 | erythrinus; chocolate | India | (Becking, 1981) |
|  | *Tickell's Leaf Warbler *Phylloscopus affinis* | Egg |  |  | chocolate | 15~16.5×11.5~13 | white with chocolate spots | India | (Marchetti, 1992) |
| Plantive Cuckoo *Cacomantis merulinus* | Common Tailorbird *Orthotomus sutorius* | Fledgling |  |  |  |  |  | Guangdong province, July 2004 | (Yang et al., 2012b) |
|  |  | Egg |  | 1.45, 18.3×13.5 | white with light rufous spots |  | white with light rufous spots | Hong Kong | (Vaughan and Jones, 1913) |
|  |  | Fledgling |  |  |  |  |  | Guangdong province, May 2006 | (Yang et al., 2012b) |
|  |  | Nestling |  |  |  |  |  | Guangxi province, July 2004 | (Yang et al., 2012b) |
|  |  | Nestling |  |  |  |  |  | Guangxi province, April 2005 | (Yang et al., 2012b) |
|  |  | Egg | 1C+3H |  | palegreen with light rufous spots |  | pale green with light rufous spots | Guangxi province, May 2011 | (Yang et al., 2012b) |
|  |  | Egg | 1C+4H |  | white with light rufous spots |  | white with light rufous spots | Guangxi province, May 2011 | (Yang et al., 2012b) |
|  |  | Fledgling |  |  |  |  |  | Guangxi province, 2011 | (Yang et al., 2012b) |
|  |  | Fledgling |  |  |  |  |  | Guangdong province, 2023 | website 25 |
|  | Mountain Tailorbird *Phyllergates cuculatus* | Egg | 1C+2H; 1C+3H | 1.45±0.09, 19.07×13.53 | white with brown spots | 1.05±0.03 | light blue; white | Guangxi province | (Huang et al., 2015) |
|  | Dark-necked Tailorbird *Orthotomus atrogularis* | Egg | 1C+3H | 1.45±0.09, 17.8-18.5×13.0-13.7 | white with brown spots | 0.89±0.02, 14.6~16.2×10.8~12.5 | white with reddish-brown spots | Guangxi province | (Huang et al., 2015) |
|  | Striated Prinia *Prinia striata* | Fledgling |  |  |  | 1.5, 17~19×11~13 | light blue with ochre spots; white, pink, light blue or turquoise with light red, rufous or black spots | Yunnan province | (Guo et al., 2024a) |
|  | Rufescent Prinia *Prinia rufescens* | Egg |  |  | white with brown spots; blue with brown spots |  | immaculate white; white with brown spots; immaculate blue; blue with brown spots | South China | (Liang et al., 2017) |
|  | *Yellow-bellied Prinia *Prinia flaviventris* |  |  |  |  | 13.8~15.0×11.2~12.0 | pink with dense red spots | Indonesia | (Payne and Kirwan, 2020c; Well, 1999) |
|  | *Hill Prinia *Prinia superciliaris* |  |  |  |  | 14.4~18.4×11.9~13.6 | white; pink | Indian | (Payne and Kirwan, 2020c) |
|  | *Grey-breasted Prinia *Prinia hodgsonii* |  |  |  |  | 14.7×11.7 | white; blue; pinkish-white with light reddish-brown spots | Indian | (Payne and Kirwan, 2020c) |
|  | *Plain Prinia *Prinia inornata* |  |  |  |  | 0.9~1.0, 15.4~15.9×11.4~11.7 | reseda with large brown spots | Indian | (Payne and Kirwan, 2020c; Yang et al., 2023) |
|  | *Zitting Cisticola *Cisticola juncidis* |  |  |  |  | 13.8~16.8×10.2~12.3 | white with rufous spots; light blue with rufous spots | Indian | (Payne and Kirwan, 2020c) |
|  | *Crimson Sunbird *Aethopyga siparaja* |  |  |  |  | 14.3~16.3×11.2~12.0 | white with purplish brown spots; grey with purplish brown spots | Indonesia | (Payne and Kirwan, 2020c) |
| Asian Emerald Cuckoo *Chrysococcyx maculatus* | Bianchi’s Warbler *Phylloscopus valentini* | Nestling | 1C |  |  | 1.2, 15.8×12.0 | white | Guizhou province, June 2007 | (Yang et al., 2008) |
|  |  | Egg | 1C+1H | 1.5, 17.2×12.4 | white with brown spots | 1.3, 15.0×12.3 | white | Guizhou province, June 2011 | (Yang et al., 2012b) |
|  |  | Nestling | 1C |  |  |  |  | Guizhou province, June 2011 | (Yang et al., 2012b) |
|  |  | Nestling | 1C |  |  |  |  | Guizhou province, June 2011 | (Yang et al., 2012b) |
|  |  | Nestling | 1C |  |  |  |  | Guizhou province, June 2011 | (Yang et al., 2012b) |
|  |  | Nestling | 1C |  |  |  |  | Guizhou province, June 2011 | (Yang et al., 2012b) |
|  |  | Nestling | 1C |  |  |  |  | Guizhou province, June 2011 | (Yang et al., 2012b) |
|  | Chestnut-crowned Warbler *Phylloscopus castaniceps* | Egg | 1C+4H | 1.3, 17.0×12.8 | white with brown spots | 0.9, 15.0×11.1 | white | Guizhou province, June 2011 | (Yang et al., 2012b) |
|  | Blyth's Leaf Warbler *Phylloscopus reguloides* | Nestling | 1C |  |  | 1.0, 14.9 × 12.2 | White | Guizhou province, June 2011 | (Yang et al., 2012b) |
|  | Grey-crowned Warbler *Phylloscopus tephrocephalus* |  |  |  |  |  |  | Yunnan province | (Liu et al. 2025) |
|  | Buff-throated Warbler *Phylloscopus subaffinis* | Nestling |  |  |  |  |  | Sichuan province, 1985 | (Li, 1985) |
|  |  | Egg | 1C+3H | 1.5, 17.2×12.4 | white with brown spots | 0.7~1.2, 13.6~15.7×10.9~12.8 | white | Guizhou province, July 2020 | (Lin et al., 2024; Zhang et al., 2021) |
|  |  | Nestling |  |  |  |  |  | Guizhou province, July 2020 | (Zhang et al., 2021) |
|  |  | Fledgling |  |  |  |  |  | Guizhou province, June 2020 | website 26 |
|  | White-throated Fantail *Rhipidura albicollis* | Fledgling |  |  |  | 16.1~18.2×12.0~13.5 | white with brown spots | Guangxi province, July 2012 | (Jiang, 2013; Jiang et al., 2014) |
|  | White-crowned Forktail *Enicurus leschenaulti* | Egg/Fledgling | 1C+2H | 1.84 ± 0.54; 19.08×13.37 | white with brown spots | 3.61 ± 0.17; 23.55×17.61 | white with brown spots | Guizhou province, April 2018 | (Lin et al., 2024) |
|  | *Crimson Sunbird *Aethopyga siparaja* |  |  |  |  | 14.3~16.3×11.2~12.0 | white with purplish brown spots | India | (Payne, 2020b) |
|  | *Little Spiderhunter *Arachnothera longirostra* |  |  |  |  | 17.0~19.1×12.5~13.9 | creamy with zone of heavy red-brown or purple-brown spots | India | (Payne, 2020b) |
| Square-tailed Drongo-Cuckoo *Surniculus lugubris* | David's Fulvetta *Alcippe davidi* | Fledgling |  |  |  | 1.7~2.6, 18.0~20.5×14.2~15.6 | white with small reddish-brown spots | Fujian province, June 2011 | (Yang et al., 2012b) |
|  | Rufous-capped Babbler *Cyanoderma ruficeps* | Fledgling |  |  |  | 1.0~1.7, 14.1~17.9×11.8~13.6 | white; white with small brown spots and lines | Guangxi province, July 2013 | Su et al. 2016 |
|  |  | Fledgling |  |  |  |  |  | Hainan province, 2021 | website 27 |
|  |  | Fledgling |  |  |  |  |  | Guangdong province, July 2020 | unpablished data |
|  | Red-whiskered Bulbul *Pycnonotus jocosus* |  |  |  |  | 2.81, 20~24×15~18 | pink, densely covered with dark red and pale purple spots, particularly prominent at the blunt end | Guangxi province | (Liu et al. 2025) |
|  | Black Drongo *Dicrurus macrocercus* |  |  |  |  | 24~26×18~20 | white with reddish-brown spots; pink with reddish-brown spots | China | (Payne and Kirwan, 2020d) |
|  | Hair-crested Drongos *Dicrurus hottentottus* |  |  |  |  | 6~8, 25~34.5×19.8~23 | pale cream with longitudinal blotches of rather pale reddish brown, lilac or purple-red, also sparsely with deep purple freckles | China | (Payne and Kirwan, 2020d) |
|  | *Striped Tit-babbler *Mixornis gularis* |  |  |  |  | 16.6~17.1×12.7~13 | white with rufous spots | Malaysia | (Payne and Kirwan, 2020d) |
| Asian Koel *Eudynamys scolopaceus* | Black-collared Starling *Gracupica nigricollis* | Egg |  | 33.5×22.9 | sage green | 29.4×21.7 | turquoise | Hong Kong | (Vaughan and Jones, 1913) |
|  |  | Egg |  |  |  |  |  | Hong Kong, April 1931 | (Carey et al., 2001) |
|  |  | Fledgling |  |  |  |  |  | Hainan province, August 2023 | website 28 |
|  | Red-billed Blue Magpie *Urocissa erythrorhyncha* | Nestling | 1C+2H |  |  |  |  | Sichuan province, June 1963 | (Li, 1985) |
|  |  | Nestling |  |  |  |  |  | Hong Kong | (Lewthwaite, 1996) |
|  |  | Fledgling |  | 33.5×22.9 |  | 7~8, 31~36×23~24 | earthy yellow with reddish-brown spots | Guangdong province, June 2022 | website 29 |
|  | Eurasian Jay *Garrulus glandarius* |  |  |  |  | 8.5, 28.5~33.0×22.0~24.5 | Bluish-gray, green, or yellowish-green with purplish-brown, grayish-brown, or yellowish-brown spots, particularly dense at the blunt end | Guangxi province | (Liu et al. 2025) |
|  | Taiwan Blue Magpie Urocissa caerulea | Fledgling |  |  |  |  |  | Taiwan province | website 22;  website 36 |
|  | Oriental Magpie *Pica serica* | Nestling | 1C |  |  | 9~13, 23~26×32~38 | light turquoise with brown or black spots | Hong Kong | (Carey et al., 2001) |
|  |  | Nestling | 1C |  |  |  |  | Hong Kong | (Carey et al., 2001) |
|  | Azure-winged Magpie *Cyanopica cyanus* | Nestling | 2C |  |  | 6.0, 28.1×20.2 | grey with brown spots | Hubei province, August 2021 | (Lin et al., 2024); website 30 |
|  | Masked Laughingthrush *Pterorhinus perspicillatus* | Nestling | 1C |  |  | 27~28×19~21 | light cyan; light green with auburn spots | Hong Kong | (Carey et al., 2001) |
|  | House Crows *Corvus splendens* | Egg |  | 8.18±0.35, 28.08 ± 0.66×22.05 ± 0.59 | Turquoise with numerous brownish spots | 35~37×26~27 | Turquoise with numerous brownish spots | Indonesia | (Begum et al., 2011; Jadav and Parasharya, 2014) |
|  |  | Nestling |  |  |  |  |  |  | (Jadav and Parasharya, 2014) |
|  |  | Fledging |  |  |  |  |  | China | website 22 |
|  | *Long-tailed Shrike *Lanius schach* | Egg |  |  | turquoise with numerous brownish spots; celadon with numerous brownish spots | 6.5~8.1, 22.4~23.7×27.2~30.5 | pale green with rufous spots; white with rufous spots; pink with rufous spots | Thailand | (Begum et al., 2011; Limparungpatthanakij, 2020) |
|  | *Common Mynas *Acridotheres tristis* | Egg |  |  | turquoise with numerous brownish spots | 27.6~35×19.2~23.2 | turquoise | Thailand | (Begum et al., 2011) |
|  | *Great Mynas *Acridotheres grandis* |  |  | 8.18±0.35, 28.08 ± 0.66×22.05 ± 0.59 |  | 26.8~31×19.8~21.9 | blue | Thailand | (Limparungpatthanakij, 2020) |
|  | *Large-billed Crow *Corvus macrorhynchos* | Egg |  | 8.79 ± 0.83, 29.97 ± 1.16×22.97 ± 0.84 |  | 41~48.8×27.4~30.2 | dark turquoise with taupe spots | Indonesia | (Jadav and Parasharya, 2014) |
|  |  | Nestling |  |  |  |  |  |  | (Jadav and Parasharya, 2014) |
| Hodgson's Hawk-cuckoo *Hierococcyx nisicolor* | Hainan Blue Flycatcher *Cyornis hainanus* | Fledging |  | 22·5×15·4 |  |  |  | Guangxi province, Junly 2013 | (Su et al., 2016) |
|  | White-rumped Shama Copsychus malabaricus | Fledging |  |  |  | 3.05±0.20, 21.68±0.63×16.37±0.47 | turquoise with numerous brownish spots | Yunan province | website 22 |
|  | Hill Blue-flycatcher *Cyornis banyumas* | Nestling |  | 22·6×16·3 |  | 4.5~5.5, 23~24×18~19 | light turquoise | Yunnan province, June 2017 | (Luo et al., 2018) |
|  | Rufous-bellied Niltava Niltava sundara | Egg, Nestling, Fledging |  |  | chocolate | 16~19×12.9~14.2 | leather yellow | Yunnan province, July 2021 | website 31 |
|  | *Lesser Shortwing *Brachypteryx leucophris* | Egg |  | 22.6×16.3 | plain olive-brown becoming darke | 18.5~23.0×14~15 | olive-green with light reddish-brown freckles | India | (del Hoyo et al., 2020a) |
|  | *Small Niltava *Niltava macgrigoriae* | Egg |  | 22.6×16.3 | plain olive-brown becoming darke | 16~19.1×12.9~14.2 | white with dark red spots | India | (del Hoyo et al., 2020a); website 33 |
|  | *Buff-breasted Babbler *Pellorneum tickelli* | Egg |  | 22.6×16.3 | plain olive-brown becoming darke | 19.9×15.7 | light celadon with light rufous or purple grey spots | India | (del Hoyo et al., 2020a) |
|  | *Spotted Forktail *Enicurus maculatus* | Egg |  | 22.5×15.4 | uniform olive-brown to green, darker | 23~26×16~18 | laurel-green with reddish-brown spots | Myanmar | (del Hoyo et al., 2020a) |
|  | *Plumbeous Water-redstart *Phoenicurus fuliginosus* | Egg |  | 22.5×15.4 | uniform olive-brown to green, darker | 1.9~2.4, 18.6~20.0×14.2×15.2 | white with brown spots | Myanmar | (del Hoyo et al., 2020a) |
|  | *Eyebrowed Wren-babbler *Napothera epilepidota* | Egg |  | 22.5×15.4 | uniform olive-brown to green, darker |  |  | Myanmar | (del Hoyo et al., 2020a) |
|  | *Yellow-throated Fulvetta *Schoeniparus cinereus* | Egg |  | 22.5×15.4 | uniform olive-brown to green, darker | 18×14 | white with ochre spots | Myanmar | (del Hoyo et al., 2020a) |
|  | *Grey-headed Parrotbill *Paradoxornis gularis* | Egg |  | 22.5×15.4 | uniform olive-brown to green, darker |  |  | Myanmar | (del Hoyo et al., 2020a) |
|  | *Little Spiderhunter *Arachnothera longirostra* | Egg |  | 22.5×15.4 | uniform olive-brown to green, darker | 17.0~19.1×12.5~13.9 | creamy with zone of heavy red-brown or purple-brown spots | Myanmar | (del Hoyo et al., 2020a) |
|  | *Streaked Spiderhunter *Arachnothera magna* | Egg |  | 22.5×15.4 | uniform olive-brown to green, darker | 20.9~24.2×15~16.4 | caesious with brown spots | Myanmar | (del Hoyo et al., 2020a) |
| Northern Hawk-cuckoo *Hierococcyx hyperythrus* | *Eurasian Skylark *Alauda arvensis* | Egg |  | 28×20 | pale blue | 2.5~3.0, 20~27.5×15~17.5 | offwhite with black-brown spots | Japan | (del Hoyo et al., 2020b) |
|  | *Japanese Thrush *Turdus cardis* | Egg |  |  |  | 26.4×18.9 | blue with light brown spots | Japan | (del Hoyo et al., 2020b) |
|  | *Siberian Blue Robin *Larvivora cyane* | Egg |  |  |  | 2.0~2.1, 17~21×13~15.5 | sky blue; turquoise | Japan | (del Hoyo et al., 2020b) |
|  | *Red-flanked Bluetail *Tarsiger cyanurus* | Egg |  |  |  | 2.0~2.5, 17.5~18×13.0~14.5 | white with reddish-brown spots | Japan | (del Hoyo et al., 2020b) |
|  | *Asian Brown Flycatcher *Muscicapa dauurica* | Egg |  |  |  | 16.2~17.6×12.3~14.0 | offwhite; light turquoise | Japan | (del Hoyo et al., 2020b) |
|  | *Blue-and-white Flycatcher *Cyanoptila cyanomelana* | Egg |  |  |  | 1.2~2.8, 16~23×14~17 | white; white with light brown spots | Japan | (del Hoyo et al., 2020b) |
|  | *Olive-backed Pipit *Anthus hodgsoni* | Egg |  |  |  | 1.8~2.0, 14.5~17×20~23.3 | pale blue with fuchsia spots | Japan | (del Hoyo et al., 2020b) |
| Banded Bay Cuckoo *Cacomantis sonneratii* | *Common Iora *Aegithina tiphia* |  |  |  |  | 16.2~19×13.2~15 | pale cream yellow or offwite with grey stripes | India | (Payne, 2020c) |
| Violet Cuckoo *Chrysococcyx xanthorhynchus* | *Little Spiderhunter *Arachnothera longirostra* |  |  |  |  | 17.0~19.1×12.5~13.9 | creamy with zone of heavy red-brown or purple-brown spots | India | (Cheke et al., 2020) |
| Pied Cuckoo *Clamator jacobinus* | Unknown |  |  |  |  |  |  |  |  |
| Common Hawk-cuckoo *Hierococcyx varius* | Unknown |  |  |  |  |  |  |  |  |

Note: The sign "*" indicates that parasitism had been recorded outside of China, but the recorded cuckoos and hosts also breed in China, suggesting that this host is a potential host for the cuckoo in the Chinese context. The sign "#" indicates that the host is newly recorded host species. Note contents: the number of eggs or nestlings of cuckoos (C) and hosts (H) in one nest.

**Website source:**

website 1: https://mp.weixin.qq.com/s/y3EzB_IamWVX3sgGiq15-A

website 2: https://mp.weixin.qq.com/s/MSGNJGc1hH4mEw-eqBJwSg

website 3: https://h.xinhuaxmt.com/vh512/share/11667070?d=134b2f9&channel=weixin

website 4: https://www.bilibili.com/video/BV1jM411Q7fd/

website 5: https://www.douyin.com/video/7396114869298203938

website 6: https://bbs.fengniao.com/forum/11553520.html

website 7: https://mp.weixin.qq.com/s/3vSTD7dQEvFUQpqwjWkc3A

website 8: https://www.douyin.com/video/7387764611681963274

website 9: https://channels.weixin.qq.com/platform/sphdsQRJxAZjjEJ

website 10: https://mp.weixin.qq.com/s/9ktUCcDJflljJpZAI8TF0Q

website 11: https://mbd.baidu.com/newspage/data/videolanding?nid=sv_5008765907881444423&sourceFrom=qmj

website 12: https://birdnet.cn/forum.php?mod=viewthread&tid=7853282&highlight=%B6%C5%BE%E9

website 13: https://www.bilibili.com/video/av927043270/?p=1

website 14: https://mp.weixin.qq.com/s/2WdM6x_dNPStCgcHO9UvYQ

website 15: https://www.bilibili.com/video/BV1Qu411n7XT/

website 16: https://www.kepu.net.cn/blog/public/201904/t20190401_477950.html

website 17: https://baijiahao.baidu.com/s?id=1751890423548631575&wfr=spider&for=pc

website 18: https://mp.weixin.qq.com/s/SJ1lobb9ib71d6Ko0jyKcQ

website 19: https://birdnet.cn/forum.php?mod=viewthread&tid=4761716&highlight=%C8%B5%F0%B6%2B%B6%C5%BE%E9

website 20: https://birdnet.cn/thread-7420435-1-1.html

website 21: https://www.birdnet.cn/forum.php?mod=viewthread&tid=7828760&highlight=%B6%C5%BE%E9

website 22: https://mp.weixin.qq.com/s/SJ1lobb9ib71d6Ko0jyKcQ

website 23: https://www.birdnet.cn/thread-2409798-1-1.html

website 24: https://www.birdnet.cn/thread-4056469-1-1.html

website 25: https://mp.weixin.qq.com/s/RY6QoARzLZQPkiinwUPd4w

website 26: https://www.birdnet.cn/forum.php?mod=viewthread&tid=7785944&highlight=%B4%E4%BD%F0%BE%E9

website 27: https://mp.weixin.qq.com/s/fhGZGNepXQysurPvZiMq-g

website 28: https://m.gmw.cn/2023-08/29/content_1303498415.htm

website 29: https://mp.weixin.qq.com/s/KQC9RrVprrKDa-uZt1Ynnw

website 30: https://www.bilibili.com/video/BV1qg411z7DT/

website 31: https://mp.weixin.qq.com/s/r_HL1ADnkd0Jc5vA35Ig6A

website 32: https://wap.sciencenet.cn/blog-2048045-1296278.html

website 33: https://s.weibo.com/weibo?q=%E5%A4%A7%E9%B9%B0%E9%B9%83&nodup=1&page=6

website 34: https://s.weibo.com/weibo?q=%E4%B8%AD%E6%9D%9C%E9%B9%83%E9%B8%9F%E7%B1%BB&page=46

website 35: https://mp.weixin.qq.com/s/b9fDxn-KIicOTTZSjYebVQ

website 36:

https://ebird.org/checklist/S58482677?_gl=1*vbfuhu*_gcl_au*MTUzMDA4NTM3NS4xNzQyNjQxNzE4*_ga*MjExMTYzNDExMC4xNzM0ODUzNDE0*_ga_QR4NVXZ8BM*czE3NDY2MzI5ODMkbzk2JGcxJHQxNzQ2NjMzMDIwJGoyMyRsMCRoMA..&_ga=2.124103050.108023473.1746623824-2111634110.1734853414

**References:**

Ali S, Ripley SD (1973). ‘Handbook of the birds of India and Pakistan. Vol.8’. (Oxford Universty Press: London, UK)

Becking JH (1981). Notes on the breeding of Indian cuckoos. *Journal of the Bombay Natural History Society* **782**, 201–231.

Begum S, Moksnes A, Røskaft E, Stokke BG (2011). Interactions between the Asian koel (*Eudynamys scolopacea*) and its hosts. *Behaviour* **148**, 325–340.

Bu F, Zhang L, Ren B (1999). Breeding ecology of Indian Cuckoo in Xiaodian area of Taiyuan city, Shanxi Province. *Shanxi Forestry Science and Technology* **3**, 36–38.

Carey GJ, Chalmers ML, Diskin DA, Kennerley PR, Leader PJ, Leven MR, Lewthwaite RW, Melvilee DS, Turnbull M, Young L (2001). ‘The avifauna of Hong Kong’. (Hong Kong Bird Watching Society: Hong Kong, China)

Cheke R, Mann C, Kirwan GM (2020). Little Spiderhunter (*Arachnothera longirostra*), version 1.0. In ‘Birds of the World’. (Cornell Lab of Ornithology: Ithaca, NY, USA) Available at: https://birdsoftheworld.org/bow/species/litspi1/cur/introduction

Cheng T (1973). ‘Avifauna of Qingling Mountain’. (Science Press: Beijing, China)

Cheng T (1963). ‘Resource fauna of China. Aves.’ (Science Press: Beijing, China)

Cheng T, Xian Y, Guan G (1991). ‘Fauna Sinica: Aves, Columbiformes, Psittaciformes, Cuculiformes and Strigiformes’. (Science Press: Beijing, China)

Clement P, Christie DA (2020). Hainan Blue Flycatcher (*Cyornis hainanus*), version 1.0. In ‘Birds of the World’. (Cornell Lab of Ornithology: Ithaca, NY, USA)

Collar N (2020). Chestnut-bellied Rock-Thrush (*Monticola rufiventris*), version 1.0. In ‘Birds of the World’. (Cornell Lab of Ornithology: Ithaca, NY, USA)

Deng W (2013). Brood parasitism on the Yellow-rumped Flycatcher (*Ficedula zanthopygia*) by the Oriental Cuckoo (*Cuculus optatus*) in an artificial nestbox in Beijing. *Chinese Birds* **4**, 187–188.

Erritzøe J, Mann CF, Brammer FP, Fuller RA (2012). ‘Cuckoos of the World’. (Christopher Helm: London, UK)

Fan L, Liu R, Song Q (2000). Breeding ecology of Large-Hawk cuckoo in Lishan Nature Reserve. *Sichuan Journal of Zoology* **19**, 85–86.

Fu T, Gao W, Song Y (1984). ‘Birds of Changbai Mountain’. (Northeastern China Normal University Press: Jilin)

Gao G, Yuan Q, Huang M, Liang D, Li B, Luo X (2024). Brood Parasitism on the Streak-Breasted Scimitar Babbler *Pomatorhinus ruficollis* by the Large Hawk Cuckoo *Hierococcyx sparverioides* Was Found in the Gaoligong Mountains, Yunnan, China. *Chinese Journal of Zoology* **59**, 981–986.

Gao W (2004). ‘The Ecology of Cavity-nesting Birds of Northeastern China’. (Jilin Science and Technology Press: Jilin)

Gao W, Yang Z, Luo W (1990). Observation on breeding behavior of three bird species. *Chinese Journal of Wildlife* **4**, 10–11.

Grim T, Samaš P, Procházka P, Rutila J (2014). Are tits really unsuitable hosts for the Common Cuckoo? *Ornis Fennica* **91**. doi:10.51812/of.133853

Guangdong Institute of Entomology, Sun Yat-sen University (1983). ‘Birds and Mammals of Hainan Island’. (Science Press: Beijing, China)

Guo L, Liu J, Liang W (2024). Brood Parasitism on Himalayan Prinia *Prinia crinigera* by Plaintive Cuckoo *Cacomantis merulinus* in Yunnan, Southwestern China. *Chinese Journal of Zoology* **59**, 815–816.

Guo W, Hu Z, Lin B, Kuang Y, Cao H, Wang C (2022). Nest site selection and breeding ecology of the red-billed blue magpie *Urocissa erythrorhyncha* in central China. *Animal Biology* **72**, 153–164.

Hao S, Wang Y (1992). Breeding ecology of the Oriental Reed Warbler. *Shandong Forestry Science and Technology* **1**, 20–22.

Higuchi H (1998). Host Use and Egg Color of Japanese Cuckoos. In ‘Parasitic Birds and Their Hosts, Studies in Coevolution’. (Eds SI Rothstein, SK Robinson.) (Oxford University Press: Oxford, United Kingdom)

del Hoyo J, Collar N, Kirwan GM (2020a). Hodgson’s Hawk-Cuckoo (*Hierococcyx nisicolor*), version 1.0. In ‘Birds of the World’. (Cornell Lab of Ornithology: Ithaca, NY, USA)

del Hoyo J, Collar N, Kirwan GM (2020b). Northern Hawk-Cuckoo (*Hierococcyx hyperythrus*), version 1.0. In ‘Birds of the World’. (Cornell Lab of Ornithology: Ithaca, NY, USA)

Hu Y, Wang X, Chang H, Sun Y (2013). Brood Parasitism on Elliot’s Laughingthrush by Large Hawk Cuckoo. *Chinese Journal of Zoology* **48**, 292–293.

Huang Q, Wang L, Yang C, Liang W (2015). Brood Parasitism on Two Tailorbird Hosts (*Orthotomus spp.*) by Plaintive Cuckoo (*Cacomantis merulinus*). *Chinese Journal of Zoology* **50**, 790–794.

Huo J, Su T, Yang C, Liang W (2014). Brood Parasitism and Egg Mimicry on *Garrulax canorus* and *Babax lanceolatus* by *Clamator coromandus*. *Sichuan Journal of Zoology* **33**, 337–341.

Huo J, Su T, Yao X, Yang C, Liang W (2016). Brood Parasitism on White-bellied Redstart (*Hodgsonius phaenicuroides*) by Large Hawk-cuckoo (*Cuculus sparverioides*). *Chinese Journal of Zoology* **51**, 1101–1105.

Jadav PCA, Parasharya BM (2014). Intensity of Brood Parasitism of Asian Koel (*Eudynamys scolopacea*) in the Nest of House Crow (*Corvus splendens*) and Jungle Crow (*Corvus macrorhynchos*) in Anand Region of Gujarat, India. *Trends in Biosciences* **7**, 4471–4476.

Jia C, Liang W, Gong H (2007). Chestnut-winged Cuckoo parasitized the Hwamei. *Chinese Journal of Zoology* **42**, 38.

Jiang A (2013). Brood parasitism on White-throated Fantail (*Rhipidura albicollis*) by Asian Emerald Cuckoo (*Chrysococcyx maculatus*) in Guangxi, southwestern China. *Journal of Hainan Normal University (Natural Science)* **26**, 198–199.

Jiang A, Zhou F, Liu N (2014). Significant recent ornithological records from the limestone area of south-west Guangxi, south China, 2004–2012. *Forktail* **30**, 122–129.

Jiang Y, Liang W, Yang C, Sun Y (2007). Large Hawk-cuckoo parasitized the White-browed Laughingthrush. *Sichuan Journal of Zoology* **26**, 509.

Kim Y (2011). A study on the breeding ecology of *Terpsiphone atrocaudata* on Jeju Island, Korea. Ph.D. thesis, Jeju National University Jeju, Korea.

La Touche JDD (1927). ‘A Handbook of the Birds of Eastern China’. (Taylor and Francis: London)

Lewthwaite RW (1996). Forest Birds of Southeastern China: Observations during 1984–1996. Hong Kong Bird Report, Hong Kong.

Li G (1985). ‘Fauna of Sichuan Province. Vol.3: Birds’. (Sichuan Science and Technology Press: Chengdu, China)

Liang W, Møller AP, Stokke BG, Yang C, Kovařík P, Wang H, Yao C-T, Ding P, Lu X, Moksnes A, Røskaft E, Grim T (2016). Geographic variation in egg ejection rate by great tits across 2 continents. *Behavioral Ecology* **27**, 1405–1412.

Liang W, Yang C, Takasu F (2017). How can distinct egg polymorphism be maintained in the rufescent prinia (*Prinia rufescens*)–plaintive cuckoo (*Cacomantis merulinus*) interaction—a modeling approach. *Ecology and Evolution* **7**, 5613–5620.

Limparungpatthanakij WL (2020). Asian Koel (*Eudynamys scolopaceus*), version 1.0. In ‘Birds of the World’. (Cornell Lab of Ornithology: Ithaca, NY, USA)

Lin R (2008). Observation of Oriental Cuckoo parasitism on Rufous-capped Babbler in central Taiwan. *Natural Conservation Quarterly* **64**, 58–62.

Lin S, Guo S, Hao L, He L, Liang W (2024). New Host Species Recorded for Four Parasitic Cuckoo Species in China. *Chinese Journal of Zoology* **59**, 632–635.

Liu D, Xin M, Wang X (1992). Study on migration rules and feeding habits of Cuculiformes in Qingdao area. *Shandong Forestry Science and Technology* **1**, 25–26.

Liu H, Feng J, Su H (1984). Observation on egg-laying of the common cuckoo. *Sichuan Journal of Zoology* **3**, 14–16.

Liu H, Su H, Shen S, Lan Y, Ren J, Wu W (1988). Breeding ecology of the Eurasian Wren in Guandi Mountain, Shanxi Province. *Chinese Journal of Zoology* **23**, 8–12.

Liu J, Lin S, Liang W (2025). An updated list of parasitic cuckoos and their hosts in China. *Avian Research* **16**, 100249.

Liu X, Long G (1986). Breeding behavior of the Light-vented Bulbul. *Chinese Journal of Zoology* **5**, 12–15.

Liu X, Zhong G, Zhang Y, He G, Wang L, Liang W (2022). A Parasitism Case of the White Wagtail (*Motacilla alba*) Nest from Common Cuckoo (*Cuculus canorus*). *Chinese Journal of Wildlife* **43**, 725–730.

Lu X (1988). Common cuckoo parasitism on the oriental reed warbler. *Sichuan Journal of Zoology* **7**, 21–22.

Luo K, Guan S, Lu ZY, Zhao H, Li DL (2018). Brood Parasitism on Hill Blue-flycatcher (*Cyornis banyumas*) by Whistling Hawk Cuckoo (*Hierococcyx nisicolor*) in Yunnan, Southwestern China. *Chinese Journal of Zoology* **53**, 125, 142.

Ma L (2018). Comparison of counteradatations in four sympatric host species to defense against cuckoo parasitism. Ph.D. thesis, Hainan Normal University Haikou, China.

Ma W, Liu N, Ding W, Wang L, Bo X (2012). Brood Parasitism on *Lanius isabellinus* by *Cuculus canorus*. *Sichuan Journal of Zoology* **31**, 74–76.

Marchetti K (1992). Costs to host defence and the persistence of parasitic cuckoos. *Proceedings of the Royal Society of London. Series B: Biological Sciences* **248**, 41–45. doi:10.1098/rspb.1992.0040

Meshcheryagina S, Gennadiy B, Bourski O (2017). Distribution of the Oriental Cuckoo gentes in Russia: a review of brood parasitism records by the host species. *Fauna of the Urals and Siberia* **2**, 39–163.

Meshcheryagina SG, Mashanova A, Bachurin GN, Mitiay IS, Golovatin MG (2018). Host species determines egg size in Oriental cuckoo. *Journal of Zoology* **306**, 147–155.

Payne RB (2020a). Asian Emerald Cuckoo (*Chrysococcyx maculatus*), version 1.0. In ‘Birds of the World’. (Cornell Lab of Ornithology: Ithaca, NY, USA)

Payne RB (2020b). Banded Bay Cuckoo (*Cacomantis sonneratii*), version 1.0. In ‘Birds of the World’. (Cornell Lab of Ornithology: Ithaca, NY, USA)

Payne RB (2020c). Indian Cuckoo (*Cuculus micropterus*), version 1.0. In ‘Birds of the World’. (Cornell Lab of Ornithology: Ithaca, NY, USA)

Payne RB, Kirwan GM (2020a). Large Hawk-Cuckoo (*Hierococcyx sparverioides*), version 1.0. In ‘Birds of the World’. (Cornell Lab of Ornithology: Ithaca, NY, USA)

Payne RB, Kirwan GM (2020b). Oriental Cuckoo (*Cuculus optatus*), version 1.0. In ‘Birds of the World’. (Cornell Lab of Ornithology: Ithaca, NY, USA)

Payne RB, Kirwan GM (2020c). Plaintive Cuckoo (*Cacomantis merulinus*), version 1.0. In ‘Birds of the World’. (Cornell Lab of Ornithology: Ithaca, NY, USA)

Payne RB, Kirwan GM (2020d). Square-tailed Drongo-Cuckoo (*Surniculus lugubris*), version 1.0. In ‘Birds of the World’. (Cornell Lab of Ornithology: Ithaca, NY, USA)

Qian Y, Zhang J (1965). ‘Birds and Mammals of Southern Xinjiang’. (Science Press: Beijing, China)

Su T, Huo J, Yang C, Liang W (2017). Brood Parasitism on Barn Swallow (*Hirundo rustica*) Populations in China by Common Cuckoo (*Cuculus canorus*). *Chinese Journal of Zoology* **52**, 338–341.

Su T, Huo J, Yang C, Liang W (2014). Brood Parasitism on Three Host Species by Himalayan Cuckoo. *Chinese Journal of Zoology* **49**, 505–510.

Su T, Jiang A, Liang W (2016). New Host Records of Whistling Hawk Cuckoo and Drongo Cuckoo. *Chinese Journal of Zoology* **51**, 1142–1143.

Tian F, Song Y, Hao S, Feng Z, Wang Y (1991). Notes on ecology of the common cuckoo in Nansi Lake. *Shandong Forestry Science and Technology* **1**, 9–12.

Vaughan RE, Jones KH (1913). The Birds of Hong Kong, Macao, and the West River or Si Kiang in South-eastern China, with special reference to their Nidification and Seasonal Movements. *Ibis* **55**, 163–200.

Wang H, Jiang Y, Gao W (2011). Jankowski’s bunting (*Emberiza jankowskii*): current status and conservation. *Chinese Birds* **1**, 251–258.

Wang J (2012). On the Ecology of Indian Cuckoo *Cuculus micropterus* in Luya National Nature Reserve, Shanxi Province. *Chinese Journal of Wildlife* **33**, 184–186.

Wang L (2015). Comparison of counter-adaptation in two sympatric prinias to defense against cuckoo parasitism. Doctoral dissertation, Wuhan University Wuhan, Chian.

Wang P, Huang X, Dong L, Zhang Z (2014). The Multiple Brood Parasitism of Eastern Crowned Warbler by Oriental Cuckoo in Xiaolongmen National Forest Park of Beijing. *Chinese Journal of Zoology* **49**, 511–515.

Wang P, Yang A, Zhang Z, Fu Y (2016). The Brood Parasitism of Pygmy Wren Babbler (*Pnoepyga pusilla*) by Lesser Cuckoo (*Cuculus poliocephalus*). *Chinese Journal of Zoology* **51**, 319–322.

Wang Y (2018). Nest Parasitism on Siberian Stonechat by Common Cuckoo in Saihanba Forest Region. *Chinese Journal of Wildlife* **39**, 699–701.

Wang Z, Jia C, Sun Y (2004). Parasitized breeding and nestlings growth in Oriental Cucko. *Chinese Journal of Zoology* **39**, 103–105.

Well DR (1999). ‘The Birds of the Thai-Malay Peninsula. Vol. 1, Non-passerines’. (Academic Press: New York, USA)

Yan A (1985). Observation on Indian Cuckoo. *Chinese Biological Bulletin* **3**, 13.

Yang C, Antonov A, Cai Y, Stokke BG, Moksnes A, Røskaft Ei, Liang W (2012a). Large Hawk‐Cuckoo *Hierococcyx sparverioides* parasitism on the Chinese Babax *Babax lanceolatus* may be an evolutionarily recent host–parasite system. *Ibis* **154**, 200–204.

Yang C, Cai Y, Liang W (2008). Asian Emerald Cuckoo parasitized the Bianchi’s Warbler *Seicercus valentini*. *Chinese Journal of Zoology* **43**, 74–75.

Yang C, Cai Y, Liang W (2010a). Brood parasitism and egg mimicry on Brownish-flanked Bush Warbler (*Cettia fortipes*) by Lesser Cuckoo (*Cuculus poliocephalus*). *Zoological Research* **31**, 555−560.

Yang C, Cai Y, Liang W (2011). Visual modeling reveals cryptic aspect in egg mimicry of Himalayan Cuckoo (*Cuculus saturatus*) on its host Blyth’s Leaf Warbler (*Phylloscopus reguloides*). *Zoological Research* **32**, 451–455.

Yang C, Lan H, Yao X, Yu D (2023). ‘Cuckoos and Their Hosts in Kuankuoshui National Nature Reserve of Guizhou’. (China Forestry Publishing House: Beijing, China)

Yang C, Liang W, Antonov A, Cai Y, Stokke BG, Fossøy F, Moksnes A, Røskaft E (2012b). Diversity of parasitic cuckoos and their hosts in China. *Chinese Birds* **3**, 9–32.

Yang C, Liang W, Cai Y, Shi S, Takasu F, Møller AP, Antonov A, Fossøy F, Moksnes A, Røskaft E, Stokke BG (2010b). Coevolution in Action: Disruptive Selection on Egg Colour in an Avian Brood Parasite and Its Host. *PLoS ONE* **5**, e10816.

Yang C, Su T, Liang W, Møller AP (2015). Coevolution between the large hawk-cuckoo (*Cuculus sparverioides*) and its two sympatric Leiothrichidae hosts: evidence for recent expansion and switch in host use? *Biological Journal of the Linnean Society* **115**, 919–926.

Yi T (2020). Coevolution between large hawk cuckoos and its hosts. A Dissertation Submitted for the Degree of Ph.D, Hainan Normal University Haikou, China.

Zhang J (2001). Observation on the breeding habits of *Dicrurus macrocercus*. *Chinese Journal of Zoology* **36**, 60–63.

Zhang S, Zhang S, Yan F (2017). Investigation on the parasitic breeding habit of *Cuculus micropterus* and *Cuculus canorus bakeri*. *Shandong Forestry Science and Technology* **47**, 68–69.

Zhang T (1989). Studies on breeding ecology of the common cuckoo. *Shandong Forestry Science and Technology* **1**, 24–26.

Zhang W (1980). ‘A Field Guide to the Birds of Taiwan’. (Insititue of Environmental Science, Tunghai University: Taiwan, China)

Zhang Y, Zhong G, He G, Liu X, Wang L, Liang W (2021). One nest of Buff-throated Warbler was parasitized by Asian Emerald Cuckoo in Guizhou. *Sichuan Journal of Zoology* **40**, 446–447.

Zhang Y, Zhong G, Wan G, Wang L, Liang W (2023). Brood parasitism and egg recognition in three bunting hosts of the cuckoos. *Ecology and Evolution* **13**, e10659.

Zhao Z (1985). ‘The Avifauna of Changbai Mountain’. (Jilin Science and Technology Press: Changchun, China)

Zhao Z, He J (1981). Studies on the breeding biology of blue-and-white flycatcher. *Acta zoologica sinica* **27**, 388–394.

**Supplementary Information 2** The phylogenetic tree of host species


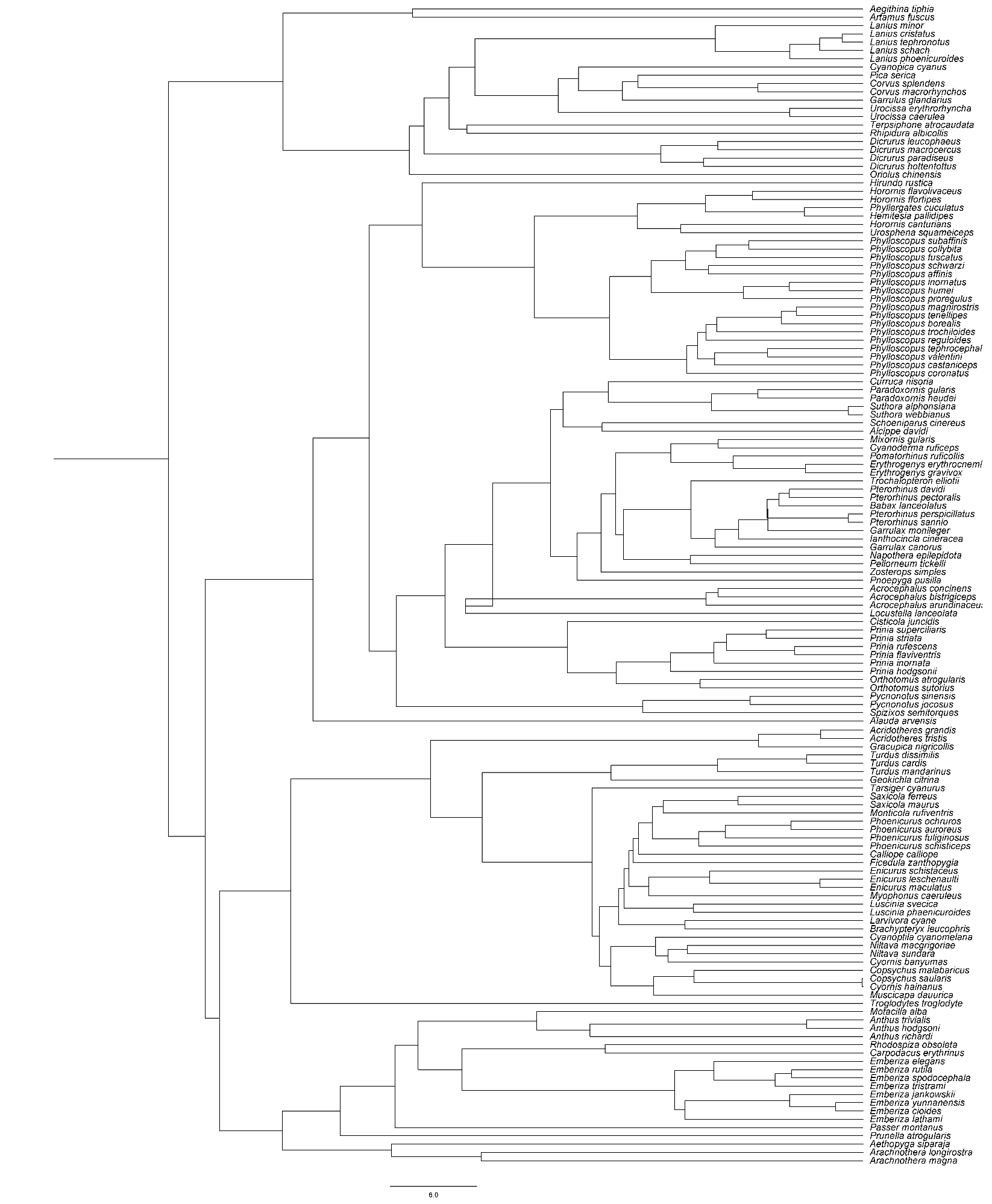


**Supplementary Information 3** The real-time tracking program for parasitism records


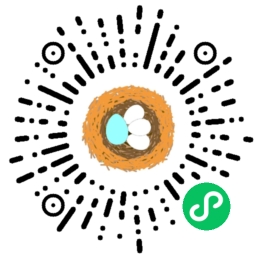


To access and use the program, scan the QR code above with the WeChat app.
